# Synthesis and Structure–Activity Relationship (SAR) Studies on New 4-Aminoquinoline-Hydrazones and Isatin Hybrids as Promising Antibacterial Agents

**DOI:** 10.3390/molecules29235777

**Published:** 2024-12-06

**Authors:** Ayesha Ubaid, Mohd. Shakir, Asghar Ali, Sobia Khan, Jihad Alrehaili, Razique Anwer, Mohammad Abid

**Affiliations:** 1 Medicinal Chemistry Laboratory, Department of Biosciences, Jamia Millia Islamia, New Delhi 110025, India; ashislam98@gmail.com (A.U.); shakirsalim7534@gmail.com (M.S.); asgharali@jamiahamdard.ac.in (A.A.); writetosobia25@gmail.com (S.K.); 2 Clinical Biochemistry Laboratory, Department of Biochemistry, School of Chemical and Life Science, Jamia Hamdard, New Delhi 110062, India; 3 Department of Pathology, College of Medicine, Imam Mohammad Ibn Saud Islamic University (IMSIU), Riyadh 13317-4233, Saudi Arabia; jaalrehaili@imamu.edu.sa

**Keywords:** antimicrobial resistance *S. aureus*, MIC, quinoline–isatin hybrids, biofilm, bactericidal, MDR

## Abstract

In response to the escalating crisis of antimicrobial resistance (AMR), there is an urgent need to research and develop novel antibiotics. This study presents the synthesis and assessment of innovative 4-aminoquinoline-benzohydrazide-based molecular hybrids bearing aryl aldehydes (**HD1-23**) and substituted isatin warheads (**HS1-12**), characterized using multispectroscopic techniques with high purity confirmed by HRMS. The compounds were evaluated against a panel of clinically relevant antibacterial strains including the Gram-positive *Enterococcus faecium*, *Bacillus subtilis*, and *Staphylococcus aureus* and a Gram-negative *Pseudomonas aeruginosa* bacterial strain. Preliminary screenings revealed that several test compounds had significant antimicrobial effects, with **HD6** standing out as a promising compound. Additionally, **HD6** demonstrated impressively low minimum inhibitory concentrations (MICs) in the range of (8–128 μg/mL) against the strains *B. subtilis, S. aureus* and *P. aeruginosa*. Upon further confirmation, **HD6** not only showed bactericidal properties with low minimum bactericidal concentrations (MBCs) such as (8 μg/mL against *B. subtilis*) but also displayed a synergistic effect when combined with the standard drug ciprofloxacin (CIP), highlighted by its FICI value of (0.375) against *P. aeruginosa*, while posing low toxicity risk. Remarkably, **HD6** also inhibited a multidrug-resistant (MDR) bacterial strain, marking it as a critical addition to our antimicrobial arsenal. Computation studies were performed to investigate the possible mechanism of action of the most potent hybrid **HD6** on biofilm-causing protein (PDB ID: 7C7U). The findings suggested that **HD6** exhibits favorable binding free energy, which is supported by the MD simulation studies, presumably responsible for the bacterial growth inhibition. Overall, this study provides a suitable core for further synthetic alterations for their optimization as an antibacterial agent.

## 1. Introduction

Antibiotics revolutionized medicine by allowing complex procedures and effectively treating infectious diseases. However, their misuse has led to resistance and escalation of multidrug-resistant pathogens, presenting significant challenges to healthcare systems [1]. Over the years, improper and excessive use of antibiotics in human medicine, veterinary practices, and agriculture has increased the spread of resistance genes, contributing to what has been termed as the “Silent Pandemic” [2]. Projections suggest that by 2050, antimicrobial resistance (AMR) could lead to an annual death toll of 10 million, making it a leading cause of mortality worldwide [3]. The highest projected fatalities are expected in Asia, followed by Africa, primarily due to large populations and insufficient regulation for AMR prevention [4]. Currently, bacterial infections are responsible for approximately 7.7 million deaths annually [5], with 4.95 million of these deaths linked to drug-resistant pathogens and 1.27 million directly attributed to infections by antibiotic-resistant bacteria [6]. A lack of understanding about antimicrobials significantly contributes to AMR. A survey in India revealed that 49% of respondents believed antibiotics could treat viral infections, and 45% used them for colds. Consequently, India has one of the highest rates of infectious diseases, including those caused by multiresistance pathogens [7]. The increasing prevalence of antibiotic resistance coupled with a stagnation in the development of new antimicrobial agents poses a critical threat to public health. The ESKAPE pathogens—*E. faecium*, *S. aureus*, *Klebsiella pneumoniae*, *Acinetobacter baumannii*, *P. aeruginosa*, and *Enterobacter* species—have been identified as priority multidrug-resistant organisms for which urgent therapeutic options are needed [8]. Despite the recent introduction of several novel antibiotics and antibiotic adjuvants, including advanced β-lactamase inhibitors [9], these pathogens continue to present significant therapeutic challenges as we progress into the third decade of the twenty-first century. The increasing prevalence of antibiotic resistance has raised significant concerns about the efficacy of existing treatments, underscoring the urgent need for the development of new and improved antibacterial agents.

Heterocycles are attracting increasing research attention due to their roles in medicine [10], antimicrobial treatments, and industry. Statistics indicate that over 85% of biologically active chemical entities contain a heterocycle [11]. These compounds consist of cyclic structures with at least one heteroatom. Due to their abundance in macromolecules such as vitamins, biological active chemicals, enzymes, and natural products, they are regarded as key constituents in medicinal chemistry [12,13]. Various compound classes have played a key role in developing antimicrobial drugs, with heterocyclic derivatives standing out as vital class of therapeutic agents for treating infectious diseases.

Quinoline, also known as 1-aza-naphthalene or benzo[b]pyridine, is a highly popular heterocyclic aromatic compound with a wide range of applications in commercial and medicinal chemistry [14]. It contains nitrogen and is known for its synthetic flexibility. Various studies have shown that quinoline hydrazide/hydrazone derivatives possess several biological activities, such as antimalarial [12,15], antibacterial [16,17,18], antifungal, anti-inflammatory [19,20], and anticancer [21]. Some notable drugs include Lenvatinib, an anticancer drug that acts as a tyrosine kinase inhibitor [22]; Tipifarnib, used in leukemia treatment as a farnesyl transferase inhibitor [23,24]; Bedaquiline, used for TB treatment by inhibiting ATP synthesis [25,26]; Saquinavir, an inhibitor targeting proteases for HIV-1 [27]; Pitavastatin, which lowers cholesterol and prevents heart disease as an HMG-CoA reductase inhibitor [28,29]; Fluoroquinolones, which works as a DNA replication inhibitor and is used as an antimicrobial agent [30,31]; and Chloroquine, a heme polymerization inhibitor used against plasmodium [32]. The structural versatility of the quinoline ring has facilitated the development of diverse quinoline hydrazide/ hydrazone derivatives, enabling them to target various bacterial mechanisms such as DNA gyrase [33], glucosamine-6-phosphate synthase, enoyl ACP reductase, and 3-ketoacyl ACP reductase [34].

Isatin or 1H-indole-2,3-dione, also known as indole quinine and indanedione, is a nitrogen-containing heterocyclic pharmacophore known for its broad biological activity, making it a bioactive heterocyclic moiety [35]. Derivatives of isatin exhibit various biological properties, including anticancer effects in different cancer types, as well as antianxiety [36], anxiogenic, anti-inflammatory [37], anticonvulsant, antibacterial, antimalarial [38], antifungal, antidiabetic, and antioxidant effects [39]. Being “privileged building blocks”, isatin moieties can be modified at almost any position. This versatility and broad spectrum of biological activities has encouraged much research to study isatins, resulting in a vast array of structurally diverse derivatives [40]. Significant chemical modifications typically occur at the N-1, C-3, and C-5 positions of the isatin moiety [41].

**Figure 1:**
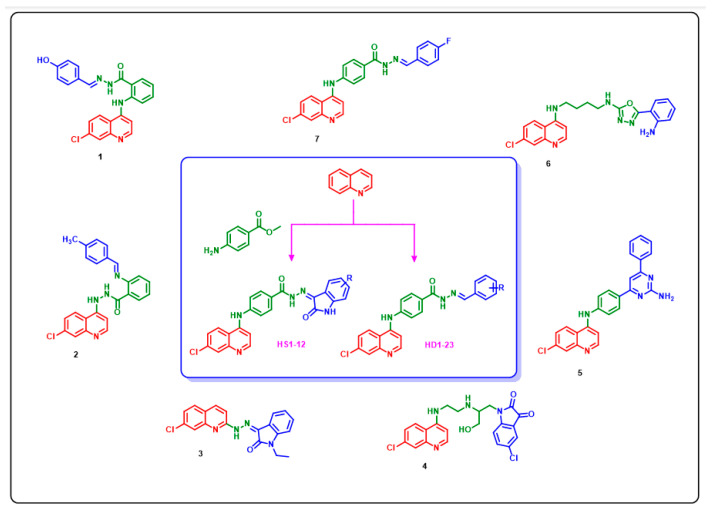
The rationale for the design and synthesis of 4-aminoquinoline–isatin hybrids and Schiff base molecules as antimicrobial agents. 1, 2, 3, 4, 5, 6, and 7 represent previously studied molecules with different biological activities [42,43,44,45,46,47,48].

In light of this escalating antimicrobial resistance, our study aims to contribute to the search for new antibacterial agents. The rationale for our study of the synthesized compounds is demonstrated in Figure 1, which provides a visual representation of the structural and functional diversity of various biologically active compounds [42,43,44,45,46,47,48]. We employed a molecular hybridization strategy to synthesize and evaluate novel antibacterial compounds derived from 4-aminoquinoline and isatin derivatives. This innovative approach enables the combination of multiple bioactive pharmacophores, such as the chloroquinoline core, hydrazone linkages, and isatin units and substituents, into a single molecular framework, potentially enhancing their therapeutic efficacy against resistant bacterial strains. A total of 35 new compounds were synthesized and thoroughly characterized using various spectroscopic techniques. Antibacterial activity was assessed in vitro against key pathogens, including *E. faecalis*, *B. subtilis*, *S. aureus*, and *P. aeruginosa*. Among the synthesized compounds, **HD6** exhibited potent antibacterial properties. We conducted comprehensive antimicrobial assessments including minimum inhibitory concentration (MIC), minimum bactericidal concentration (MBC), and zone of inhibition (ZOI) assays. Notably, **HD6** demonstrated synergistic effects in combination with ciprofloxacin, indicating enhanced antibacterial activity. Additionally, a hemolytic activity assay was performed to evaluate the compound’s safety profile on human red blood cells, revealing a favorable toxicity level. The results collectively suggest that the aminoquinoline-hydrazone derivative **HD6** possesses significant antibacterial efficacy with a promising safety profile, indicating its potential as a therapeutic agent against antibiotic-resistant infections.

## 2. Results

### 2.1. Chemistry

**Scheme 1:**
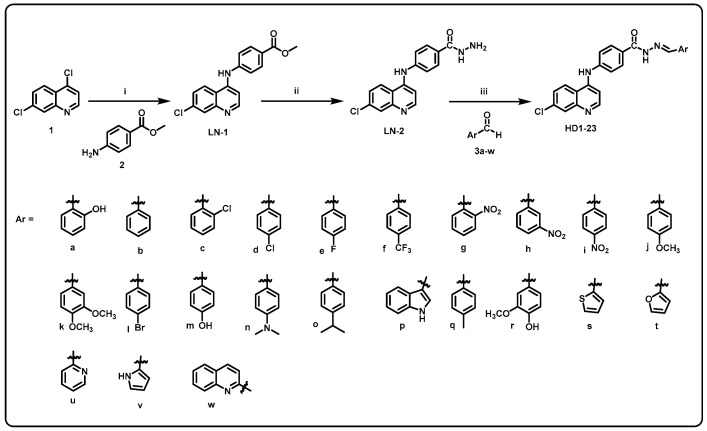
Synthesis of 4-aminoquinoline-hydrazones. Reagents and conditions: (i) Methyl4-aminobenzoate, ethanol, p-TSA, 80 °C, 48 h. (ii) Hydrazine hydrate, ethanol, reflux at 80 °C, 24 h. (iii) Substituted benzaldehyde, glacial acetic acid, ethanol, reflux at 80 °C, overnight. (a–w) Synthesized substituted derivatives of HD.

Synthesis of *(E)-N*′-benzylidene-4-((7-chloroquinolin-4-yl)amino)benzohydrazide-based molecular hybrids connected through aromatic linker (LN-2) was achieved using the approach as show in Scheme 1. Briefly, commercially available 4,7-dichloroquinoline 1 was heated with methyl 4-aminobenzoate 2 at 80 °C for 48 h in presence of ethanol and *p*-Toluene sulfonic acid as a catalyst to give the linker LN-1 in quantitative yield. The LN-1 linker was heated at 80 °C with hydrazine hydrate for 24 h in the presence of ethanol as a solvent to form LN-2 hydrazide as an intermediate. Finally, the linker LN-2 (4-((7-chloroquinolin-4-yl)amino)benzohydrazide) was reacted with substituted aromatic/heteroaromatic benzaldehyde (3a-w) in ethanol under reflux overnight to give the corresponding *(E)-N*′-benzylidene-4-((7-chloroquinolin-4-yl)amino)benzohydrazide molecular hybrids (**HD1-23**). All the final compounds were characterized by multispectroscopic techniques such as ^1^H, ^13^C-NMR, FT-IR, and mass spectrometry. Briefly, the formation of imine was indicated with the appearance of characteristic peaks corresponding to C=N stretching at 1640–1690 in the IR spectra of derivatives of 7-chloro-4-aminoquinoline–Schiff’s bases. ^1^H-NMR of all the desired derivatives (**HD1-23**) showed exact numbers of the desired protons. One representative derivative compound, **HD2**, is discussed here; all other derivative molecules showed a similar trend.

^1^H-NMR spectra exhibited a characteristic singlet peak for the hydrazide proton (N-H) and imine proton (C-H) at *δ* (ppm) 11.93 and 8.60. The total number of protons were 17, out of which 14 protons were in aromatic region *δ* (ppm) 7.18–8.53. These indicated the formation of (*E)-N*′-benzylidene-4-((7-chloroquinolin-4-yl)amino)benzohydrazide molecular hybrids. Eighteen peaks were observed in ^13^C-NMR spectra *δ* (ppm). Characteristic peaks of C=N of imine appeared at *δ* (ppm) 143.84. Due to the keto (C=O) group, peaks appeared at *δ* (ppm) 162.91, respectively. This further indicated the formation of the **HD1-23** compounds. The mass spectra of all the compounds (**HD1-23**) were found to be in agreement with the calculated values, consequently confirming, through HRMS, the formation of the desired hybrid compound.

**Scheme 2:**
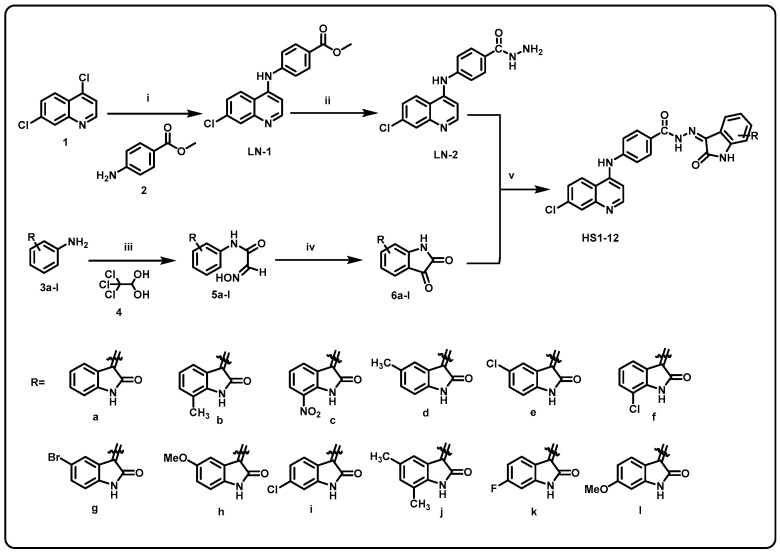
Reagents and conditions: (i) Methyl4-aminobenzoate, ethanol, p-TSA, 80 °C, 48 h. (ii) Hydrazine hydrate, ethanol, reflux at 80 °C, 24 h. (iii) NH_2_OH.HCl, chloral hydrate, HCl (2N), H_2_O, Na_2_SO_4_, 50 °C, 12 h. (iv) Conc. sulfuric acid, 80 °C, 4 h. (v) EtOH, 80 °C, 12 h. (a–l) Synthesized substituted isatin derivatives.

Synthesis of 4-aminoquinoline–isatin based molecular hybrids, connected through an aromatic linker (LN-2), was achieved using the approach as shown in Scheme 2. Briefly, commercially available 4,7-dichloroquinoline 1 was heated with methyl 4-aminobenzoate 2 at 80 °C for 48 h in presence of ethanol and p-Toluene sulfonic acid as a catalyst to give the linker LN-1 in quantitative yield. The LN-1 linker was further heated at 80 °C with hydrazine hydrate for 24 h in the presence of ethanol as a solvent to form LN-2 hydrazide as intermediates. An intermediate in a different series of reactions produced similar isonitrosoacetanilide (**5a**–**i**) by treating substituted anilines (**3a**–**i**) containing electron-donating and electron- withdrawing groups with hydroxylamine hydrochloride and chloral hydrate. Intermediate isonitrosoacetanilide derivatives (**5a**–**i**) were heated at 80 °C with concentrated H_2_SO_4_ to give the substituted isatins (**6a**–**i**). Finally, the linker LN-2 (4-((7-chloroquinolin-4-yl)amino)benzohydrazide) was reacted with substituted isatins (**6a**–**i**) in ethanol under reflux condition for 24 h to afford the corresponding 7-chloro-4-aminoquinoline–isatin-based molecular hybrids (**HS1-12**) in quantitative yield. Several spectroscopic methods, including FT-IR, ^1^H, ^13^C-NMR, and mass spectrometry, were used to characterize all intermediates and final aminoquinoline–isatin hybrids (**HS1-12**). The IR spectra of 7-chloro-4-aminoquinoline–isatin-based molecular hybrids showed the emergence of distinctive peaks related to -N*H*, -C=O, and -C=N extending at 3100–3500 cm^−1^, 1705–1725 cm^−1^, and 1640–1690 cm^−1^, respectively, indicating the confirmation of imine production. 

The ^1^H-NMR of all the desired hybrid molecules (**HS1-12**) showed the exact numbers of desired protons with corresponding peaks. One representative molecular hybrid **HS1** is discussed here; all other hybrids showed a similar trend. The ^1^H-NMR spectra of compound **HS1** was characterized under 400 MHz (*δ*, ppm) using DMSO-*d*_6_ as solvent. Compound **HS1** exhibited a total of 16 protons present in the compound. A broad singlet peak appeared at 13.94, indicating a characteristic peak for the -N*H* proton of hydrazide, and a sharp singlet peak appeared at 11.37, indicating the -N*H* proton of the isatin core. All 13 aromatic protons appeared in the range between 8.62 and 6.96. The -N*H* proton of the 4-aminoquinoline ring appeared at 9.50 as a singlet. Furthermore, 22 peaks were observed in the ^13^C-NMR (100.6 MHz, *δ,* ppm) spectra of compound **HS1**, which included a characteristic peak of -C=N of imine, which appeared at 134.76. A peak appeared at 163.58, which clearly indicates -C=O of isatin, respectively, indicating the formation of **HS1-12** hybrid molecules.

Before performing any biological study, the mass spectra of all compounds (**HS1-12**) were found to be in agreement with the calculated values and were consequently confirmed by HRMS, validating the formation of the desired molecules.

### 2.2. Pharmacological Evaluation

#### 2.2.1. Drug-Likeliness Assessment

In silico studies were conducted using the Schrodinger 2022-4 software to evaluate the drug-likeliness properties of 4-aminoquinoline hydrazones and isatin hybrids. Most of these compounds demonstrated favorable drug-like characteristics (Supplementary Table S1).

#### 2.2.2. In Vitro Screening 

All 4-aminoquinoline hydrazones and isatin hybrids, designated as (**HD1-23** and **HS1-12**), respectively, were subjected to in vitro screening to assess their inhibitory efficacy against selected bacterial strains. The antibacterial evaluation was conducted by measuring the zone of inhibition (ZOI) on Mueller–Hilton agar (MHA) plates against the tested standard isolates, i.e., *E. faecalis*, *B. subtilis*, *S. aureus*, and *P. aeruginosa* (Table 1). The screening commenced with an initial concentration of 10 mg/mL as the upper limit for the classification of potent compounds. Those exhibiting ineffective activity at this concentration were excluded from further analysis. The majority of the **HD** compounds exhibited measurable inhibitory zones against *E. faecalis* and *P. aeruginosa*. Conversely, these compounds displayed minimal significant antimicrobial activity against *B. subtilis*. Notably, compounds **HD1**, **HD4**, **HD6**, and **HD11** demonstrated good to moderate antibacterial activity across various bacterial strains. Within the **HS** series, most compounds demonstrated inhibitory effects against *E. faecalis* and *S. aureus*. However, the **HS** series did not exhibit any zones of clearance against *P. aeruginosa*. Among them, compounds **HS7** and **HS8** exhibited mild activity against the tested isolates. Ciprofloxacin was used as the standard reference drug in this study for benchmarking antimicrobial activity.

#### 2.2.3. Minimum Inhibitory Concentration (MIC)

Based on their zones of inhibition, the selected compounds **HD1**, **HD4**, **HD6**, **HD11**, **HS2**, **HS7**, and **HS8** were further assessed as possible antibacterials. In vitro screening of the compounds identified **HD6** and **HS8**, among others, as having superior antibacterial activity against the tested bacterial strains, as shown in (Table 2). **HD6** demonstrated good to moderate antibacterial potential, having MICs of 8 μg/mL against *B. subtilis*, 128 μg/mL against *E. faecalis* and *S. aureus*, and 16 μg/mL against *P. aeruginosa*, while **HS8** showed MICs of 256 μg/mL against the strains of *E. faecalis* and *B. subtilis*. In the case of **HS8**, no growth inhibition was observed against the strains *S. aureus* and *P. aeruginosa*, as the MIC values exceeded 500 μg/mL. This study concluded that **HD6** and **HS8** are selective inhibitors of these selective bacterial strains, warranting further investigation. 

#### 2.2.4. Minimum Bactericidal Concentration (MBC)

The minimum bactericidal concentration (MBC) indicates the lowest concentration at which an antimicrobial agent successfully eradicates microorganisms. To evaluate the antibacterial efficacy of a compound, the ratios of MBC to MIC are compared. If the MBC/MIC ratio is ≤2, the compound is classified as bactericidal. Conversely, a ratio between 2 and 16 suggests the compound is bacteriostatic. (Supplementary Data Table S2) provides the MBC values and the ratios of MIC to MBC, denoted as “R” for **HD** and **HS** compounds against the bacterial strains, with MBC indicating the concentration required to kill 99.9% of bacteria. Notably, the MIC/MBC ratios for *B. subtilis*, *S. aureus*, and *P. aeruginosa* strains were all 1, and it was found to be 0.5 for *E. faecalis*, which suggested that **HD6** has a potent bactericidal effect closely aligning its bacteriostatic properties. In contrast, all **HS** compounds exhibited MBC values greater than 2 for all bacterial strains, indicating a lack of bactericidal capabilities.

#### 2.2.5. Disc Diffusion Assay

The antibacterial effectiveness of the test compounds **HD6** and **HS8** were observed using the disc diffusion assay on Mueller–Hinton agar (MHA) plates at concentrations equivalent to their ½ MIC, MIC, and 2MIC values. The assay demonstrated that the zones of clearance were dose-dependent, varying with the different concentrations of the two compounds used (Table 3). Upon treatment with the compound **HD6**, increasing zones of inhibition were seen of 11 mm, 11 mm, and 12 mm for ½ MIC, MIC, and 2MIC, respectively, around the discs against Gram-positive *S. aureus*. For Gram-negative *P. aeruginosa*, the recorded ZOI results were 9 mm at (½ MIC), 10 mm at (MIC), and 11 mm at (2MIC), as shown in Table 3. **HS8** demonstrated mild activity against Gram-positive *E. faecalis* with a zone of 12 mm at 2MIC but displayed lower zones of inhibition against *B. subtilis* and *P. aeruginosa*.

#### 2.2.6. Combination Study 

Table 4 provides a comprehensive in vitro analysis on the synergistic antibacterial activity of compounds **HD6** and **HS8** in combination with the standard antibiotic ciprofloxacin (CIP) against four bacterial isolates. When evaluating the impact on *E. faecalis*, a many-fold decrease in the MIC values of both **HD6** and **HS8** was observed when used in combination with ciprofloxacin, indicating a synergistic effect against this strain. In addition, the results demonstrated that **HD6** also showed a significant increase in antibacterial effectiveness against the strains *E. faecalis* (FICI = 0.515), *B. subtilis* (FICI = 0.5), and *P. aeruginosa* (FICI = 0.375) in combination with CIP, showcasing a synergistic mode of interaction. Conversely, for *S. aureus*, the interaction between **HD6** and CIP appears to be indifferent. For **HS8** in combination with CIP against *B. subtilis* and *S. aureus*, the mode of interaction was indifferent, with FICI values seen to be (>0.5). These results suggest that the compound **HD6** exhibits a strong synergistic effect (i.e., a many-fold decrease in MIC values of the test compounds) with the standard drug CIP, which might be beneficial for treating resistant bacterial strains through combination therapy. The level of synergy between the examined compounds was assessed using the FICI (fractional inhibitory concentration index). Synergy was indicated by FICI values of 0.5 or less, while antagonism was shown by values of 4 or more. FICI values greater than 0.5 but less than 4 were regarded as indifferent.

#### 2.2.7. Hemolytic Assay

**Figure 2:**
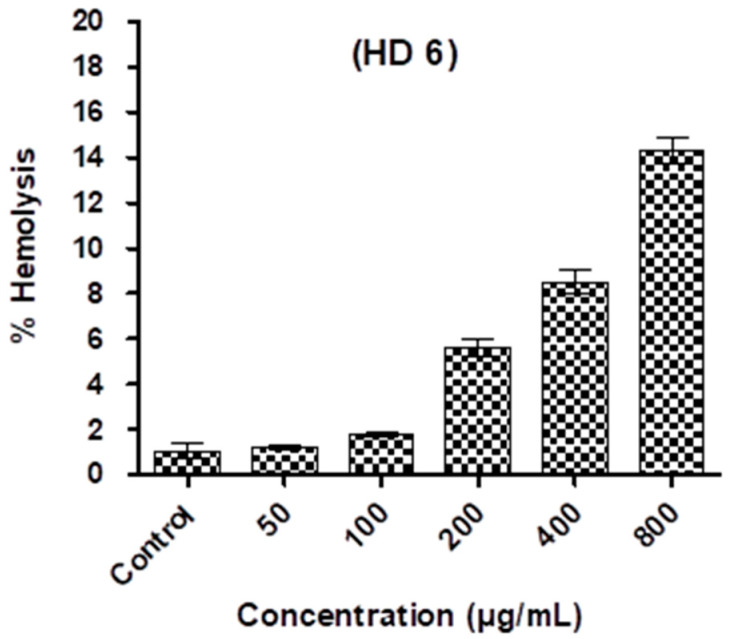
Hemolytic assay of compound **HD6** with standard drug ciprofloxacin on human red blood cells (hRBCs).

The in vitro toxicity of compound **HD6** was evaluated through the procedure of hemolytic assay to investigate its effects on human red blood cells (hRBCs) across a range of concentrations from 50 to 800 μg/mL, with ciprofloxacin as a control standard. Our findings show that at concentrations up to 100 μg/mL, **HD6** does not induce significant hemolysis, with less than 4% of RBCs undergoing lysis exhibiting non-toxicity. However, at a high concentration of 400 μg/mL, the lysis observed increased to 8% (Figure 2).

#### 2.2.8. Zone of Inhibition on Environmental Resistant Strains

Multidrug-resistant strains were taken from various environmental waterbodies for this study. A total of 16 MDR strains were subjected to antimicrobial testing against the **HD6** compound (Supplementary Materials Table S3). Ampicillin was included as a standard control reference to benchmark the efficacy of **HD6**. Remarkably, **HD6** exhibited a significant zone of inhibition specifically against the MDR strain designated AA 237, indicating its potent antimicrobial activity. This study suggested that **HD6** might be an effective candidate for combating multidrug-resistant bacterial infections. 

## 3. Molecular Docking and Simulation Studies 

### 3.1. Analysis of Molecular Docking and BFE Calculations

**Figure 3:**
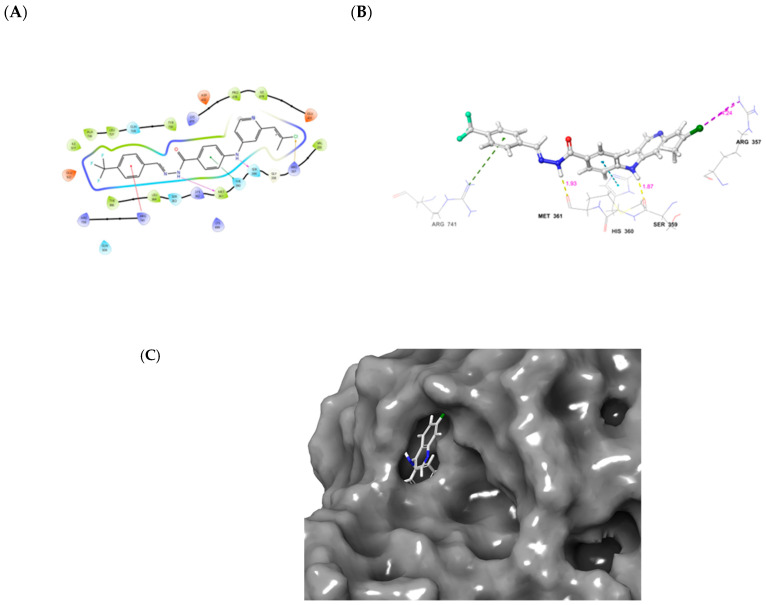
(**A**) Two-dimensional interaction diagram of the biofilm-associated protein with the ligand **HD6**. (**B**) Three-dimensional interaction diagram of the protein complex. (**C**) Surface representation of the protein–ligand complex.

The Glide module of Schrödinger suite 2022-4 was employed for extra precision docking (XP) within the predicted catalytic pocket. The biofilm-associated protein (7C7U) docked with **HD6** generated the docking score and binding free energy and showed the interactions. It showed the XP docking score to be −2.707 Kcal/mol, and binding free energy was calculated to be −60.74 Kcal/mol. The 2D interaction diagram generated by Maestro illustrates the formation of hydrogen-bond interactions with residues SER_359 and MET_361, with a bond distance of 1.87 Å and 1.93 Å, respectively; pi–pi interaction with HIS_360 and ARG_741; and ionic interaction with ARG_357 with a bond distance of 3.49 Å (Figure 3).

### 3.2. Molecular Dynamic Simulation

**Figure 4:**
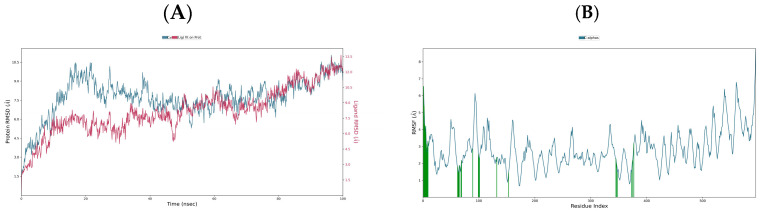
(**A**) RMSD of the protein–ligand complex. (**B**) RMSF of the protein–ligand complex. Green line in the figure represents the specific amino acid residues which are taking part in the interaction with the ligand.

To decode the dynamic behavior of the protein–ligand complex at the atomic level, molecular dynamic simulations were performed. To assess the stability of the protein and the chosen ligands, we conducted a 100 ns MD simulation of the protein with the ligand. Some of the properties were calculated during simulation, such as the root mean squared deviation (RMSD), RMSF, and some of the ligand properties mentioned below. The RMSD of the protein complex was calculated from 0 to 100 ns. From 0 to 40 ns, RMSD is from 2.00 Å to 10.48 Å. From 40 to 80 ns, RMSD is from 5.45 ns to 8.59 ns. Then from 80 ns to 100 ns, RMSD is from 7.19 ns to 11.05 ns. The average value throughout the simulation period of 100 ns exhibited between this range only (above 2 Å and less than 10 Å) (Figure 4). This indicates that the protein forms an average stable complex in the presence of the ligand and does not show much deviation. 

The protein complex’s root mean square fluctuation (RMSF) assesses individual residues’ dynamic behavior during the entire simulation period of 100 ns. LYS_415 and LEU_416 showed an RMSF value of 1.82 Å and 1.52 Å, respectively. The residues exhibit flexible and dynamic behavior according to their RMSF values. A high deviating RMSF protein value means that throughout the simulation time, the particular area of the protein is very flexible. The atom in that specific region is flexible and moves away from its original position. A low deviation in RMSF value indicates that the flexible behavior of that residue is low, which means that residues involved in interaction with the ligand are held stable. It occupies its original position and shows less fluctuation, indicating a relatively more stable complex [11]. The RMSD of the ligand was measured to be between 1.1 Å and 1.5 Å. Throughout the simulation period of 100 ns, the ligand showed a value between this range and not beyond, hence indicating the stability of the ligand.

To evaluate the stability and compactness of the ligand with the protein, we analyzed the radius of gyration (rGyr). The rGyr tells us how close the ligand is fitted with the protein [13]. The values of the complex range from 6.71 to 6.75 Å throughout 100 ns. It indicated a close fitness of the ligand with the protein (rigid complex) during the entire simulation time. A low Rg indicates a compact rigid structure, while a high Rg indicates a loose structure [49].

**Figure 5:**
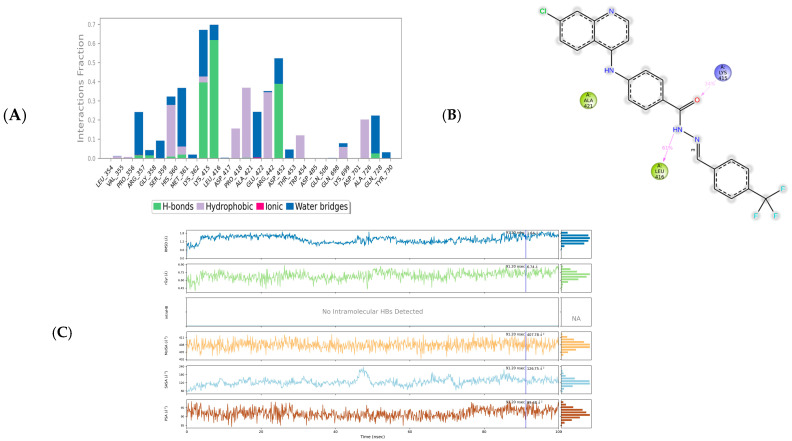
(**A**) Protein interaction/(contact) with ligand **HD6**. (**B**) A detailed schematic of the ligand. (**C**) Ligand properties such as ligand RMSD, radius of gyration, intermolecular H-bonds, molecular surface area, solvent-accessible surface area, and polar surface area.

Solvent-accessible surface area (SASA) is a key property of the ligand, which indicates solvent exposure to the ligand surface. It is the feature that regulates the protein stability and folding dynamics by changing the protein’s conformation in the presence of the ligand. SASA measures the compactness of the complex system. A lower SASA value indicates that ligand atoms are stabilizing the protein’s conformation by exposing aqueous solvent to the protein chain and facilitating the formation of a compact structure, and a SASA value with a higher deviation means low stability of the protein in the presence of the ligand, which exposes hydrophobic residues with aqueous solvent and destabilizes the structure of the protein [50]. Hence, the SASA value indicates the folding mechanism of the protein [14]. The value of the ligand during the entire simulation time lies between 100 Å and 120 Å. During the entire period, the value lies in this range only (Figure 5).

### 3.3. Molecular Dynamics-Based Investigation of Interactions

The interactions between the **HD6** ligand and the protein are analyzed throughout the simulation time of 0–100 nsec. Residues VAL_355, PRO_356, ARG_357, GLY_358, SER_359, HIS_360, MET_361, LYS_362, LYS_415, LEU_416, ASP_417, PRO_418, ALA_421, GLU_422, ARG_442, ASP_452, THR_453, TRP_454, ASP_485, GLN_506, GLN_698, LYS_699, ASP_701, ALA_726, GLN_728, and TYR_730 show different numbers of interactions, like hydrogen bond, hydrophobic interaction, ionic interactions, and water bridges. Residues LYS_415 and LEU_416 show interactions for more than 30% of the simulation period, i.e., 100 nsec. 

## 4. Discussion

Antimicrobial resistance (AMR) poses global challenges in the discovery of novel drugs. To overcome these challenges, alternative therapeutic strategies are urgently required by exploring novel antibacterial agents. The present study attempted to explore the potential of new 4-aminoquinoline-hydrazones and isatin-based hybrid compounds as antimicrobial agents. Heterocyclic compounds, synthesized by the fusion of two or more active pharmacophores, have been critical towards the development of antimicrobial treatments. 4-aminoquinoline-based heterocycles are well-known in medicinal chemistry for their antibacterial, anti-inflammatory, analgesic, antimalarial, anticancer, antiviral, antileishmanial, and antitubercular effects. Prompted by this, 4-aminoquinoline-benzohydrazide-based molecular hybrids bearing aryl aldehydes (**HD1-23**) and substituted isatin warheads (**HS1-12**) having drug-like properties were developed to address the issue of drug resistance. The synthesized compounds demonstrated considerable antibacterial properties, with **HD6** emerging as the most potent candidate. Notably, among the 35 synthesized derivatives, **HD6** exhibited an MIC of 8 μg/mL against *Bacillus subtilis* and significant ZOI values (up to 11 mm) against both *S. aureus* and *E. faecalis*. Furthermore, the combination of **HD6** with ciprofloxacin resulted in a marked reduction in MIC values, demonstrating a synergistic interaction, particularly against *P. aeruginosa* (fractional inhibitory concentration index, FICI = 0.37). Clinically, a FICI value of 0.37 signifies strong synergy, indicating that the combination effectively reduces bacterial resistance, allowing for lower doses of each drug to achieve the desired antimicrobial effect. This synergistic interaction can lead to fewer side effects, reduced risk of toxicity, and improved patient outcomes. This synergy underscores the potential of **HD6** in combination therapy, enhancing its effectiveness against resistant infections. For example, the MIC of **HD6** against *E. faecalis* decreased dramatically from 128 μg/mL to 2 μg/mL in the presence of ciprofloxacin, supporting its role in developing more effective treatment strategies for combating antimicrobial resistance. The hemolytic activity of **HD6** was also evaluated using human red blood cells (hRBCs), revealing a favorable safety profile with less than 8% toxicity at concentrations up to 400 μg/mL. This low level of toxicity suggests that **HD6** could be a viable candidate for further development as an antimicrobial agent. Importantly, **HD6′**s activity against the MDR strain AA 237 underlines its promise as a candidate for new therapeutic strategies in combating multidrug-resistant infections. We conducted ZOI on other MDR strains (MDR strains), but no significant results were obtained (Supplementary Materials Table S4). These data from the in vitro studies prompted us to explore the possible mode of action. Molecular docking studies highlighted the strong binding affinity of **HD6** with biofilm-associated protein (PDB ID: 7C7U), providing pivotal insights into its potential mechanism of action. The subsequent molecular dynamics (MD) simulations allowed for the evaluation of the stability and dynamic behavior of the ligand–protein complexes under physiological conditions, simulating the interactions that could occur within a bacterial environment. The findings of this study align with the previous literature that emphasizes the efficacy of quinoline derivatives against various bacterial strains, further supporting the therapeutic potential of 4-aminoquinoline-benzohydrazide-based molecular hybrids in addressing antibiotic resistance. This research contributes to ongoing efforts in combating resistance management and reducing the burden of microbial infections worldwide. Future research should aim to elucidate the specific mechanisms of action of the most effective compounds, conduct in vivo studies to assess both efficacy and safety, and explore the structure–activity relationships (SAR) to optimize the pharmacological properties of these hybrids. Additionally, expanding the range of bacterial strains tested and investigating potential synergistic interactions with other antibiotic classes could facilitate the development of robust treatment strategies against AMR.

## 5. Materials and Methods

### 5.1. Chemistry

All chemicals (reagents and solvents) for synthesis were purchased at ≥95% purity from Sigma-Aldrich (St. Louis, MO, USA), TCI (Zwijndrecht, Belgium), Avra (Hyderabad, India), Merck (Darmstadt, Germany), Fisher scientific, SRL (Mumbai, India), and GLR Innovation (New Delhi, India) and used without further purification. The reaction was confirmed by analytical silica gel TLC (thin-layer chromatography) plates pre-coated on aluminum sheets (DC Kieselgel 60 F_254_, Merck KGaA, Darmstadt, Germany) used under 254 nm (shortwave) UV light. Iodine (I_2_) vapor staining was used for visualization of spots. All compounds were purified by flash column chromatography with Combi Flash NextGen 100 (Teledyne ISCO, Lincoln, NE, USA) using a 230–400 mesh silica gel purchased from Sisco Research Laboratories (Mumbai, India). Infrared spectra were recorded, and only major peaks were reported in cm^−1^ using an Agilent Cary 630FT-IR spectrometer. A digital melting point instrument (M-560, Buchi, Switzerland) was used for the measurement of melting points, which were uncorrected. DMSO-*d*_6_ (as a solvent) with Tetramethylsilane (TMS) as an internal standard on a Bruker Avance Neo 400 MHz spectrometer at 400 MHz and 100.6 MHz, and (**HD1-23**) and (**HS1-12**) were used for ^1^H and ^13^C-NMR spectra, respectively. s (singlet), d (doublet), t (triplet), and m (multiplet) were labelled as the splitting pattern. The chemical shift *(δ)* values in ppm were referenced to the residual solvent, signals of DMSO-*d*_6_; ^1^H *δ* = 2.50 ppm, ^13^C *δ* = 39.5 ppm, and unit of coupling constants (*J*) is Hertz (Hz). An Agilent G6530AA (LC-HRMS-Q-TOF) spectrometer was used for recording of the mass spectra. Procedure of synthesis and spectral characterization is discussed below.

#### 5.1.1. General Procedure for the Synthesis of Methyl 4-((7-Chloroquinolin-4-yl)amino)benzoate (LN-1)

A solution of 4,7-dichloroquiline 1 (1.0 equiv.) and methyl-4-aminobenzoate 2 (1.0 equiv.) in absolute ethanol was refluxed at 80 °C in the presence of *p*-TSA (0.2 equiv.) as catalyst for 48 h. During this process, the formation of precipitate takes place, and reaction was monitored by TLC using hexane/ethylacetate (1:1). Then the reaction mixture was cooled down to room temperature. The resultant precipitate was directly filtered without prior addition of ice-cold water and washed with water to obtain the desired product LN-1 (91% yield) [51]. 

#### 5.1.2. General Procedure of 4-((7-Chloroquinolin-4-yl)amino)benzohydrazide (LN-2)

In a dried round-bottom flask, compound LN-1 (1.0 equiv.) was dissolved in absolute ethanol followed by the addition of hydrazine hydrate (20 equiv.) dropwise. The reaction mixture was allowed to reflux at 80–90 °C overnight. A white solid precipitate was formed, and the reaction was monitored by TLC. The reaction mixture was cooled down to room temperature followed by the addition of ice-cold water, resulting in the formation of precipitate. The precipitate was filtered and washed with water to obtain the desired product LN-2 in a good to excellent yield [52,53]. 

#### 5.1.3. General Procedure of Compounds (**HD1-23**)

In an RB flask, the hydrazide linker LN-2 (1.0 eq) was dissolved in absolute ethanol followed by the addition of appropriate aldehydes (**3a**–**w**) (1.0 eq). Then a few drops of glacial acetic acid were added to the reaction mixture and reaction was allowed to be refluxed at 80–90 °C for 8 h. Reaction progress was monitored by TLC. After completion of the reaction, the reaction mixture was cooled to room temperature, and ice-cold water was added, resulting in the formation of precipitate. The precipitate was filtered and washed with water to obtain the desired product (**HD1-23**) in a good to excellent yield [54]. 

(a) (*E*)-4-((7-chloroquinolin-4-yl)amino)-*N*′-(2-hydroxybenzylidene)benzohydrazide (**HD1**).


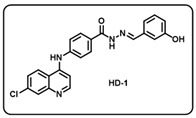


Creamy white solid; yield 83%; R*_f_* = 0.40 (methanol/dichloromethane = 5:95); mp: 272 °C; FT-IR (cm^−1^): 3495, 3231, 2826, 2082, 1941, 1694, 1521, 1361, 1284, 1171, 1078; ^1^H NMR (400 MHz, DMSO-*d*_6_) (*δ*, ppm): 12.15 (s, 1H, N-*H_hydrazide_*), 11.40 (s, 1H, O-*H*), 9.62 (s, 1H, N-*H*), 8.68 (s, 1H, Ar-*H*), 8.62 (s, 1H, C-*H*_imine_), 8.48 (d, *J* = 9.06 Hz, 1H, Ar-*H*), 8.04 (d, *J* = 8.28 Hz, 2H, Ar-*H*), 7.98 (s, 1H, Ar-*H*), 7.66 (d, *J* = 8.96 Hz, 1H, Ar-*H*), 7.54 (t, *J* = 7.77 Hz, 3H, Ar-*H*), 7.31 (t, *J* = 7.77 Hz, 1H, Ar-*H*), 7.22 (d, *J* = 5.18 Hz, 1H, Ar-*H*), 6.97–6.92 (m, 1H, Ar-*H*); ^13^C NMR (100.6 MHz, DMSO-*d*_6_) (*δ*, ppm): 172.51, 162.63, 157.94, 151.67, 148.44, 147.74, 144.51, 135.06, 131.75, 130.02, 129.71, 127.47, 127.39, 126.10, 125.27, 120.84, 119.79, 119.18, 116.89; HRMS (*m*/*z*) calcd. for C_23_H_17_ClN_4_O_2_: 416.1040. Found: 417.1117 [M + H]^+^. 

(b) (*E*)-*N*′-benzylidene-4-((7-chloroquinolin-4-yl)amino)benzohydrazide (**HD2**).


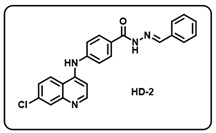


Light yellow solid; yield 85%; R*_f_* = 0.45 (methanol/dichloromethane = 5:95); mp: 254.6 °C; FT-IR (cm^−1^): 3402, 3201, 2802, 2105, 1816, 1574, 1446, 1358, 1269, 1184, 1135; ^1^H NMR (400 MHz, DMSO-*d*_6_) (*δ*, ppm): 11.93 (s, 1H, N-*H_hydrazide_*), 8.60 (s, 1H, C-*H*_imine_), 8.53 (d, *J* = 8.07 Hz, 2H, Ar-*H*), 8.04 (d, *J* = 8.05 Hz, 2H, Ar-*H*), 8.00 (d, *J* = 0.92 Hz, 1H, Ar-*H*), 7.75 (d, *J* = 5.91 Hz, 2H, Ar-*H*), 7.68 (d, *J* = 8.79 Hz, 1H, Ar-*H*), 7.55–7.46 (m, 5H, Ar-*H*), 7.18 (d, *J* = 5.26 Hz, 1H, Ar-*H*); ^13^C NMR (100.6 MHz, DMSO-*d*_6_) (*δ*, ppm): 162.91, 150.78, 148.66, 147.95, 143.84, 135.52, 134.87, 130.47, 129.70, 129.31, 128.65, 127.51, 126.45, 126.29, 125.44, 121.39, 118.92, 103.76; HRMS (*m*/*z*) calcd. for C_23_H_17_ClN_4_O: 400.1091. Found: 401.1162 [M + H]^+^.

(c) (*E*)-*N*′-(2-chlorobenzylidene)-4-((7-chloroquinolin-4-yl)amino)benzohydrazide (**HD3**).


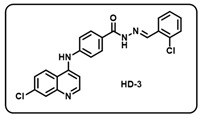


Light yellow solid; yield 86%; R*_f_* = 0.59 (methanol/dichloromethane = 5:95); mp: 278.5 °C; FT-IR (cm^−1^): 3337, 3179, 2881, 2923, 1824, 1651, 1565, 1523, 1432, 1373, 1239; ^1^H NMR (400 MHz, DMSO-*d*_6_) (*δ*, ppm): 12.10 (s, 1H, N-*H_hydrazide_*), 9.62 (s, 1H, N-*H*), 8.91 (s, 1H, C-*H*_imine_), 8.61 (d, *J* = 2.98 Hz, 1H, Ar-*H*), 8.48 (d, *J* = 9.03 Hz, 1H, Ar-*H*), 8.03 (d, *J* = 7.96 Hz, 3H, Ar-*H*), 7.98 (d, *J* = 1.44 Hz, 1H, Ar-*H*), 7.66 (dd, *J* = 9.02, 1.72 Hz, 1H, Ar-*H*), 7.52 (d, *J* = 8.38 Hz, 3H, Ar-*H*), 7.45 (t, *J* = 3.18 Hz, 2H, Ar-*H*), 7.21 (d, *J* = 5.35 Hz, 1H, Ar-*H*); ^13^C NMR (100.6 MHz, DMSO-*d*_6_) (*δ*, ppm): 172.49, 162.96, 151.63, 148.99, 147.80, 144.42, 143.70, 135.07, 133.60, 132.14, 131.85, 130.39, 129.47, 128.07, 127.86, 127.29, 126.10, 120.83, 119.21, 104.18; HRMS (*m*/*z*) calcd. for C_23_H_16_Cl_2_N_4_O: 434.0701. Found: 435.0776 [M + H]^+^.

(d) (*E*)-*N*′-(4-chlorobenzylidene)-4-((7-chloroquinolin-4-yl)amino)benzohydrazide (**HD4**).


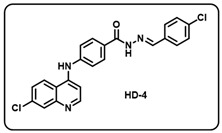


Creamy yellow solid; yield 81%; R*_f_* = 0.59 (methanol/dichloromethane = 5:95); mp: 252.5 °C; FT-IR (cm^−1^): 3412, 3024, 2105, 1922, 1579, 1524, 1446, 1362, 1287, 1086, 1064; ^1^H NMR (400 MHz, DMSO-*d*_6_) (*δ*, ppm): 11.96 (s, 1H, N-*H_hydrazide_*), 9.65 (s, 1H, N-*H*), 8.60 (d, *J* = 4.50 Hz, 1H, C-*H*_imine_), 8.49 (t, *J* = 4.11 Hz, 2H, Ar-*H*), 8.03–7.98 (m, 3H, Ar-*H*), 7.77 (d, *J* = 7.62 Hz, 2H, Ar-*H*), 7.66 (d, *J* = 8.92 Hz, 1H, Ar-*H*), 7.52 (d, *J* = 8.39 Hz, 4H, Ar-*H*), 7.20 (d, *J* = 5.26 Hz, 1H, Ar-*H*); ^13^C NMR (100.6 MHz, DMSO-*d*_6_) (*δ*, ppm): 172.51, 162.99, 151.36, 148.11, 146.33, 144.16, 135.23, 134.88, 133.28, 131.60, 129.70, 129.39, 129.12, 127.01, 126.16, 125.30, 121.03, 119.10, 104.00; HRMS (*m*/*z*) calcd. for C_23_H_16_Cl_2_N_4_O: 434.0701. Found: 435.0778 [M + H]^+^.

(e) (*E*)-4-((7-chloroquinolin-4-yl)amino)-*N*-(4-fluorobenzylidene)benzohydrazide (**HD-5**).


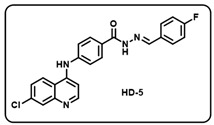


Light yellow solid; yield 84%; R*_f_* = 0.37 (methanol/dichloromethane = 5:95); mp: 258.3 °C; FT-IR (cm^−1^): 3411, 3213, 2812, 2343, 1918, 1650, 1566, 1507, 1444, 1367, 1299; ^1^H NMR (400 MHz, DMSO-*d*_6_) (*δ*, ppm): 11.90 (s, 1H, N-*H_hydrazide_*), 9.62 (s, 1H, N-*H*), 8.60 (d, *J* = 5.27 Hz, 1H, C-*H*_imine_), 8.50 (d, *J* = 9.33 Hz, 2H, Ar-*H*), 8.02 (d, *J* = 8.43 Hz, 2H, Ar-*H*), 7.98 (d, *J* = 1.43 Hz, 1H, Ar-*H*), 7.80 (s, 2H, Ar-*H*), 7.66 (d, *J* = 8.94 Hz, 1H, Ar-*H*), 7.52 (d, *J* = 8.43 Hz, 2H, Ar-*H*), 7.31 (t, *J* = 8.43 Hz, 2H, Ar-*H*), 7.20 (d, *J* = 5.44 Hz, 1H, Ar-*H*); ^13^C NMR (100.6 MHz, DMSO-*d*_6_) (*δ*, ppm): 172.49, 162.93, 162.29, 151.51, 148.82, 147.98, 146.72, 144.19, 135.13, 131.51, 129.67, 128.23, 127.20, 126.10, 125.29, 120.98, 119.14, 116.47, 116.26, 104.01; HRMS (*m*/*z*) calcd. for C_23_H_16_ClFN_4_O: 418.0997. Found: 419.1070 [M + H]^+^.

(f) (*E*)-4-((7-chloroquinolin-4-yl)amino)-*N*′-(4-(trifluoromethyl)benzylidene)benzo- hydrazide (**HD6**).


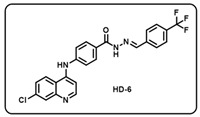


Off-white solid; yield 87%; R_f_ = 0.3.9 (methanol/dichloromethane = 5:95); mp: 112.5 °C; FT-IR (cm^−1^): 3189, 3036, 2344, 2108, 1564, 1527, 1445, 1371, 1321, 1168, 1106; ^1^H NMR (400 MHz, DMSO-*d*_6_) (*δ*, ppm): 12.06 (s, 1H, N-*H_hydrazide_*), 9.76 (s, 1H, N-*H*), 8.61 (d, *J* = 4.95 Hz, 1H, Ar-*H*), 8.56 (s, 1H, C-*H*_imine_), 8.46 (d, *J* = 8.98 Hz, 1H, Ar-*H*), 8.15 (d, *J* = 8.11 Hz, 1H, Ar-*H*), 8.02–7.95 (m, 3H, Ar-*H*), 7.89 (t, *J* = 5.25 Hz, 2H, Ar-*H*), 7.84 (d, *J* = 7.76 Hz, 1H, Ar-*H*), 7.66–7.62 (m, 1H, Ar-*H*), 7.51 (d, *J* = 8.23 Hz, 1H, Ar-*H*), 7.44 (d, *J* = 8.50 Hz, 1H, Ar-*H*), 7.23 (d, *J* = 4.96 Hz, 1H, Ar-H); ^13^C NMR (100.6 MHz, DMSO-*d*_6_) (*δ*, ppm): 166.72, 152.16, 149.67, 147.30, 138.89, 135.19, 134.78, 130.56, 129.77, 128.84, 128.04, 127.86, 127.69, 126.20, 126.06, 125.95, 125.18, 120.97, 120.53, 119.40, 104.38; HRMS (*m*/*z*) calcd. for C_24_H_16_ClF_3_N_4_O: 468.0965. Found: 469.1045 [M + H]^+^.

(g) (*E*)-4-((7-chloroquinolin-4-yl)amino)-*N*′-(2-nitrobenzylidene)benzohydrazide (**HD7**).


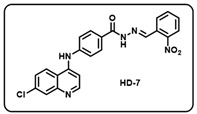


Light yellow solid; yield 79%; R*_f_* = 0.43 (methanol/dichloromethane = 5:95); mp: 285.8 °C; FT-IR (cm^−1^): 3342, 3178, 2331, 1167, 1565, 1519, 1460, 1256, 1190, 1130; ^1^H NMR (400 MHz, DMSO-*d*_6_) (*δ*, ppm): 12.19 (s, 1H, N-*H_hydrazide_*), 9.44 (s, 1H, N-*H*), 8.90 (s, 1H, C-*H*_imine_), 8.61 (d, *J* = 4.86 Hz, 1H, Ar-*H*), 8.45 (d, *J* = 8.99 Hz, 1H, Ar-*H*), 8.17 (d, *J* = 8.11 Hz, 1H, Ar-*H*), 8.10 (d, *J* = 7.98 Hz, 1H, Ar-*H*), 8.03 (d, *J* = 8.09 Hz, 1H, Ar-*H*), 7.97 (d, *J* = 1.94 Hz, 1H, Ar-*H*), 7.83 (t, *J* = 7.60 Hz, 1H, Ar-*H*), 7.68 (t, *J* = 7.93 Hz, 1H, Ar-*H*), 7.64 (dd, *J* = 9.0, 2.15 Hz, 1H, Ar-*H*), 7.52 (d, *J* = 8.73 Hz, 2H, Ar-*H*), 7.24 (d, *J* = 5.35 Hz, 1H, Ar-*H*); ^13^C NMR (100.6 MHz, DMSO-*d*_6_) (*δ*, ppm): 163.10, 159.14, 152.31, 149.87, 148.67, 147.12, 144.83, 142.80, 134.70, 134.16, 131.02, 129.80, 129.28, 128.14, 128.03, 127.31, 125.92, 125.14, 120.38, 119.45, 104.49; HRMS (*m*/*z*) calcd. for C_23_H_16_ClN_5_O_3_: 445.0942. Found: 446.1019 [M + H]^+^.

(h) (*E*)-4-((7-chloroquinolin-4-yl)amino)-*N*′-(3-nitrobenzylidene)benzohydrazide (**HD-8**).


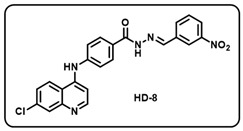


Greenish-yellow solid; yield 82%; R*_f_* = 0.29 (methanol/dichloromethane = 5:95); mp: 276.6 °C; FT-IR (cm^−1^): 3276, 3081, 2874, 2816, 2109, 1665, 1572, 1516, 1462, 1342, 1305; ^1^H NMR (400 MHz, DMSO-*d*_6_) (*δ*, ppm): 12.12 (s, 1H, N-*H_hydrazide_*), 9.54 (s, 1H, N-*H*), 8.61 (d, *J* = 4.91 Hz, 2H, Ar-*H*), 8.55 (s, 1H, C-*H*_imine_), 8.47 (d, *J* = 9.07 Hz, 1H, Ar-*H*), 8.27 (d, *J* = 7.58 Hz, 1H, Ar-*H*), 8.16 (d, *J* = 6.93 Hz, 1H, Ar-H), 8.03 (d, *J* = 7.98 Hz, 2H, Ar-*H*), 7.97 (d, *J* = 1.76 Hz, 1H, Ar-*H*), 7.75 (t, *J* = 7.94 Hz, 1H, Ar-*H*), 7.66 (dd, *J* = 8.92, 1.64 Hz, 1H, Ar-*H*), 7.53 (d, *J* = 7.39 Hz, 2H, Ar-*H*), 7.22 (d, *J* = 5.29 Hz, 1H, Ar-*H*); ^13^C NMR (100.6 MHz, DMSO-*d*_6_) (*δ*, ppm): 163.15, 151.86, 149.30, 148.68, 147.55, 145.30, 144.59, 136.78, 134.93, 133.80, 130.90, 129.79, 127.58, 126.01, 125.22, 124.58, 121.25, 120.66, 119.29, 104.27; HRMS (*m*/*z*) calcd. for C_23_H_16_ClN_5_O_3_: 445.0942. Found: 446.1019 [M + H]^+^.

(i) (*E*)-4-((7-chloroquinolin-4-yl)amino)-*N*′-(4-nitrobenzylidene)benzohydrazide (**HD9**).


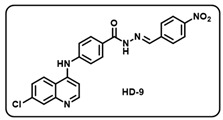


Deep yellow solid; yield 86%; R*_f_* = 0.43 (methanol/dichloromethane = 5:95); mp: 298.8 °C; FT-IR (cm^−1^): 3406, 3241, 3037, 2323, 2115, 1923, 1676, 1530, 1448, 1254, 1180; ^1^H NMR (400 MHz, DMSO-*d*_6_) (*δ*, ppm): 12.13 (s, 1H, N-*H_hydrazide_*), 9.51 (s, 1H, N-*H*), 8.57 (s, 2H, Ar-*H*), 8.45 (d, *J* = 8.14 Hz, 1H, C-*H*_imine_), 8.32 (d, *J* = 7.76 Hz, 2H, Ar-*H*), 8.00 (s, 5H, Ar-*H*), 7.66 (d, *J* = 8.48 Hz, 1H, Ar-*H*), 7.52 (d, *J* = 7.49 Hz, 2H, Ar-*H*), 7.22 (s, 1H, Ar-*H*); ^13^C NMR (100.6 MHz, DMSO-*d*_6_) (*δ*, ppm): 163.42, 158.92, 156.67, 151.98, 148.23, 147.43, 145.22, 14.24, 139.66, 134.89, 129.82, 128.38, 127.71, 126.04, 125.20, 124.56, 120.58, 104.99; HRMS (*m*/*z*) calcd. for C_23_H_16_ClN_5_O_3_: 445.0942. Found: 446.1019 [M + H]^+^.

(j) (*E*)-4-((7-chloroquinolin-4-yl)amino)-*N*′-(4-methoxybenzylidene)benzohydrazide (**HD10**).


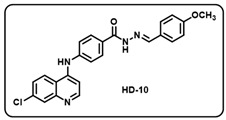


Creamy yellow solid; yield 80%; R*_f_* = 0.29 (methanol/dichloromethane = 5:95); mp: 253.1 °C; FT-IR (cm^−1^): 3259, 2964, 2837, 2323, 2105, 1648, 1571, 1509, 1452, 1366, 1251, 1063; ^1^H NMR (400 MHz, DMSO-*d*_6_) (*δ*, ppm): 11.75 (s, 1H, N-*H_hydrazide_*), 9.61 (s, 1H, N-*H*), 8.60 (d, *J* = 5.28 Hz, 1H, Ar-*H*), 8.48 (d, *J* = 8.13 Hz, 1H, Ar-*H*), 8.44 (s, 1H, C-*H*_imine_), 8.00 (d, *J* = 8.42 Hz, 2H, Ar-*H*), 7.98 (d, *J* = 2.01 Hz, 1H, Ar-*H*), 7.70–7.64 (m, 3H, Ar-*H*), 7.51 (d, *J* = 8.52 Hz, 2H, Ar-*H*), 7.19 (d, *J* = 5.51 Hz, 1H, Ar-*H*), 7.03 (d, *J* = 8.60 Hz, 2H, Ar-*H*), 3.82 (s, 3H, OC*H*_3_); ^13^C NMR (100.6 MHz, DMSO-*d*_6_) (*δ*, ppm): 162.77, 161.24, 151.60, 148.94, 147.95, 147.89, 144.07, 135.08, 129.59, 129.10, 128.46, 127.44, 127.30, 126.07, 125.27, 121.00, 119.15, 114.80, 103.96, 55.76; HRMS (*m*/*z*) calcd. for C_24_H_19_ClN_4_O_2_: 430.1197. Found: 431.1276 [M + H]^+^.

(k) (*E*)-4-((7-chloroquinolin-4-yl)amino)-*N*′-(3,4-dimethoxybenzylidene)benzo- hydrazide (**HD11**).


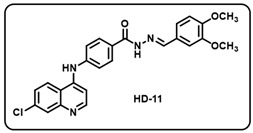


Creamy yellow solid; yield 84%; R*_f_* = 0.16 (methanol/dichloromethane = 5:95); mp: 178.4 °C; FT-IR (cm^−1^): 3394, 3250, 3047, 1919, 1576, 1508, 1412, 1324, 1269, 1100, 1018; ^1^H NMR (400 MHz, DMSO-*d*_6_) (*δ*, ppm): 11.81 (s, 1H, N-*H_hydrazide_*), 9.91 (s, 1H, N-*H*), 8.60–8.54 (m, 2H, Ar-*H*), 8.44 (s, 1H, C-*H*_imine_), 8.02 (t, *J* = 8.27 Hz, 3H, Ar-*H*), 7.70 (d, *J* = 8.71 Hz, 1H, Ar-*H*), 7.54 (d, *J* = 8.04 Hz, 2H, Ar-*H*), 7.36 (s, 1H, Ar-*H*), 7.21 (d, *J* = 7.84 Hz, 1H, Ar-*H*), 7.15 (d, *J* = 5.55 Hz, 1H, Ar-*H*), 7.03 (d, *J* = 7.98 Hz, 1H, Ar-*H*), 3.83 (d, *J* = 4.79 Hz, 6H, 2OC*H*_3_); ^13^C NMR (100.6 MHz, DMSO-*d*_6_) (*δ*, ppm): 162.75, 151.17, 150.26, 149.53, 149.23, 148.24, 147.23, 143.44, 135.80, 129.64, 129.17, 127.57, 126.43, 125.91, 125.53, 122.32, 121.76, 110.70, 111.94, 108.65, 103.48, 56.03, 55.91; HRMS (*m*/*z*) calcd. for C_25_H_21_ClN_4_O_3_: 460.1302. Found: 461.1382 [M + H]^+^.

(l) (*E*)-*N*′-(4-bromobenzylidene)-4-((7-chloroquinolin-4-yl)amino)benzohydrazide (**HD12**).


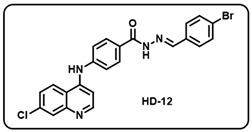


Greenish-yellow solid; yield 79%; R*_f_* = 0.40 (methanol/dichloromethane = 5:95); mp: 268.1 °C; FT-IR (cm^−1^): 3391, 3244, 3018, 2342, 2069, 1917, 1646, 1562, 1523, 1446, 1361, 1032; ^1^H NMR (400 MHz, DMSO-*d*_6_) (*δ*, ppm): 11.92 (s, 1H, N-*H_hydrazide_*), 9.46 (s, 1H, N-*H*), 8.60 (d, *J* = 5.16 Hz, 1H, Ar-*H*), 8.46 (s, 1H, C-*H*_imine_), 8.43 (s, 1H, Ar-*H*), 8.00–7.96 (m, 3H, Ar-*H*), 7.73–7.62 (m, 5H, Ar-*H*), 7.50 (d, *J* = 8.31 Hz, 2H, Ar-*H*), 7.21 (d, *J* = 5.18 Hz, 1H, Ar-*H*); ^13^C NMR (100.6 MHz, DMSO-*d*_6_) (*δ*, ppm): 172.49, 162.97, 152.18, 149.70, 147.32, 146.51, 134.77, 134.19, 132.31, 130.66, 129.67, 129.34, 129.89, 125.94, 125.17, 123.64, 120.57, 119.38, 104.32; HRMS (*m*/*z*) calcd. For C_23_H_16_BrClN_4_O: 478.0196. Found: 481.0258 [M + H]^+^.

(m) (*E*)-4-((7-chloroquinolin-4-yl)amino)-*N*′-(4-hydroxybenzylidene)benzohydrazide (**HD13**).


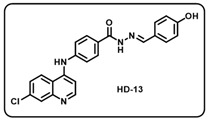


Light yellow solid; yield 85%; R*_f_* = 0.18 (methanol/dichloromethane = 5:95); mp: 299.8 °C; FT-IR (cm^−1^): 3166, 2998, 2348, 2105, 1947, 1605, 1506, 1444, 1288, 1240, 1165; ^1^H NMR (400 MHz, DMSO-*d*_6_) (*δ*, ppm): 11.69 (s, 1H, N-*H_hydrazide_*), 9.96 (s, 1H, N-*H*), 8.59 (s, 1H, C-*H*_imine_), 8.51 (d, *J* = 8.95 Hz, 1H, Ar-*H*), 8.40 (s, 1H, Ar-*H*), 8.02 (s, 1H, Ar-*H*), 7.99 (d, *J* = 3.57 Hz, 2H, Ar-*H*), 7.67 (d, *J* = 8.92 Hz, 1H, Ar-*H*), 7.57 (d, *J* = 8.18 Hz, 2H, Ar-*H*), 7.51 (d, *J* = 8.58 Hz, 2H, Ar-*H*), 7.17 (d, *J* = 5.28 Hz, 1H, Ar-*H*), 6.86 (d, *J* = 8.22 Hz, 2H, Ar-*H*); ^13^C NMR (100.6 MHz, DMSO-*d*_6_) (*δ*, ppm): 172.49, 162.67, 159.86, 151.03, 148.49, 148.29, 143.72, 135.37, 129.56, 129.27, 128.84, 126.72, 126.21, 125.83, 125.37, 121.33, 118.93, 116.18, 103.74; HRMS (*m*/*z*) calcd. for C_23_H_17_ClN_4_O_2_: 416.1040. Found: 417.1120 [M + H]^+^.

(n) (*E*)-4-((7-chloroquinolin-4-yl)amino)-*N*′-(4-(dimethylamino)benzylidene)benzo- hydrazide (**HD14**).


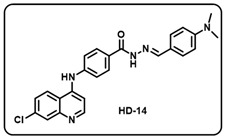


Light yellow solid; yield 77%; R*_f_* = 0.24 (methanol/dichloromethane = 5:95); mp: 290.9 °C; FT-IR (cm^−1^): 3294, 2894, 2798, 2321, 2103, 1908, 1646, 1568, 1509, 1448, 1363, 1218, 1016; ^1^H NMR (400 MHz, DMSO-*d*_6_) (*δ*, ppm): 11.53 (s, 1H, N-*H_hydrazide_*), 9.36 (s, 1H, N-*H*), 8.58 (d, *J* = 5.05 Hz, 1H, Ar-*H*), 8.43 (d, *J* = 9.05 Hz, 1H, Ar-*H*), 8.33 (s, 1H, C-*H*_imine_), 7.97 (d, *J* = 8.69 Hz, 3H, Ar-*H*), 7.62 (dd, *J* = 8.95 Hz, 2.03 Hz, 1H, Ar-*H*), 7.55 (d, *J* = 8.52 Hz, 2H, Ar-*H*), 7.48 (d, *J* = 8.29 Hz, 2H, Ar-*H*), 7.20 (d, *J* = 4.98 Hz, 1H, Ar-*H*), 6.76 (d, *J* = 8.56 Hz, 2H, Ar-*H*), 2.98 (s, 6H, 2C*H*_3_); ^13^C NMR (100.6 MHz, DMSO-*d*_6_) (*δ*, ppm): 162.52, 152.50, 151.92, 150.07, 148.67, 147.19, 144.23, 134.59, 129.45, 128.84, 128.34, 128.20, 125.81, 125.10, 122.18, 120.61, 119.40, 112.28, 104.17, 40.61; HRMS (*m*/*z*) calcd. for C_25_H_22_ClN_5_O: 443.1513. Found: 444.1596 [M + H]^+^.

(o) (*E*)-4-((7-chloroquinolin-4-yl)amino)-*N*′-(4-isopropylbenzylidene)benzohydrazide (**HD15**).


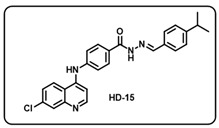


White solid; yield 86%; R*_f_* = 0.62 (methanol/dichloromethane = 5:95); mp: 274.4 °C; FT-IR (cm^−1^): 3315, 2959, 2189, 1648, 1559, 1523, 1456, 1370, 1326, 1269, 1190; ^1^H NMR (400 MHz, DMSO-*d*_6_) (*δ*, ppm): 11.78 (s, 1H, N-*H_hydrazide_*), 8.60 (d, *J* = 4.80 Hz, 1H, Ar-*H*), 8.46 (s, 1H, Ar-H), 8.44 (s, 1H, C-*H*_imine_), 8.00–7.97 (m, 3H, Ar-*H*), 7.67–7.65 (m, 3H, Ar-*H*), 7.51 (d, *J* = 8.41 Hz, 2H, Ar-*H*), 7.35 (d, *J* = 7.70 Hz, 2H, Ar-*H*), 7.20 (d, *J* = 5.28 Hz, 1H, Ar-*H*), 2.97–2.90 (m, 1H, C-*H*), 1.23 (d, *J* = 6.84 Hz, 6H, 2C*H*_3_); ^13^C NMR (100.6 MHz, DMSO-*d*_6_) (*δ*, ppm): 162.86, 151.85, 151.05, 149.29, 147.89, 144.68, 114.27, 134.94, 132.59, 129.62, 128.21, 127.60, 127.27, 126.01, 125.22, 120.82, 119.26, 104.10, 33.85, 24.13; HRMS (*m*/*z*) calcd. for C_26_H_23_ClN_4_O: 442.1560. Found: 443.1642 [M + H]^+^.

(p) (*E*)-*N*′-((1H-indol-3-yl)methylene)-4-((7-chloroquinolin-4-yl)amino) benzohydrazide (**HD16**).


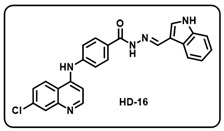


Light yellow solid; yield 81%; R*_f_* = 0.18 (methanol/dichloromethane = 5:95); mp: 268.3 °C; FT-IR (cm^−1^): 3633, 3426, 3281, 3177, 2825, 1910, 1609, 1568, 1519, 1438, 1364, 1307, 1248; ^1^H NMR (400 MHz, DMSO-*d*_6_) (*δ*, ppm): 11.63 (s, 1H, N-*H*_Indol ring_), 11.58 (s, 1H, N-*H_hydrazide_*), 9.76 (s, 1H, N-*H*), 8.67 (s, 1H, C-*H*_imine_), 8.59 (d, *J* = 5.34 Hz, 1H, Ar-*H*), 8.53 (d, *J* = 8.95 Hz, 1H, Ar-*H*), 8.34 (d, *J* = 7.46 Hz, 1H, Ar-*H*), 8.04 (d, *J* = 8.45 Hz, 2H, Ar-*H*), 7.99 (d, *J* = 1.70 Hz, 1H, Ar-*H*), 7.89 (d, *J* = 2.39 Hz, 1H, Ar-*H*), 7.68 (d, *J* = 8.95 Hz, 1H, Ar-*H*), 7.53 (d, *J* = 8.49 Hz, 2H, Ar-*H*), 7.46 (d, *J* = 8.18 Hz, 1H, Ar-*H*), 7.23–7.15 (m, 3H, Ar-*H*); ^13^C NMR (100.6 MHz, DMSO-*d*_6_) (*δ*, ppm): 162.34, 155.55, 150.91, 148.74, 148.05, 145.20, 143.68, 137.52, 135.44, 132.29, 130.70, 129.48, 126.58, 126.23, 125.40, 124.84, 123.08, 122.52, 121.56, 120.82, 11.88, 112.26, 103.55; HRMS (*m*/*z*) calcd. for C_25_H_18_ClN_5_O: 439.1200. Found: 440.1277 [M + H]^+^.

(q) (*E*)-4-((7-chloroquinolin-4-yl)amino)-*N*′-(4-methylbenzylidene)benzohydrazide (**HD17**).


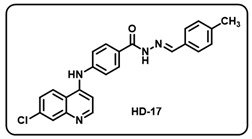


Deep yellow solid; yield 84%; R*_f_* = 0.44 (methanol/dichloromethane = 5:95); mp: 283.6 °C; FT-IR (cm^−1^): 3304, 3020, 2342, 2112, 1917, 1644, 1560, 1503, 1449, 1368; ^1^H NMR (400 MHz, DMSO-*d*_6_) (*δ*, ppm): 11.83 (s, 1H, N-*H_hydrazide_*), 9.72 (s, 1H, N-*H*), 8.60 (d, *J* = 4.19 Hz, 1H, Ar-*H*), 8.51 (d, *J* = 9.33 Hz, 1H, Ar-*H*), 8.47 (s, 1H, C-*H*_imine_), 8.01 (t, *J* = 8.39 Hz, 3H, Ar-*H*), 7.68–7.62 (m, 3H, Ar-*H*), 7.52 (d, *J* = 8.22 Hz, 2H, Ar-*H*), 7.27 (d, *J* = 7.71 Hz, 2H, Ar-*H*), 7.18 (d, *J* = 5.40 Hz, 1H, Ar-*H*), 2.35 (s, 3H, C*H*_3_); ^13^C NMR (100.6 MHz, DMSO-*d*_6_) (*δ*, ppm): 162.83, 161.69, 151.06, 148.45, 148.25, 147.97, 143.89, 140.27, 135.37, 132.18, 129.91, 129.65, 128.78, 127.49, 126.22, 125.38, 121.27, 118.86, 103.80, 21.50; HRMS (*m*/*z*) calcd. for C_24_H_19_ClN_4_O: 414.1247. Found: 415.1326 [M + H]^+^.

(r) (*E*)-4-((7-chloroquinolin-4-yl)amino)-*N*′-(4-hydroxy-3-methoxybenzylidene)benzo-hydrazide (**HD18**).


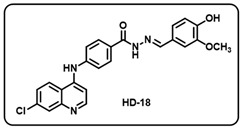


Off-white solid; yield 85%; R*_f_* = 0.18 (methanol/dichloromethane = 5:95); mp: 191.8 °C; FT-IR (cm^−1^): 3204, 3029, 2037, 2592, 2344, 2112, 1920, 1702, 1649, 1590, 1536, 1263, 1037; ^1^H NMR (400 MHz, DMSO-*d*_6_) (*δ*, ppm): 11.68 (s, 1H, N-*H_hydrazide_*), 9.58 (s, 1H, N-*H*), 8.59 (d, *J* = 5.42 Hz, 1H, Ar-*H*), 8.47 (d, *J* = 9.24 Hz, 1H, Ar-*H*), 8.38 (s, 1H, C-*H*_imine_), 8.00–7.97 (m, 3H, Ar-*H*), 7.65 (dd, *J* = 9.02 Hz, 2.08 Hz, 1H, Ar-*H*), 7.50 (d, *J* = 8.68 Hz, 2H, Ar-*H*), 7.33 (s, 1H, Ar-*H*), 7.18 (d, *J* = 5.41 Hz, 1H, Ar-*H*), 7.10 (d, *J* = 7.70 Hz, 1H, Ar-*H*) 6.86 (d, *J* = 8.03 Hz, 1H, Ar-*H*), 3.84 (s, 3H, OC*H*_3_); ^13^C NMR (100.6 MHz, DMSO-*d*_6_) (*δ*, ppm): 172.49, 162.71, 151.71, 149.41, 149.07, 148.51, 147.87, 144.06, 135.02, 129.55, 128.49, 127.04, 126.28, 126.04, 125.24, 122.57, 120.97, 119.17, 115.91, 109.36, 103.98, 56.00; HRMS (*m*/*z*) calcd. for C_24_H_19_ClN_4_O_3_: 446.1146. Found: 447.1227 [M + H]^+^.

(s) (*E*)-4-((7-chloroquinolin-4-yl)amino)-*N*′-(thiophen-2-ylmethylene)benzohydrazide (**HD19**).


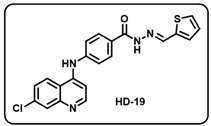


Light yellow solid; yield 87%; R*_f_* = 0.40 (methanol/dichloromethane = 5:95); mp: 254.6 °C; FT-IR (cm^−1^): 3408, 3211, 2073, 1548, 1563, 1524, 1462, 1370, 1325, 1271, 1014; ^1^H NMR (400 MHz, DMSO-*d*_6_) (*δ*, ppm): 11.82 (s, 1H, N-*H_hydrazide_*), 9.50 (s, 1H, N-*H*), 8.71 (s, 1H, C-*H*_imine_), 8.59 (d, *J* = 5.36 Hz, 1H, Ar-*H*), 8.46 (d, *J* = 9.16 Hz, 1H, Ar-*H*), 7.99 (s, 1H, Ar-*H*), 7.97 (d, *J* = 1.97 Hz, 2H, Ar-*H*), 7.68 (d, *J* = 4.78 Hz, 1H, Ar-*H*), 7.64 (dd, *J* = 9.03 Hz, 2.13 Hz, 1H, Ar-*H*), 7.51–7.46 (m, 3H, Ar-*H*), 7.20 (d, *J* = 5.30 Hz, 1H, Ar-*H*), 7.15 (t, *J* = 3.92 Hz, 1H, Ar-*H*); ^13^C NMR (100.6 MHz, DMSO-*d*_6_) (*δ*, ppm): 172.50, 162.81, 152.04, 149.52, 147.49, 144.42, 142.99, 139.74, 134.84, 131.22, 129.60, 129.29, 128.32, 127.75, 125.96, 125.20, 120.71, 119.95, 104.21; HRMS (*m*/*z*) calcd. for C_21_H_15_ClN_4_OS: 406.0655. Found: 407.0737 [M + H]^+^.

(t) (*E*)-4-((7-chloroquinolin-4-yl)amino)-*N*′-(furan-2-ylmethylene)benzohydrazide (**HD20**).


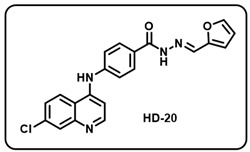


Deep yellow solid; yield 89%; R*_f_* = 0.29 (methanol/dichloromethane = 5:95); mp: 246.7 °C; FT-IR (cm^−1^): 2765, 2357, 2166, 1863, 1622, 1679, 1529, 1445, 1398, 1336, 1293; ^1^H NMR (400 MHz, DMSO-*d*_6_) (*δ*, ppm): 11.83 (s, 1H, N-*H_hydrazide_*), 8.60 (d, *J* = 5.27 Hz, 1H, Ar-*H*), 8.48 (d, *J* = 9.06 Hz, 1H, Ar-*H*), 8.39 (s, 1H, C-*H*_imine_), 8.00 (d, *J* = 8.78 Hz, 3H, Ar-*H*), 7.86 (s, 1H, Ar-*H*), 7.67 (dd, *J* = 8.98, 1.90 Hz, 1H, Ar-*H*), 7.52 (d, *J* = 8.44, 2H, Ar-*H*), 7.18 (d, *J* = 5.49 Hz, 1H, Ar-*H*), 6.94 (d, *J* = 2.56 Hz, 1H, Ar-*H*), 6.65 (s, 1H, Ar-H); ^13^C NMR (100.6 MHz, DMSO-*d*_6_) (*δ*, ppm): 172.49, 162.84, 151.35, 150.00, 148.13, 145.58, 144.10, 137.69, 135.22, 129.63, 128.29, 127.04, 126.15, 125.31, 121.08, 119.09, 113.79, 112.66, 103.96; HRMS (*m*/*z*) calcd. for C_21_H_15_ClN_4_O_2_: 390.0884. Found: 391.968 [M + H]^+^.

(u) (*E*)-4-((7-chloroquinolin-4-yl)amino)-*N*′-(pyridin-2-ylmethylene)benzohydrazide (**HD21**).


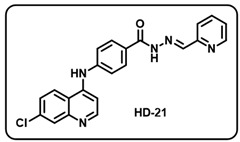


Deep yellow solid; yield 81%; R*_f_* = 0.16 (methanol/dichloromethane = 5:95); mp: 185.8 °C; FT-IR (cm^−1^): 3259, 2997, 2886, 2342, 2105, 1920, 1654, 1578, 1528, 1501, 1425, 1285, 1050; ^1^H NMR (400 MHz, DMSO-*d*_6_) (*δ*, ppm): 15.59 (s, 1H, N-*H_hydrazide_*), 8.94 (d, *J* = 4.21 Hz, 1H, Ar-*H*), 8.62 (d, *J* = 5.53 Hz, 1H, Ar-*H*), 8.49 (d, *J* = 9.09 Hz, 1H, Ar-*H*), 8.13 (ddd, *J* = 15.51, 7.82, 1.51 Hz, 1H, Ar-*H*), 8.01–7.99 (m, 3H, Ar-*H*), 7.84 (d, *J* = 7.93, 1H, Ar-*H*), 7.72 (s, 1H, C-*H*_imine_), 7.67 (dd, *J* = 11.05, 2.01 Hz, 1H, Ar-*H*), 7.58 (m, 3H, Ar-*H*), 7.27 (d, *J* = 5.56 Hz, 1H, Ar-*H*); ^13^C NMR (100.6 MHz, DMSO-*d*_6_) (*δ*, ppm): 172.49, 152.50, 151.34, 149.97, 148.90, 147.92, 139.16, 137.32, 135.27, 129.79, 129.29, 127.58, 126.96, 126.24, 125.39, 125.14, 121.22, 119.23, 104.50, 103.98; HRMS (*m*/*z*) calcd. for C_22_H_16_ClN_5_O: 401.1043. Found: 402.1128 [M + H]^+^.

(v) (*E*)-*N*′-((1H-pyrrol-2-yl)methylene)-4-((7-chloroquinolin-4-yl)amino)benzo- hydrazide (**HD22**).


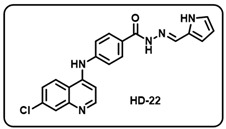


Gray solid; yield 85%; R*_f_* = 0.45 (methanol/dichloromethane = 5:95); mp: 234.0 °C; FT-IR (cm^−1^): 3414, 3301, 3295, 2849, 2342, 2100, 1608, 1569, 1520, 1445, 1373, 1328, 1032; ^1^H NMR (400 MHz, DMSO-*d*_6_) (*δ*, ppm): 11.51 (s, 1H, N-*H_hydrazide_*), 8.58 (d, *J* = 5.18 Hz, 1H, Ar-*H*), 8.44 (d, *J* = 9.10 Hz, 1H, Ar-*H*), 8.31 (s, 1H, C-*H*_imine_), 7.97 (s, 1H, Ar-*H*), 7.96 (d, *J* = 1.77 Hz, 2H, Ar-*H*), 7.63 (dd, *J* = 9.05, 2.15 Hz, 1H, Ar-*H*), 7.47 (d, *J* = 8.56 Hz, 2H, Ar-*H*), 7.18 (d, *J* = 5.18 Hz, 1H, Ar-*H*), 6.92 (d, *J* = 1.23 Hz, 1H, Ar-*H*), 6.48 (s, 1H, Ar-*H*), 6.15 (q, *J* = 5.42 Hz, 1H, Ar-*H*); ^13^C NMR (100.6 MHz, DMSO-*d*_6_) (*δ*, ppm): 172.49, 162.48, 152.23, 149.75, 147.43, 144.99, 134.73, 129.47, 128.44, 127.93, 127.61, 125.88, 125.14, 122.88, 120.74, 119.32, 113.62, 109.70, 104.08; HRMS (*m*/*z*) calcd. for C_21_H_16_ClN_5_O: 389.1043. Found: 390.1125 [M + H]^+^.

(w) (*E*)-4-((7-chloroquinolin-4-yl)amino)-*N*′-(quinolin-2-ylmethylene)benzohydrazide (**HD23**).


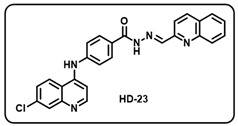


Off-white solid; yield 87%; R*_f_* = 0.32 (methanol/dichloromethane = 5:95); mp: 288.7 °C; FT-IR (cm^−1^): 3305, 2705, 2342, 1659, 1607, 1568, 1520, 1451, 1351, 1258, 1155; ^1^H NMR (400 MHz, DMSO-*d*_6_) (*δ*, ppm): 12.20 (s, 1H, N-*H_hydrazide_*), 9.60 (s, 1H, N-*H*), 8.64 (s, 1H, C-*H*_imine_), 8.61 (d, *J* = 5.20 Hz, 1H, Ar-*H*), 8.46 (d, *J* = 9.14 Hz, 1H, Ar-*H*), 8.42 (d, *J* = 8.49 Hz, 1H, Ar-*H*), 8.14 (d, *J* = 7.49 Hz, 1H, Ar-*H*), 8.06–8.00 (m, 4H, Ar-*H*), 7.97 (d, *J* = 2.01 Hz, 1H, Ar-*H*), 7.79 (t, *J* = 7.26 Hz, 1H, Ar-*H*), 7.67–7.62 (m, 2H, Ar-*H*), 7.53 (d, *J* = 8.51 Hz, 2H, Ar-*H*), 7.21 (d, *J* = 5.36 Hz, 1H, Ar-*H*); ^13^C NMR (100.6 MHz, DMSO-*d*_6_) (*δ*, ppm): 163.46, 154.36, 151.61, 148.94, 147.85, 137.19, 135.11, 130.54, 129.89, 129.37, 128.49, 128.35, 127.76, 127.32, 126.13, 125.27, 120.87, 119.23, 117.95, 104.23; HRMS (*m*/*z*) calcd. for C_26_H_18_ClN_5_O: 451.1200. Found: 452.1282 [M + H]^+^.

#### 5.1.4. Synthesis of Substituted Isonitrosoacetanilides (**5a**–**l**)

Substituted aniline **3a**–**l** (4.29 mmol) was taken in a dry RB flask and incorporated with water (23 mL) followed by the addition of 2 N HCl (1.72 mL). When aniline was completely dissolved, reagents were added in the following order: anhyd. sodium sulfate (28.35 mmol), hydroxylamine hydrochloride (15.03 mmol), and chloral hydrate (15.03 mmol). The reaction mixture was allowed to mix uniformly and then allowed to stir overnight at 55 °C. Reaction progress was monitored by TLC. After completion of the reaction, mixture was cooled to room temperature. Without additional purification, the solid crude was filtered under suction and washed with cold distilled water and dried subsequently to yield substituted isonitrosoacetanilides (**5a**–**l**), which proceeded to next step to make substituted isatin derivatives [52,53]. 

#### 5.1.5. Synthesis of Substituted Isatin (**6a**–**l**)

Specified amount of pure sulfuric acid (1.08 mL) was taken in a dried two-neck round-bottom flask and warmed to 50 °C, then added to isonitrosoacetanilide **5a**–**l** (1.52 mmol), and the reaction temperature was gradually increased and kept between 60 and 70 °C. Subsequently, the reaction mixture was kept heated at 80 °C for 15–20 min and then allowed to cool down to room temperature after the reaction, which was monitored by TLC. The product mixture was poured into ice water, resulting in the formation of precipitate. The solid precipitate was suction-filtered, washed with cold water until the sulfuric acid was entirely removed, and dried to obtain high-purity substituted isatins (**6a**–**l**) [52,53]. 

#### 5.1.6. Synthesis of CQ-Isatin Hydrazone Hybrids (**HS1-12**)

Separately in two dried 50 mL round-bottom flasks, appropriate isatin derivatives (**6a**–**l**) and the linker LN-2 (1.0 equiv. each) were dissolved in ethanol respectively. The two were mixed, and the reaction mixture was refluxed at 80 °C for 8 h. Reaction progress was monitored by TLC. After completion of the reaction, the reaction mixture was allowed to cool to room temperature then followed by the addition of ice-cold water, and precipitate formed. This precipitate was filtered and washed with cold water to obtain the desired product (**HS1-12**) in good to excellent yield [52,53,55]. 

(a) (*Z*)-4-((7-chloroquinolin-4-yl)amino)-*N*′-(2-oxoindolin-3-ylidene)benzohydrazide (**HS1**).


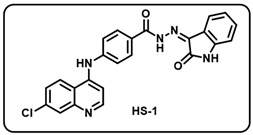


Yellow solid; yield 75%; R*_f_* = 0.30 (methanol/dichloromethane = 10:90); mp: 264–266 °C; FT-IR (cm^−1^): 3319, 3230, 1739, 1699, 1654, 1344, 1264; ^1^H NMR (400 MHz, DMSO-*d*_6_) (δ, ppm): 13.94 (s, 1H, N-*H*_hydrazide_), 11.37 (s, 1H, N-*H*_pyrolering_), 9.50 (s, 1H, N-*H*), 8.62 (d, *J* = 5.03 Hz, 1H, Ar-*H*), 8.41 (d, *J* = 8.95 Hz, 1H, Ar-*H*), 7.97–7.91 (m, 3H, Ar-*H*), 7.64–7.60 (m, 2H, Ar-*H*), 7.55 (d, *J* = 8.4 Hz, 2H, Ar-*H*), 7.41 (t, *J* = 7.6 Hz, 1H, Ar-*H*), 7.30 (d, *J* = 5.2 Hz, 1H, Ar-*H*), 7.14 (t, *J* = 7.2 Hz, 1H, Ar-*H*), 6.98 (d, *J* = 7.6 Hz, 1H, Ar-*H*); ^13^C NMR (100.6 MHz, DMSO-*d*_6_) (δ, ppm): 163.58, 152.36, 149.89, 146.73, 145.91, 142.79, 138.07, 134.77, 132.10, 130.65, 129.59, 128.06, 126.06, 125.87, 125.19, 123.21, 120.38 (d, *J* = 4.03 Hz), 120.13, 119.69, 11.69, 105.33; ESI-MS (*m/z*) calc. for C_24_H_16_ClN_5_O_2_: 441.09; found 442.10 [M + H]^+^.

(b) (*Z*)-4-((7-chloroquinolin-4-yl)amino)-*N*′-(7-methyl-2-oxoindolin-3-ylidene)benzohydrazide (**HS2**).


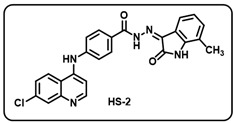


Pale yellow solid; yield 78% yield; R*_f_* = 0.44 (methanol/dichloromethane = 10:90); mp: 266–268 °C; FT-IR (cm^−1^): 3231, 1710, 1692, 1681, 1352, 1230; ^1^H NMR (400 MHz, DMSO-*d*_6_) (δ, ppm): 13.96 (s, 1H, N-*H*_hydrazide_), 11.27 (s, 1H, N-*H*_pyrolering_), 9.64 (s, 1H, N-*H*), 8.63 (d, *J* = 4.04 Hz, 1H, Ar-*H*), 8.47–8.42 (m, 1H, Ar-*H*), 8.05 (d, *J* = 8.24 Hz, 1H, Ar-*H*), 7.98–7.92 (m, 2H, Ar-*H*), 7.68 (d, *J* = 8.72 Hz, 1H, Ar-*H*), 7.57–7.52 (m, 2H, Ar-*H*), 7.45 (s, 1H, Ar-*H*), 7.30–7.20 (m, 2H, Ar-*H*), 6.87–6.81 (m, 1H, Ar-*H*), 2.32 (s, 3H, CH_3_); ^13^C NMR (100.6 MHz, DMSO-*d*_6_) (δ, ppm): 163.57, 159.48, 159.10, 158.72, 158.35, 154.69, 144.54, 141.56, 140.69, 139.46 (d, *J* = 22.13 Hz), 132.74 (d, *J* = 40.62 Hz), 130.73, 129.68, 128.22, 126.21, 125.33, 121.71, 120.15 (d, *J* = 26.20 Hz), 117.13, 116.79, 114.29, 111.50, 101.63, 20.92; ESI-MS (*m/z*) calc. for C_25_H_18_ClN_5_O_2_: 455.11; found 456.05 [M + H]^+^.

(c) (*Z)*-4-((7-chloroquinolin-4-yl)amino)-*N*′-(7-nitro-2-oxoindolin-3-ylidene)benzohydrazide (**HS3**).


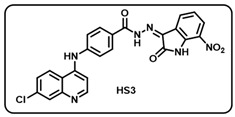


Pale yellow solid; yield 70%; R*_f_* = 0.45 (methanol/dichloromethane = 10:90); mp: 272–274 °C; FT-IR (cm^−1^): 3330, 3160, 1723, 1698, 1680, 1328, 1270; ^1^H NMR (400 MHz, DMSO-*d*_6_, TFA-*d*_1_) (δ, ppm): 13.93 (s, 1H, N-*H*_hydrazide_), 11.50 (s, 1H, N-*H*_pyrolering_), 8.82–8.72 (m, 2H, Ar-*H*), 8.35 (dd, *J* = 7.14, 1.53 Hz, 1H, Ar-*H*), 8.10–8.04 (m, 2H, Ar-*H*), 7.93–7.83 (m, 1H, Ar-*H*), 7.71–7.69 (m, 2H, Ar-H), 7.60 (d, *J* = 8.05 Hz, 1H, Ar-*H*), 7.15–7.09 (m, 2H, Ar–H); ^13^C NMR (100.6 MHz, DMSO-*d*_6_, TFA-*d*_1_) (δ, ppm): 163.65, 157.44, 156.98, 156.57, 144.12, 143.95, 140.92, 139.36, 136.47, 129.73, 128.21, 126.74, 125.49, 125.27, 122.96, 122.58, 119.62, 118.78, 115.95, 113.91, 110.27; ESI-MS (*m/z*) calc. for C_24_H_15_ClN_6_O_4_: 486.08; found 487.08 [M + H]^+^.

(d) (*Z*)-4-((7-chloroquinolin-4-yl)amino)-*N*′-(5-methyl-2-oxoindolin-3-ylidene)benzohydrazide (**HS4**).


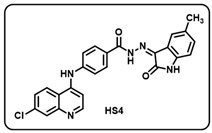


Yellow solid; yield 80%; R*_f_* = 0.45 (methanol/dichloromethane = 10:90); mp: 268–270 °C; FT-IR (cm^−1^): 3231, 1710, 1692, 1681, 1352, 1230; ^1^H NMR (400 MHz, DMSO-*d*_6_) (δ, ppm): 13.83 (s, 1H, N-*H*_hydrazide_), 11.53 (s, 1H, N-*H*_pyrolering_), 9.60 (s, 1H, N-*H*), 8.61 (s, 1H, Ar-*H*), 8.43 (d, *J* = 8.53 Hz, 1H, Ar-*H*), 7.97–7.91 (m, 3H, Ar-*H*), 7.66–7.54 (m, 4H, Ar-*H*), 7.29 (d, *J* = 4.38 Hz, 1H, Ar-*H*), 6.93–6.81 (m, 2H, Ar-*H*), 1.91 (s, 3H, C*H*_3_); ^13^C NMR (100.6 MHz, DMSO-*d*_6_) (δ, ppm): 166.01, 163.76 (d, *J* = 21.38 Hz), 154.35, 144.92 (d, *J* = 12.14 Hz), 141.84, 139.94, 139.02, 130.41, 129.73, 128.14, 126.22, 125.14, 123.45 (d, *J* = 10.53 Hz), 120.27, 116.95, 116.62, 110.09 (d, *J* = 23.93 Hz), 101.86, 100.17 (d, *J* = 27.64 Hz), 21.50; ESI-MS (*m/z*) calc. for C_25_H_18_ClN_5_O_2_: 455.11; found 456.05 [M + H]^+^.

(e) (*Z*)-*N*′-(5-chloro-2-oxoindolin-3-ylidene)-4-((7-chloroquinolin-4-yl)amino)benzohydrazide (**HS5**).


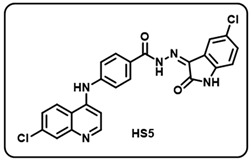


Yellow solid; yield 85%; R*_f_* = 0.39 (methanol/dichloromethane = 10:90); mp: 274–276 °C; FT-IR (cm^−1^): 3239, 1715, 1689, 1681, 1352, 1217; ^1^H NMR (400 MHz, DMSO-*d*_6_, TFA-*d*_1_) (δ, ppm): 13.95 (s, 1H, N-*H*_hydrazide_), 11.79 (s, 1H, N-*H*_pyrolering_), 11.09 (s, 1H, N-*H*), 8.75 (d, *J* = 9.04 Hz, 1H, Ar-*H*), 8.65 (d, *J* = 6.8 Hz, 1H, Ar-*H*), 8.10–8.05 (m, 3H, Ar-*H*), 7.88 (d, *J* = 6.98 Hz, 1H, Ar-*H*), 7.74 (d, *J* = 7.81 Hz, 2H, Ar-*H*), 7.57 (d, *J* = 6.80 Hz, 1H, Ar-*H*), 7.46–7.43 (m, 1H, Ar-*H*), 7.15–7.10 (m, 2H, Ar-*H*); ^13^C NMR (100.6 MHz, DMSO-*d*_6_, TFA-*d*_1_) (δ, ppm): 163.55, 154.75, 144.43, 141.67, 140.21, 139.43 (d, *J* = 13.73 Hz), 131.64, 130.54, 129.76, 128.14, 126.13, 125.28, 124.21, 122.17, 119.83, 116.85, 115.88, 111.12, 101.52; ESI-MS (*m/z*) calc. for C_24_H_15_Cl_2_N_5_O_2_: 475.06; found 476.10 [M + H]^+^.

(f) (*Z)*-*N*′-(7-chloro-2-oxoindolin-3-ylidene)-4-((7-chloroquinolin-4-yl)amino) benzohydrazide (**HS6**).


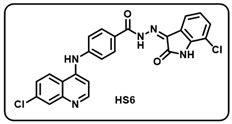


Yellow solid; yield 84%; R*_f_* = 0.41 (methanol/dichloromethane = 10:90); mp: 264–266 °C; FT-IR (cm^−1^): 3241, 1720, 1683, 1679, 1347, 1219; ^1^H NMR (400 MHz, DMSO-*d*_6_, TFA-*d*_1_) (δ, ppm): 13.66 (s, 1H, N-*H*_hydrazide_), 11.73 (s, 1H, N-*H*_pyrolering_), 11.01 (s, 1H, N-*H*), 8.68 (d, *J* = 8.87 Hz, 1H, Ar-*H*), 8.61 (d, *J* = 6.8 Hz, 1H, Ar-*H*), 8.07–8.01 (m, 3H, Ar-*H*), 7.88 (d, *J* = 6.98 Hz, 1H, Ar-*H*), 7.74 (d, *J* = 7.81 Hz, 2H, Ar-*H*), 7.57 (d, *J* = 6.80 Hz, 1H, Ar-*H*), 7.43 (s, 1H, Ar-*H*), 7.19–7.14 (m, 2H, Ar-*H*); ^13^C NMR (100.6 MHz, DMSO-*d*_6_, TFA-*d*_1_) (δ, ppm): 163.64, 154.73, 144.41, 141.52, 140.11, 139.38, 131.76, 130.28, 129.64, 128.19, 126.11, 125.27, 124.37, 122.13, 119.76, 116.09, 115.83, 111.26, 101.67; ESI-MS (*m/z*) calc. for C_24_H_15_Cl_2_N_5_O_2_: 475.06; found 476.10 [M + H]^+^. 

(g) (*Z*)-*N*′-(5-bromo-2-oxoindolin-3-ylidene)-4-((7-chloroquinolin-4-yl)amino)benzohydrazide (**HS7**).


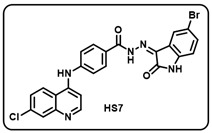


Pale yellow solid; yield 82%; R*_f_* = 0.43 (methanol/dichloromethane = 10:90); mp: 270–272 °C; FT-IR (cm^−1^): 3235, 1721, 1687, 1679, 1354, 1223; ^1^H NMR (400 MHz, DMSO-*d*_6_) (δ, ppm): 13.95 (s, 1H, N-*H*_hydrazide_), 11.58 (s, 1H, N-*H*_pyrolering_), 11.15 (s, 1H, N-*H*), 8.79 (d, *J* = 9.18 Hz, 1H, Ar-*H*), 8.71 (d, *J* = 6.95 Hz, 1H, Ar-*H*), 8.12–8.10 (m, 3H, Ar-*H*), 7.96 (dd, *J* = 9.07, 1.80 Hz, 1H, Ar-*H*), 7.78 (d, *J* = 8.48 Hz, 2H, Ar-*H*), 7.58 (s, 1H, Ar-*H*), 7.48 (dd, *J* = 8.32, 2.03 Hz, 1H, Ar-*H*), 7.20 (d, *J* = 6.97 Hz, 1H, Ar-*H*), 7.04 (d, *J* = 8.35 Hz, 1H, Ar-*H*); ^13^C NMR (100.6 MHz, DMSO-*d*_6_) (δ, ppm): 163.28, 154.68, 144.55, 141.71 (d, *J* = 12.15 Hz), 139.46, 39.25, 131.69, 130.46, 129.86, 128.24, 127.40, 126.24, 125.31, 121.86, 120.87, 119.99, 117.11, 113.26, 101.68; ESI-MS (*m/z*) calc. for C_24_H_15_BrClN_5_O_2_: 519.00; found 520.02 [M + H]^+^.

(h) (*Z*)-4-((7-chloroquinolin-4-yl)amino)-*N*′-(5-methoxy-2-oxoindolin-3-ylidene)benzohydrazide (**HS8**).


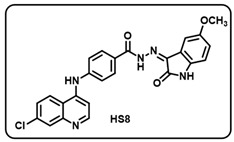


Yellow solid; yield 83%; R*_f_* = 0.37 (methanol/dichloromethane = 10:90); mp: 284–286 °C; FT-IR (cm^−1^): 3233, 1716, 1686, 1651, 1315, 1209; ^1^H NMR (400 MHz, DMSO-*d*_6_, TFA-*d*_1_) (δ, ppm): 14.15 (s, 1H, N-*H*_hydrazide_), 11.25 (s, 1H, N-*H*_pyrolering_), 11.13 (s, 1H, N-*H*), 8.80 (d, *J* = 9.25 Hz, 1H, Ar-*H*), 8.70 (d, *J* = 6.99 Hz, 1H, Ar-*H*), 8.14–8.10 (m, 3H, Ar-*H*), 7.95 (dd, *J* = 9.08, 1.97 Hz, 1H, Ar-*H*), 7.78 (d, *J* = 8.55 Hz, 2H, Ar-*H*), 7.18 (d, *J* = 6.94 Hz, 2H, Ar-*H*), 7.02 (dd, *J* = 8.54, 2.54 Hz, 1H, Ar-*H*), 6.94 (d, *J* = 8.52 Hz, 1H, Ar-*H*), 3.83 (s, 3H, OCH_3_); ^13^C NMR (100.6 MHz, DMSO-*d*_6_, TFA-*d*_1_) (δ, ppm): 163.63, 155.93, 154.80, 144.41, 141.56, 139.43 (d, *J* = 15.32 Hz), 136.60, 130.76, 129.79, 128.15, 126.14, 124.35, 120.90, 119.84 (d, *J* = 12.92 Hz), 118.39, 116.85 (d, *J* = 9.85 Hz), 113.99, 112.48, 111.13, 106.28, 101.51, 55.84; ESI-MS (*m/z*) calc. for C_25_H_18_ClN_5_O_3_: 471.12; found 472.08 [M + H]^+^.

(i) (*Z*)-*N*′-(6-chloro-2-oxoindolin-3-ylidene)-4-((7-chloroquinolin-4-yl)amino) benzohydrazide (**HS9**).


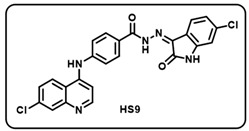


Yellow solid; yield 76%; R*_f_* = 0.45 (methanol/dichloromethane = 10:90); mp: 282–284 °C; FT-IR (cm^−1^): 3237, 1714, 1674, 1648, 1307, 1216; ^1^H NMR (400 MHz, DMSO-*d*_6_, TFA-*d*_1_) (δ, ppm): 8.79 (d, *J* = 9.15 Hz, 1H, Ar-*H*), 8.67 (d, *J* = 7.03 Hz, 1H, Ar-*H*), 8.14–8.08 (m, 3H, Ar-*H*), 7.88 (dd, *J* = 9.12, 2.05 Hz, 1H, Ar-*H*), 7.76 (d, *J* = 8.61 Hz, 2H, Ar-*H*), 7.59 (d, *J* = 7.41 Hz, 1H, Ar-*H*), 7.45 (d, *J* = 7.45 Hz, 1H, Ar-*H*), 7.18–7.11 (m, 2H, Ar-H); ^13^C NMR (100.6 MHz, DMSO-*d*_6_, TFA-*d*_1_) (δ, ppm): 163.35, 154.64, 144.10, 141.43, 140.02, 139.37 (d, *J* = 14.35 Hz), 131.52, 130.50, 129.65, 128.05, 126.01, 125.12, 124.03, 122.07, 119.67, 115.83, 101.27; ESI-MS (*m/z*) calc. for C_24_H_15_Cl_2_N_5_O_2_ 475.06; found 476.30 [M + H]^+^.

(j) (*Z*)-4-((7-chloroquinolin-4-yl)amino)-*N*′-(5,7-dimethyl-2-oxoindolin-3-ylidene) benzohydrazide (**HS10**).


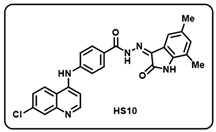


Yellow solid; yield 77%; R*_f_* = 0.43 (methanol/dichloromethane = 10:90); mp: 292–294 °C; FT-IR (cm^−1^): 3230, 1711, 1666, 1644, 1311, 1209; ^1^H NMR (400 MHz, DMSO-*d*_6_, TFA-*d*_1_) (δ, ppm): 8.70 (d, *J* = 9.2 Hz, 1H, Ar-*H*), 8.61 (d, *J* = 7.2 Hz, 1H, Ar-*H*), 8.03–7.98 (m, 3H, Ar-*H*), 7.81 (dd, *J* = 9.2, 2.4 Hz, 1H, Ar-*H*), 7.68 (d, *J* = 8.4 Hz, 2H, Ar-*H*), 7.13–7.08 (m, 2H, Ar-*H*), 6.93 (s, 1H, Ar-*H*), 2.23 (s, 3H, C*H*_3_), 2.15 (s, 3H, C*H*_3_); ^13^C NMR (100.6 MHz, DMSO-*d*_6_, TFA-*d*_1_) (δ, ppm): 160.06, 159.45 (q, *J* = 314.4 Hz, TFA–d_1_), 154.41, 144.36, 139.48, 139.39, 134.40, 132.03, 130.87, 129.63, 128.22, 126.14, 125.24, 120.94, 119.80, 101.45, 20.70, 16.16; ESI-MS (*m/z*) calc. C_26_H_20_ClN_5_O_2_: 463.13; found 464.06 [M + H]^+^.

(k) (*Z*)-4-((7-chloroquinolin-4-yl)amino)-*N*′-(6-fluoro-2-oxoindolin-3-ylidene)benzohydrazide (**HS11**).


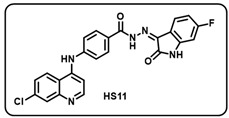


Pale yellow solid; yield 71%; R*_f_* = 0.45 (methanol/dichloromethane = 10:90); mp: 270–272 °C; FT-IR (cm^−1^): 3335, 3120, 1716, 1695, 1677, 1329, 1268; ^1^H NMR (400 MHz, DMSO-*d*_6_, TFA-*d*_1_) (δ, ppm): 13.63 (s, 1H, N-*H*_hydrazide_), 11.32 (s, 1H, N-*H*_pyrolering_), 11.08 (s, 1H, N-*H*), 8.81–8.75 (m, 2H, Ar-*H*), 8.32 (dd, *J* = 7.15, 1.78 Hz, 1H, Ar-*H*), 8.08–8.04 (m, 3H, Ar-*H*), 7.83–7.77 (m, 1H, Ar-*H*), 7.70–7.68 (m, 2H, Ar-*H*), 7.58 (d, *J* = 8.05 Hz, 1H, Ar-*H*), 7.13–7.09 (m, 2H, Ar-*H*); ^13^C NMR (100.6 MHz, DMSO-*d*_6_, TFA-*d*_1_) (δ, ppm): 163.61, 157.41, 156.94, 156.52, 144.10, 143.89, 141.12, 140.92, 139.36, 136.47, 129.73, 128.21, 126.74, 125.49, 125.27, 122.96, 122.58, 119.62, 118.78, 115.95, 113.91, 110.27; ESI-MS (*m/z*) calc. for C_24_H_15_ClFN_5_O_2_: 459.09 found 460.01 [M + H]^+^

(l) (*Z*)-4-((7-chloroquinolin-4-yl)amino)-*N*′-(6-methoxy-2-oxoindolin-3-ylidene)benzohydrazide (**HS12**).


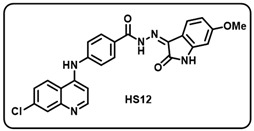


Light yellow solid; yield 70%; R*_f_* = 0.38 (methanol/dichloromethane = 10:90); mp: 323–325 °C; FT-IR (cm^−1^): 3328, 3157, 1722, 1687, 1663, 1314, 1243; ^1^H NMR (400 MHz, DMSO-*d*_6_, TFA-*d*_1_) (δ, ppm): 8.71 (d, *J* = 9.2 Hz, 1H, Ar-*H*), 8.60 (d, *J* = 7.2 Hz, 1H, Ar-*H*), 8.05–8.00 (m, 3H, Ar-*H*), 7.80 (d, *J* = 9.2 Hz, 1H, Ar-*H*), 7.68 (d, *J* = 8.4 Hz, 2H, Ar-*H*), 7.11–7.06 (m, 2H, Ar-*H*), 6.91 (dd, *J* = 8.8, 2.4 Hz, 1H, Ar-*H*), 6.83 (d, *J* = 8.4 Hz, 1H, Ar-*H*), 3.75 (s, 3H, OC*H*_3_); ^13^C NMR (100.6 MHz, DMSO-*d*_6_, TFA-*d*_1_) (δ, ppm): 163.61, 159.62 (q, *J* = 314.4 Hz, TFA–d_1_), 156.26, 154.95, 144.63, 141.60, 139.74, 136.74, 131.00, 129.91, 128.41, 126.32, 125.43, 121.13, 120.04, 118.51, 117.07, 112.68, 106.57, 101.59, 55.98; ESI-MS (*m/z*) calc. for C_25_H_18_ClN_5_O_3_: 471.08; found 472.14 [M + H]^+^.

### 5.2. Biological Evaluation

#### 5.2.1. Culture Preparation and Maintenance

Subculturing of four bacterial strains, namely, *E. faecium* (MTCC 439), *Bacillus subtilis* (MTCC 736), *S. aureus* (MTCC 902), and *P. aeruginosa* (ATCC 2453), was carried out on a nutrient agar medium using the streaking method. The streaking was performed on petri plates containing the nutrient agar. After completion, the plates were incubated at 37 °C for 24 h. The next day, the inoculum for primary culture was prepared by transferring a loopful of bacteria from the petri plates into test tubes containing 3 mL of nutrient broth. These test tubes were then incubated overnight in an incubator shaker at 37 °C.

For the secondary culture, a loopful of inoculum from the primary culture was transferred into new test tubes containing nutrient broth. These test tubes were incubated for 4–8 h at 37 °C [56].

#### 5.2.2. In Vitro Screening of Compounds

The objective of this experiment was to assess all 35 compounds (**HD1-23** and **HS1-12**) for their antibacterial activity against four different bacterial strains: *E. faecalis*, *B. subtilis*, *S. aureus*, and *P. aeruginosa*. Initially, stock solutions of the test compounds were prepared at a concentration of 10mg/mL in dimethyl sulfoxide (DMSO) to minimize solvent effects. The experiment was conducted using Mueller–Hinton agar (MHA) plates [57]. A suspension of each bacterial strain, approximately 10^5^ CFU/mL, was evenly spread across the agar surface using sterile glass beads to ensure uniform inoculation. Plates were marked to identify the test compounds. Sterile paper discs, soaked in the respective test compound solutions, were placed at predetermined distances on the agar using sterile forceps. Control discs included those infused with ciprofloxacin (CIP), those soaked in DMSO, and unsoaked discs, which were incorporated onto the plate as control. The plates were sealed with parafilm to prevent contamination and incubated at 37 °C overnight. Following this incubation, the plates were examined, and the diameters of the resulting zones of inhibition were measured. The antibacterial efficacies of the test compounds against the specified bacterial strains were thus determined.

#### 5.2.3. Minimum Inhibitory Concentration (MIC)

After conducting in vitro studies, the minimum inhibitory concentrations (MICs) of selected compounds were determined. Test compounds (**HD1**, **4**, **6**, **11**; **HS2**, **7**, **8**) were evaluated against the four bacterial isolates using a 96-well microtiter plate. The compounds were added to the wells containing nutrient broth, with the initial concentration set at 512 μg/mL in the first column. Serial dilutions were performed using a multichannel pipette, gradually reducing the concentration from 512 μg/mL to 2 μg/mL in subsequent wells. A 15 µL aliquot of bacterial culture, approximately 2 × 10^5^ Colony Forming Units (CFU)/mL [58], was added to each well. The setup included control groups: positive control, negative control, and color offset references. The plates were incubated at 37 °C with constant agitation at 90 rpm overnight in an incubator shaker. The MIC was determined as the lowest concentration of the compound that prevented observable bacterial growth, with all experiments conducted in biological triplicates [59,60].

#### 5.2.4. Minimum Bactericidal Concentration (MBC)

Following the determination of minimum inhibitory concentrations (MICs), a procedure was conducted to derive the minimum bactericidal concentrations (MBCs) of the test compounds. Samples of 4 μL, were drawn from each well absent of visible bacterial growth on the 96-well plate. These samples were then cultured onto the Mueller–Hinton agar (MHA) plates, avoiding exposure to the tested substances, and kept at a constant temperature of 37 °C overnight. The smallest concentration that prevented bacterial growth on the medium was identified as the MBC of an examined compound. A numerical correlation between these MBCs and MICs was then established through a calculated ratio. If the output ratio was ≤2, the substance was classified as bactericidal. However, if the ratio was >2, it indicated the substance had bacteriostatic properties [61].

#### 5.2.5. Disc Diffusion Assay

Diffusion assay was carried out by using the bacterial strains to assess the potential antimicrobial properties of the selected test compounds, particularly the ones that exhibited low MIC values in comparison to the other tested compounds against the bacterial isolates, as per the NARMS [62]. The assay was initialized by cultivating bacterial cells in a liquid broth medium and permitting overnight incubation at 37 °C. Following this, an inoculum of approximately 10^5^ cells/mL was extracted from the liquid broth culture and introduced into molten Mueller–Hinton agar (MHA) medium, which was subsequently dispensed into petri plates with a diameter of 90 mm. Upon solidification of the medium, sterile 4 mm diameter Whatman paper discs were placed at an appropriate distance on the solid agar surface [63]. Test compounds (**HD6** and **HS8**) equivalent to their ½ MIC, MIC, and 2 MIC concentrations were placed onto the discs that were then stationed on plates filled with MHA and uniform bacterial smears. Ciprofloxacin, taken as the standard antibiotic, was administered as the control drug. Another disc was treated with DMSO as the negative control. A process of overnight incubation of 24 h at a controlled temperature of 37 °C then took place for the MHA plates after sealing them with parafilm. Following the incubation period, the zones of inhibition (ZOIs) surrounding each disc were diligently measured in millimeters (mm) to denote the efficacy of each compound [64,65]. 

#### 5.2.6. Fractional Inhibitory Concentration Index (FICI)

The assessment of FICI, which assessed the potential synergistic action between the standard antibiotic ciprofloxacin and selected compounds **HD6** and **HS8**, was facilitated through the effective application of the microdilution checkerboard procedure. Using a 96-well plate filled with nutrient broth, a horizontal dilution series of ciprofloxacin was prepared, generating concentrations of 16, 8, 4, 2, 1, 0.5, 0.25, 0.125, 0.062, and 0.031 μg/mL. Simultaneously, a top-to-bottom dilution of each compound was devised to yield concentrations of 256, 128, 64, 32, 16, 8, 4, and 2 μg/mL, constituting a wide range of combinations. Subsequently, 15 μL of the bacterial suspension was instilled into each well, with a cell concentration of approximately 2 × 10^5^ CFU/mL, followed by incubation at a maintained 37 °C in a shaking incubator executing 90 rotations per minute. Post-incubation, the extent of bacterial growth was ascertained by capturing an absorbance readout at 590 nm using a spectrophotometer. Wells devoid of visible bacterial growth suggested the effective combined MIC values. The degree of synergism between the tested compounds was evaluated in the context of the FICI (fractional inhibitory concentration index) using the standard formula:

$$\mathrm{FICI}=\frac{MIC\;of\;the\;drug\;A\;in\;combination\;with\;B}{MIC\;of\;the\;drug\;A\;alone}+\frac{MIC\;of\;the\;drug\;B\;in\;combination\;with\;A}{MIC\;of\;the\;drug\;B\;alone}$$

Synergy and antagonism were defined by FICI indices of ≤0.5 and ≥4, respectively. A FICI index result between >0.5 and <4 was considered indifferent [66].

#### 5.2.7. Hemolytic Assay

The hemolytic potential of test compound **HD6** was evaluated following a specific protocol. Human blood was obtained from a healthy individual and collected into a tube with EDTA as an anticoagulant. The erythrocytes were separated by centrifuging the blood at 2000 rpm for 10 min at 20 °C. Following centrifugation, the erythrocytes underwent three washes with phosphate-buffered saline (PBS). The washed cell pellets were then resuspended in PBS to create a 10% erythrocyte suspension. This suspension was further diluted in PBS at a ratio of 1:10. For the assay, 100 microliters of the diluted suspension were transferred into microcentrifuge tubes in triplicate, each containing 100 microliters of different dilutions of the test compound, with matching buffer. To ensure total hemolysis, a final concentration of 1% Triton X-100 was added. The tubes were incubated at 37 °C for 60 min. Post-incubation, centrifugation was performed at 2000 rpm for 10 min at 20 °C. Following this step, 150 microliters of the supernatant was carefully transferred to a flat-bottomed microtiter plate. The absorbance was then measured spectrophotometrically at a wavelength of 450 nm to assess hemolysis [61]. The percentage of RBCs lysed was calculated using the following formula:

$$\%\;\mathrm{Hemolysis}=\frac{\left(OD\;of\;the\;test\;compound\;treated\;sample-OD\;of\;the\;buffer\;treated\;sample\right)}{\left(OD\;of\;the\;1\%\;TritonX-100\;treated\;sample-OD\;of\;the\;buffer\;treated\;sample\right)}-100$$

#### 5.2.8. Zone of Inhibition of Environmental Resistant Strains

A disc diffusion assay was conducted using multidrug-resistant strains that were taken from environmental waterbodies. These were tested against the **HD6** compound as this compound showed promising results in comparison to the other test compounds. The MDR strains were inoculated and incubated overnight at 37 °C on Mueller–Hinton agar. An inoculum (10^5^ CFU/mL) was mixed with molten Mueller–Hinton agar (MHA) and poured into 90 mm petri plates. Once solidified, 4 mm sterile Whatman paper discs were placed on the agar. Ampicillin was used as the positive control, with DMSO as the negative control. After 24 h of incubation at 37 °C, zones of inhibition (ZOIs) were measured to evaluate the antimicrobial efficacy. 

### 5.3. Selection of Target Protein and Preparation of Protein Target Structure

#### 5.3.1. Protein Preparation

We inserted biofilm-associated protein with PDB ID 7C7U and performed protein preparation by utilizing the Protein Preparation Wizard within Schrödinger Maestro 2022-4. To optimize the protein structure’s energy, OPLS4 force field was employed (Schrodinger, LLC, New York, NY, USA, 2009) [67].

#### 5.3.2. Ligand Preparation

The ligand named **HD6** was drawn in 2D sketcher in Schrodinger software. Preparation of the ligand utilized the LigPrep module within Schrodinger Maestro 2022-3 (Schrodinger, LLC, New York, NY, USA, 2009). The 2D structure was converted to a 3D structure, with optimized geometry and adjusted for chirality and desalting. Ionization and tautomeric states were determined using the Epik module, accommodating pH values ranging from 5 to 9. The ligand underwent minimization using the Optimized Potentials for Liquid Simulations-4 (OPLS-4) force field integrated within Schrodinger software [68], and hence, an energetically favored ligand was prepared for docking analysis [69].

#### 5.3.3. Validation of Binding Site

The binding site analysis of the protein is a key feature in molecular docking. All the possible site maps were generated for the protein using the site map tool in Maestro 2022-4 of the Schrödinger suite. 

#### 5.3.4. Grid Box Generation

The receptor grid box of the protein’s active site was generated using the “Glide’s Receptor Grid Generation” module. The ligand was docked with the protein’s selected binding pocket using Glide and OPLS-4 force fields [70]. 

#### 5.3.5. Glide Ligand Docking

Standard precision (SP) and extra precision (XP) docking were performed with the ligand and the protein. The glide score scoring function evaluated hydrogen bonds, hydrophobic interactions, and electrostatic interactions and avoided steric clashes and provided a glide score and a docking score [71,72].

#### 5.3.6. Calculation of Binding Free Energy Using Prime/MM-GBSA Approach

To calculate the binding free energy of the receptor–ligand complexes, the prime module was employed from the Schrödinger suite 2022-4. The Molecular Mechanics–Generalized Born Surface Area (MM-GBSA) method utilizing the OPLS4 force field, the VSGB solvent model, and search algorithms were utilized [73]. 

#### 5.3.7. Molecular Dynamics Simulation

When analyzed in a protein–ligand 2D interaction diagram, the best complex was selected based on the highest number of hydrogen-bond interactions and high binding energy to perform molecular dynamics (MD) simulation. The Desmond module was employed from Schrödinger Release 2022-4, running on a Linux system. MD simulation captures the dynamic stability of every atom of the protein–ligand complex at every point in time, mimicking the living system. The simulation utilized the Optimized Potentials for Liquid Simulations (OPLS4) force field at pH 7.4. To identify the most suitable binding complexes, we conducted a 100 ns simulation. This process involved placing the protein and selected complex in an orthorhombic box and solvating them with water molecules. To balance charges and maintain a salt concentration of 0.15 M, we introduced Na^+^ and Cl^−^ ions. Throughout the simulation, we kept a constant temperature of 300 K and a pressure of 1.01325 bar. Following established protocols, we recorded data at 5 ps intervals [74]. The stability of the ligand–protein complex was assessed by analyzing parameters such as root mean square deviation (RMSD), root mean square fluctuation (RMSF), and the values of secondary structure elements (SSE). Additionally, we examined the interactions between ligand and amino acid residues for each trajectory frame. We also assessed conformation during the 100 ns simulation by evaluating the radius of gyration (Rg) and solvent-accessible surface area (SASA) [75,76,77,78,79,80].

## 6. Conclusions

In summary, a variety of novel 4-aminoquinoline hydrazones and isatin hybrids with appropriate substitutions were synthesized and screened for antibacterial activity against both Gram-positive and Gram-negative strains. Notably, among all derivatives (**HD1-23** and **HS1-12**), compound **HD6** exhibited significant antibacterial efficacy, indicated by an MIC in the range of (8–16 μg/mL) and an MBC of (8–128 μg/mL) against the bacterial strains *E. faecalis*, *B. subtilis*, *S. aureus*, and *P. aeruginosa*. Additionally, the combination of **HD6** with ciprofloxacin resulted in substantial reductions in MIC values of both **HD6** and CIP, showcasing a synergistic interaction with the standard drug ciprofloxacin, particularly against *P. aeruginosa* (FICI = 0.37), which underscores its potential in combination therapy to enhance antibacterial efficacy against resistant infections. Additionally, a hemolytic assay demonstrated the non-toxicity of **HD6**. Furthermore, molecular docking studies revealed strong binding interactions of **HD6** with biofilm-associated protein (PDB ID: 7C7U), providing insights into its potential mechanism of action. The docking analysis indicated favorable binding energy scores, suggesting the compound’s capability to effectively inhibit bacterial growth. Overall, these results signify the promising potential of the synthesized compound **HD6** to be developed as an effective and safe antibacterial agent.

## Figures and Tables

**Table 1:** In vitro preliminary screening showing zones of inhibition at 10 mg/mL (mm).

S. No.	Compound	*E. faecalis* (MTCC439)	*B. subtilis* (MTCC736)	*S. aureus* (MTCC902)	*P. aeruginosa* (2453)
1	HD1	10	-	10	08
2	HD2	-	-	-	10
3	HD3	-	-	-	-
4	HD4	11	09	11	11
5	HD5	10	-	-	10
6	HD6	11	10	11	11
7	HD7	-	-	-	-
8	HD8	-	-	-	-
9	HD9	10	-	-	11
10	HD10	-	-	-	-
11	HD11	10	-	11	11
12	HD12	-	-	-	-
13	HD13	13	-	-	-
14	HD14	-	-	-	-
15	HD15	-	-	-	-
16	HD16	-	-	-	-
17	HD17	11	-	-	10
18	HD18	12	-	-	11
19	HD19	-	-	-	-
20	HD20	-	-	-	-
21	HD21	-	-	-	-
22	HD22	11	-	-	12
23	HD23	-	-	-	-
24	HS1	-	-	-	-
25	HS2	6	-	6	6
26	HS3	-	-	-	-
27	HS4	6	-	-	-
28	HS5	-	-	-	-
29	HS6	-	-	-	-
30	HS7	6	6	6	-
31	HS8	-	7	9	6
32	HS9	-	-	-	-
33	HS10	6	-	-	-
34	HS11	-	-	-	-
35	HS12	-	-	-	-
36	Cip *	12	13	15	16
37	DMSO	-	-	-	-
38	Blank	-	-	-	-

* Cip: ciprofloxacin.

**Table 2:** Minimum inhibitory concentration (MIC) of compounds in µg/mL.

S. No.	Compound	*E. faecalis*	*B. subtilis*	*S. aureus*	*P. aeruginosa*
1	HD1	512	>1024	>1024	>1024
2	HD4	512	512	1024	>1024
3	HD6	128	8	128	16
4	HD11	1024	1024	>1024	1024
5	HS2	1024	>1024	>1024	>1024
6	HS7	>1024	>1024	>1024	>1024
7	HS8	256	256	1024	>1024
8	CIP *	0.5	1.0	0.125	0.5

* CIP: ciprofloxacin.

**Table 3:** Zone of inhibition (in mm) measured around the disc of ½ MIC, MIC, and 2MIC concentrations of the compounds **HD6** and **HS8**.

Bacterial Strain	Zone of Inhibition at Different Concentrations (mm)					
	HD6			HS8		
	½ MIC	MIC	2MIC	½ MIC	MIC	2MIC
*E. faecalis*	6	7	10	6	6	11
*B. subtilis*	8	10	10	6	6	6
*S. aureus*	11	11	12	6	6	8
*P. aeruginosa*	9	10	11	6	6	6

**Table 4:** Synergistic effect of compounds **HD6** and **HS8** with standard antibacterial drug ciprofloxacin (CIP).

	Bacterial Strain	MIC Alone			MIC in Combination			FICI	Mode of Interaction
		HD6	HS8	CIP	HD6	HS8	CIP		
	*E. faecalis*	128	-	0.5	2	-	0.25	0.515	Synergistic
HD 6	*B. subtilis*	8	-	1	2	-	0.25	0.5	Synergistic
	*S. aureus*	128	-	0.125	2	-	2.015	2.015	Indifferent
	*P. aeruginosa*	16	-	0.5	2	-	0.125	0.375	Synergistic
	*E. faecalis*	-	256	0.5	-	2	0.25	0.50	Synergistic
HS8	*B. subtilis*	-	256	1	-	64	1	1.25	Indifferent
	*S. aureus*	-	1024	0.125	-	2	0.25	2.001	Indifferent

## Data Availability

Data has been provided in the Supplementary Materials.

## Supplementary Materials

The following supporting information can be downloaded at: https://www.mdpi.com/article/10.3390/molecules29235777/s1, Table S1: Physicochemical properties of **HD1-23** and **HS1-12**; Table S2: Minimum bactericidal concentration (MBC) of compounds in µg/mL and MBC-to-MIC ratio (R); Table S3: Zone of inhibition against the MDR strains and spectral data of all the synthesized compounds; Table S4: Zone of inhibition against MDR strains.
