# Supplementary material for: Synthesis and Structure–Activity Relationship (SAR) Studies on New 4-Aminoquinoline-Hydrazones and Isatin Hybrids as Promising Antibacterial Agents
Source: Molecules. 2024 Dec 6;29(23):5777. doi: 10.3390/molecules29235777 (PMC11643852; doi:10.3390/molecules29235777)
Supplement: Supplementary file 1 [file molecules-29-05777-s001.zip › molecules-3296604-supplementary.pdf]

Synthesis and Structure–Activity Relationship (SAR) Studies on New 4-Aminoquinoline-Hydrazones and Isatin Hybrids as Promising Antibacterial Agents

Ayesha Ubaid[a], Mohd Shakir[a], Asghar Ali[a, b], Sobia Khan[a], Jihad Alrehaili[c], Razique Anwer[c*], Mohammad Abid[a*]

[a] *Department of Biosciences, Jamia Millia Islamia, Jamia Nagar-110025, New Delhi, India*

[b] *Clinical Biochemistry Laboratory, Department of Biochemistry, School of Chemical and Life Science, Jamia Hamdard, New Delhi-110062, India.*

[c]*Department of Pathology, College of Medicine, Imam Mohammad Ibn Saud Islamic University (IMSIU), Riyadh 13317-4233, Saudi Arabia.*

Corresponding Authors: mabid@jmi.ac.in (MA); razainuddin@imamu.edu.sa (RA).

TABLE OF CONTENTS

	Contents	Page No.
1.	Physicochemical properties Table S1	S3-S4
2.	Minimum bactericidal concentration (MBC) of compounds in µg/ml and MBC to MIC ratio (R) Table S2	S5
3.	Zone of inhibition against the MDR strains Table S3	S6
4.	Zone of inhibition against Environmental *E. coli* MDR strains.	S7
5.	Spectral data of the synthesized compounds	S8-S53

Physiochemical Properties

Table S1: Physicochemical properties of HD1-23 and HS1-12

Compound ID	Mol. Weight	SASA	QPPCaco	HBA	HBD	PSA	QPlogBB	Percent Human Oral Absorption	Lipinski Rule Of Five violations	#metab	QPlogHERG
HD1	416.866	744.993	378.089	4.75	3	94.858	7.094	100	0	3	-0.657
HD2	400.866	732.021	1247.104	4	2	72.281	-0.69	100	1	2	-7.886
HD3	435.312	754.497	1187.22	4	2	72.806	-0.58	100	1	2	-7.794
HD4	435.312	756	1247.422	4	2	72.276	-0.535	100	1	2	-7.773
HD5	418.857	740.85	1249.211	4	2	72.225	-0.582	100	1	2	-7.749
HD6	468.865	784.156	1246.896	4	2	72.274	-0.443	100	1	2	-7.801
HD7	445.864	767.366	179.551	5	2	115.587	-0.443	96.073	0	3	-7.795
HD8	445.864	771.02	148.712	5	2	117.189	-1.797	94.359	0	3	-7.779
HD9	445.864	770.999	148.861	5	2	117.299	-1.907	94.365	0	3	-7.78
HD10	430.893	770.482	1247.237	4.75	2	80.467	-1.907	100	1	3	-7.77
HD11	460.919	809.292	1246.237	5.5	2	87.901	-0.776	100	1	4	-7.666
HD12	479.763	762.075	1227.564	4	2	72.279	-0.865	100	1	2	-7.812
HD13	416.866	744.39	378.692	4.75	3	94.805	-0.536	100	0	3	-7.743
HD14	443.935	810.834	1173.655	5	2	75.964	-1.381	100	1	3	-7.835
HD15	442.947	819.828	1246.386	4	2	72.269	-0.841	100	1	3	-7.856
HD16	439.903	771.515	658.075	4	3	86.622	-0.813	100	1	2	-8.078
HD17	414.893	764.474	1246.982	4	2	72.279	-1.048	100	1	3	-7.77
HD18	446.892	786.592	375.153	5.5	3	102.187	-0.71	100	0	4	-7.691

							7				
HD19	406.889	717.8	1225.298	4	2	72.713	-1.5	100	1	3	-7.521
HD20	390.828	694.995	1192.764	4.5	2	81.633	-0.572	100	0	3	-7.475
HD21	401.854	728.155	790.836	5	2	84.481	-0.682	100	0	3	-7.767
HD22	389.843	707.905	696.863	4	3	86.409	-0.925	100	0	2	-7.543
HD23	451.914	799.696	904.884	5	2	83.866	-0.973	100	1	2	-8.416
HS1	441.876	748.122	230.258	5	2	114.397	-0.912	100	0	2	-7.632
HS2	455.902	790.744	230.573	5	2	113.676	-1.491	86.856	1	3	-7.675
HS3	486.873	802.174	25.278	6	2	156.685	-1.576	76.793	0	3	-7.744
HS4	455.902	780.429	230.425	5	2	114.388	-2.857	86.539	1	3	-7.517
HS5	476.321	773.36	230.404	5	2	114.388	6.938	87.657	1	2	-7.54
HS6	476.321	757.525	325.613	5	2	113.866	-1.356	90.389	1	2	-7.395
HS7	520.772	772.497	273.665	5	2	114.384	-1.137	76.581	2	2	-7.519
HS8	471.902	785.477	230.303	5.75	2	122.598	-1.243	100	0	3	-7.507
HS9	476.321	772.225	230.254	5	2	114.395	-1.595	87.603	1	2	-7.524
HS10	469.929	824.516	223.862	5	2	113.654	-1.353	88.449	1	4	-7.561
HS11	459.866	757.106	230.241	5	2	114.399	-1.643	86.089	1	2	-7.498
HS12	471.902	788.394	223.347	5.75	2	122.601	-1.391	100	0	3	-7.542

Table S2: Minimum bactericidal concentration (MBC) of compounds in µg/ml and MBC to MIC ratio (R)

S. No.	*Compound*	*E. faecalis*		*B. subtilis*		*S. aureus*		*P. aeruginosa*	
		**MBC**	**R***	**MBC**	**R***	**MBC**	**R***	**MBC**	**R***
1	**HD 1**	512	1	ND	-	ND	-	ND	-
2	**HD 4**	512	1	512	1	>1024	>2	ND	-
3	**HD 6**	256	2	8	1	128	1	16	1
4	**HD 11**	>1024	>2	>1024	>2	ND	-	>1024	>2
5	**HS-2**	>1024	>2	ND	-	ND	-	ND	-
6	**HS-7**	ND	-	ND	-	ND	-	ND	-
7	**HS-8**	>1024	>2	>1024	>2	>1024	>2	ND	-

R* : Represents the MBC/MIC Ratio

ND: Not determined

Table S3: Zone of inhibition against the MDR strains

MDR strains	HD-6	Ampicillin	Resistance pattern	Number of antibiotics found insensitive against the isolates
AA 201	-	-	MET, AMP, PI, CZ, CXM, CTX, CPM, IPM, MRP, CIP, Na, NX, AZM, GEN, VA, NIT, COT, C, CL	19
AA 202	-	-	MET, AMP, PI, CZ, CXM, CTX, CPM, IPM, MRP, CIP, Na, NX, AZM, GEN, VA, NIT, COT, C, CL	19
AA 209	-	30	MET, AMP, CZ, CXM, CTX, CPM, IPM, MRP, Na, NX, AZM, VA, NIT, CL	15
AA 216	-	-	MET, AMP, Pi, CZ, CXM, CTX, CPM, IPM, MRP, CIP, Na, NX, AZM, GEN, VA, NIT, COT, C, CL	19
AA 221	-	-	ME, AMP, Pi, CZ, CXM, CTX, CPM, IPM, MRP, CIP, Na, NX, AZM, VA, NIT, COT, C	17
AA 224	-	-	Met, Amp, Pi, CZ, CXM, CTX, CPM, IPM, MRP, CIP, Na, NX, AZM, GEN, VA, NIT, COT, C, CL	19
AA 237	11	26	Met, Amp, CZ, CTX, IPM, MRP, CIP, Na, NX, AZM, VA,<br>NIT, COT, C, CL	15
AA 240	-	23	Met, Amp, Pi, CZ, CXM, CPM, CIP, Na, AZM, GEN, VA, NIT, COT, C, CL	15
AA 243	-	20	MET, AMP, CZ, CXM, CTX, CPM, Na, NX, AT, AZM, VA, NIT, CL	13
AA 245	-	18	MET, AMP, PI, CZ, CXM, CTX, CPM, IPM, MRP<br>AT, GEN, VA, NIT, CL	14
AA 248	-	25	MET, AMP, CPM, IPM, CIP, Na, NX, AZM, VA, COT, C, CL	13
AA 261	-	25	MET, AMP, CZ, CXM, Na, NIT, COT, C, CL	10
AA 269	-	-	MET, AMP, PI, CZ, CXM, CTX, CPM, IPM, Na, AT, VA, NIT, C, CL	14
AA 273	-	15	MET, AMP, PI, CZ, CXM, CTX, CPM, IPM, MRP, CIP, Na, NX, AZM, GEN, VA, NIT, COT, CL	18
AA 276	-	22	MET, AMP, PI, CZ, CXM, CTX, CPM, MRP, CIP, Na, NX, AZM, GEN, NIT, C, CL	16
AA 290	-	-	MET, AMP, CZ, CXM, Na, NX, VA, NIT, C, CL	10

MET: Methicillin, **AMP:** Ampicillin, **PI:** Piperacillin **CZ:** Ceftazidime, **CXM:** Cefuroxime, **CTX:** Cefotaxime, **CPM:** Cefpodoxime, IPM: Imipenem, MRP: Meropenem, CIP: Ciprofloxacin, NA: Nalidixic acid, NX: Norfloxacin, **AZM:** Azithromycin, **GEN:** Gentamicin, **VA:** Vancomycin, **NIT:** Nitrofuran, **COT:** Co-trimoxazole, **C:** Chloramphenicol, **CL:** Colistin, **AT:** Aztreonam

Table S4: Zone of inhibition against MDR strains.

Isolate	HD6	Ampicillin	Resistance pattern
ECJH1	-	-	AMP, COT, NA
ECJH2	-	30*	TET
ECJH4	-	-	AMP, COT, TET, CIP
ECJH5	-	20	NA, CTX, CAZ, CZ, CP, AZ
ECJH7	-	34	C, NIT, NA, NOR
ECJH11	-	21	TET, NIT
ECJH12	-	-	AMP, CTX
ECJH13	-	-	AMP, CIP, TET, CAZ, NIT, NOR, IPM, CTX
ECJH14	-	-	AMP, COT, CTX
ECJH15	-	20	COT, TET, CTX, CIP, IPM, CAZ
ECJH18	-	17	TET, NIT
ECJH19	-	17	TET, NA, CTX, IPM
ECJH20	-	20	AMP, TET, NIT, NA
ECJH21	-	18	IPM
ECJH22	-	32*	AMP, CIP, NA, TET, NIT, IPM
ECJH23	-	20	NIT, NA, CTX, NOR, C, CAZ, IPM
ECJH24	-	23	NA
ECJH25	-	22	TET, NIT

AMP: Ampicillin, **CZ:** Ceftazidime, **CTX:** Cefotaxime, **IPM:** Imipenem, **CIP:** Ciprofloxacin, **NA:** Nalidixic acid, **NX:** Norfloxacin, **NIT:** Nitrofuran, **COT:** Co-trimoxazole, **C:** Chloramphenicol, **TET:** Tetracycline, **CAZ:** Ceftazidime, **AZ:** Aztreonam, **NOR:** Norfloxacin, **CP:** Carbapenem.

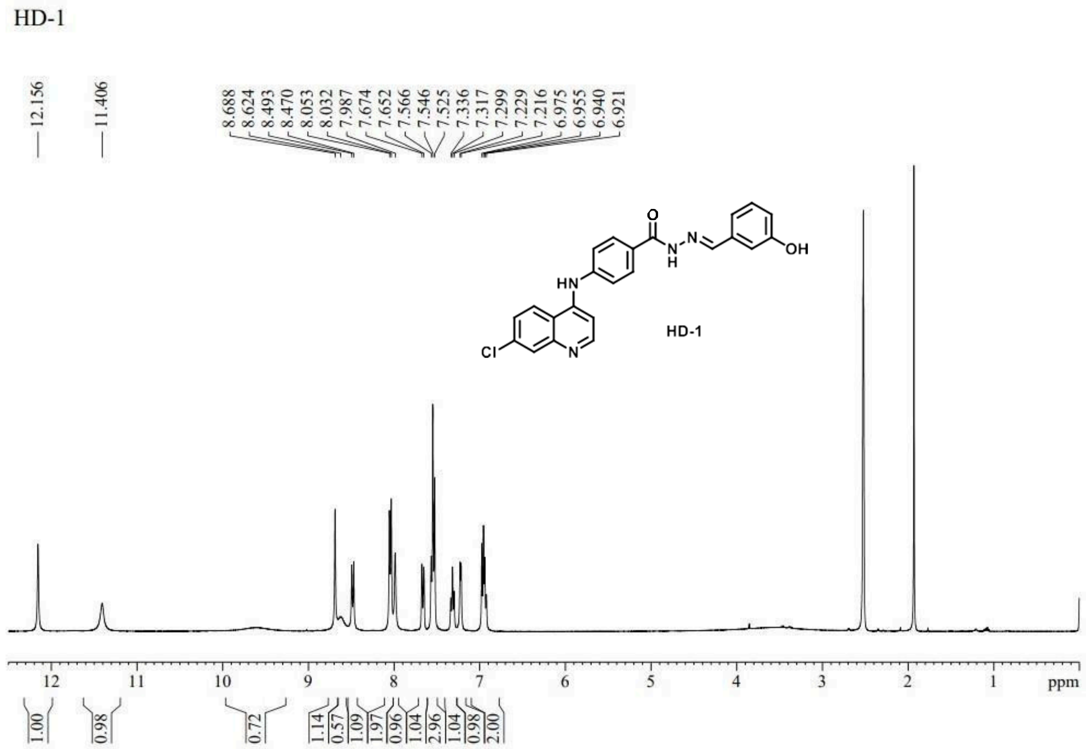

Figure S1: ^{1}H NMR spectrum of **HD1.**

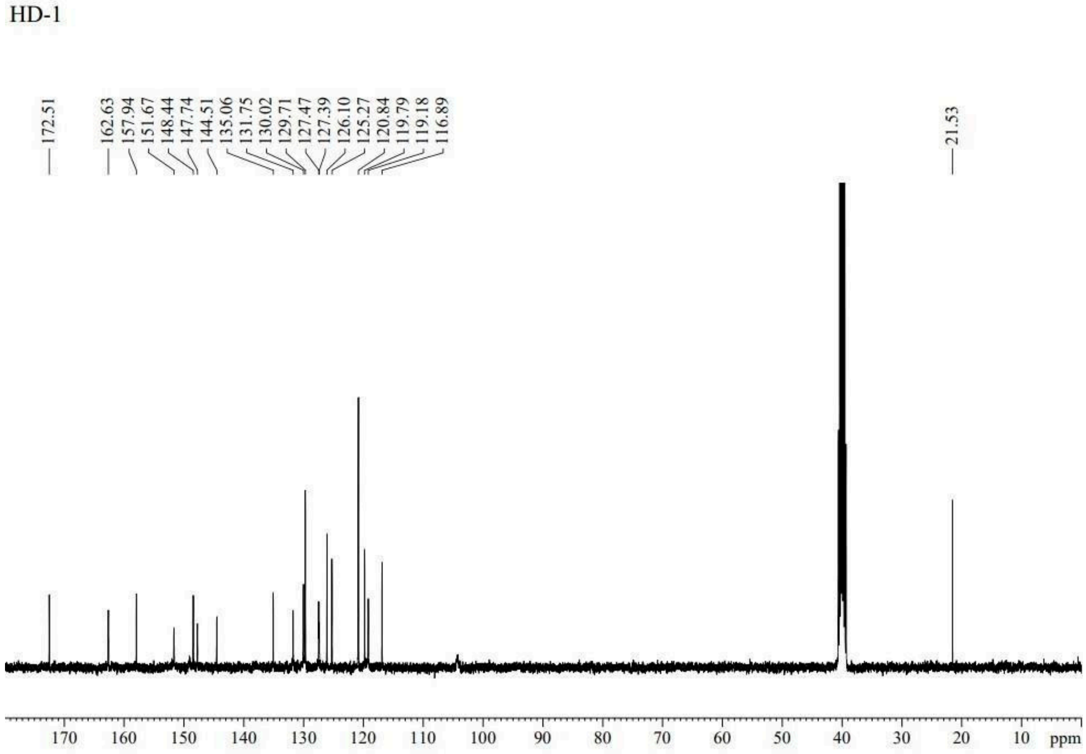

Figure S2: ^{13}C NMR spectrum of **HD1.**

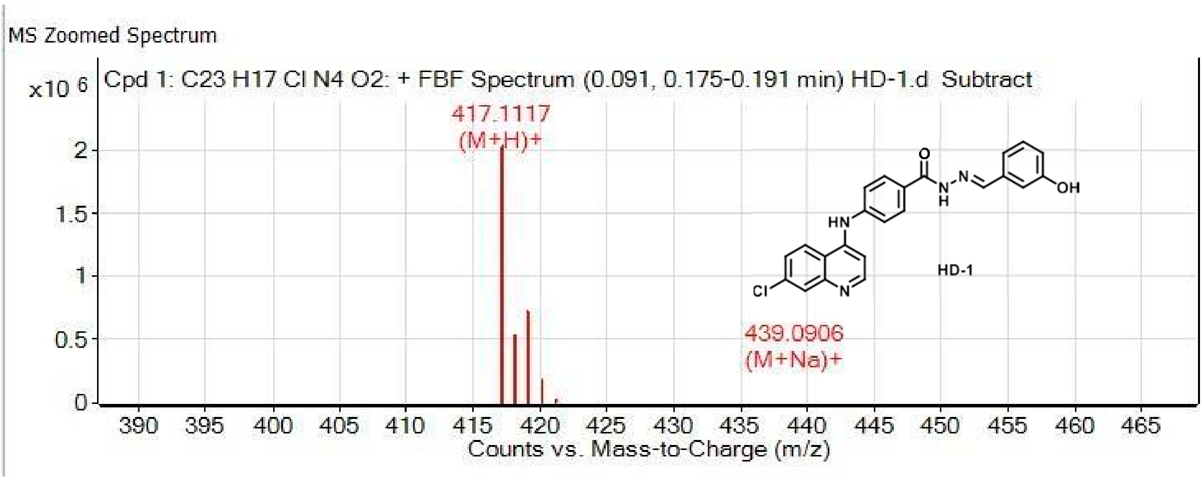

Figure S3: Mass spectrum of **HD1.**

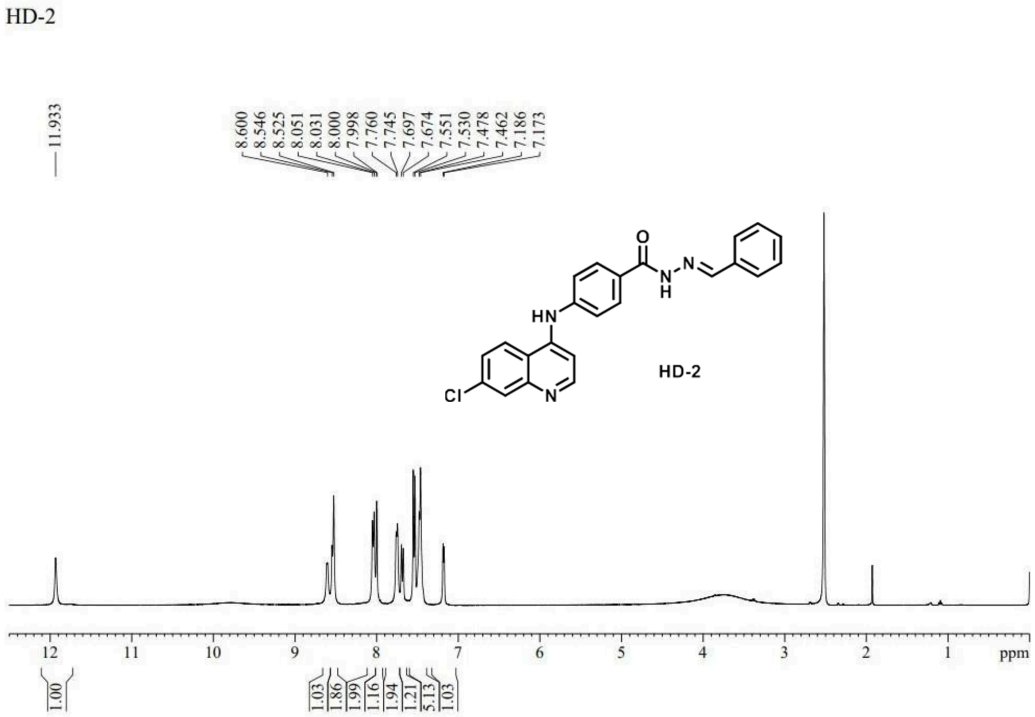

Figure S4: ^{1}H NMR spectrum of **HD2.**

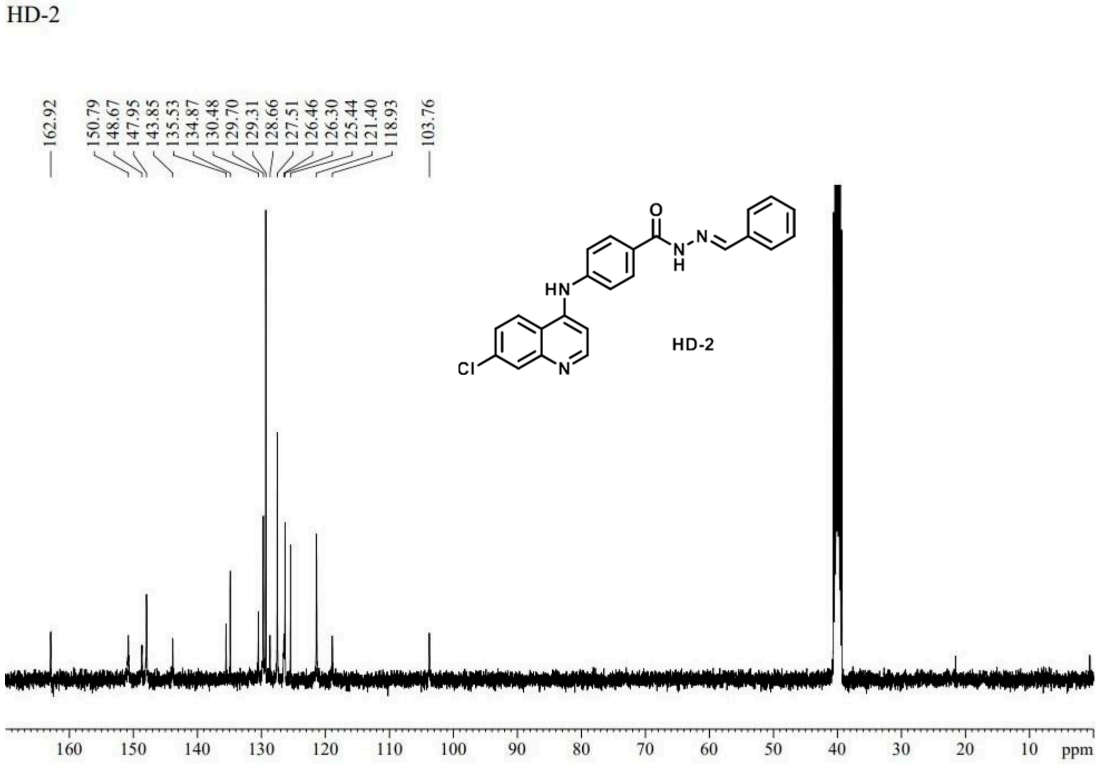

Figure S5: ^{13}C NMR spectrum of **HD2.**

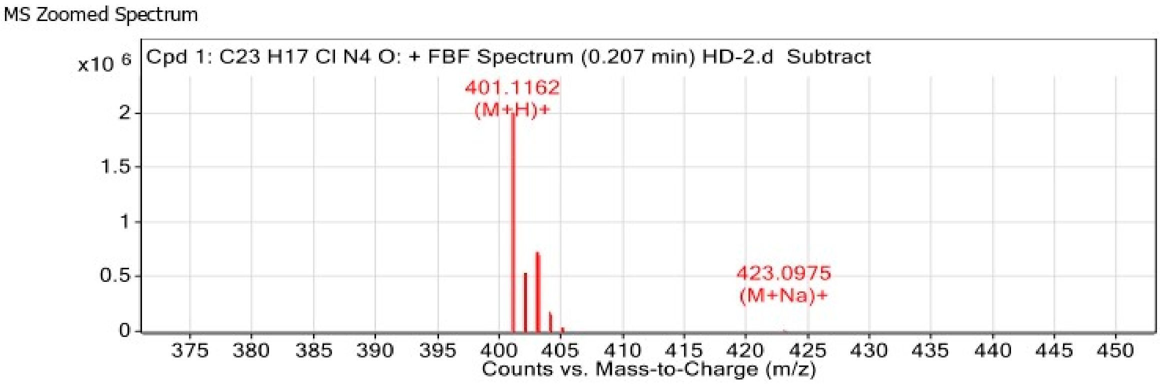

Figure S6: Mass spectrum of **HD2.**

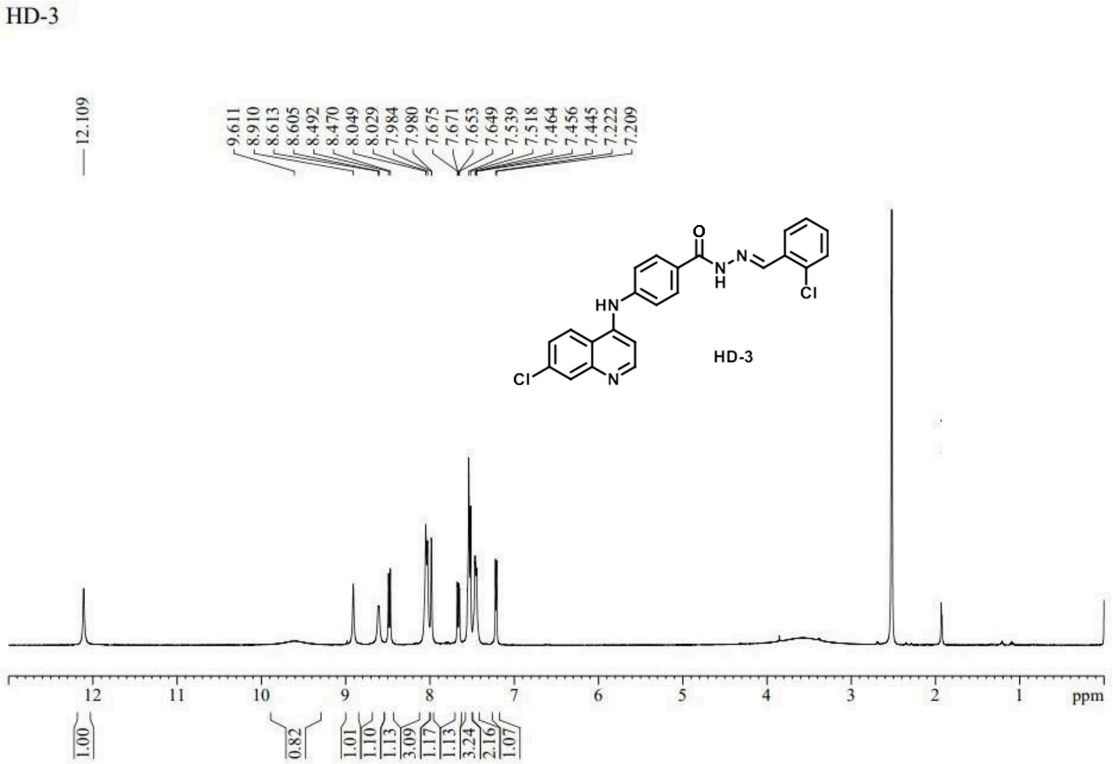

Figure S7: ^{1}H NMR spectrum of **HD3.**

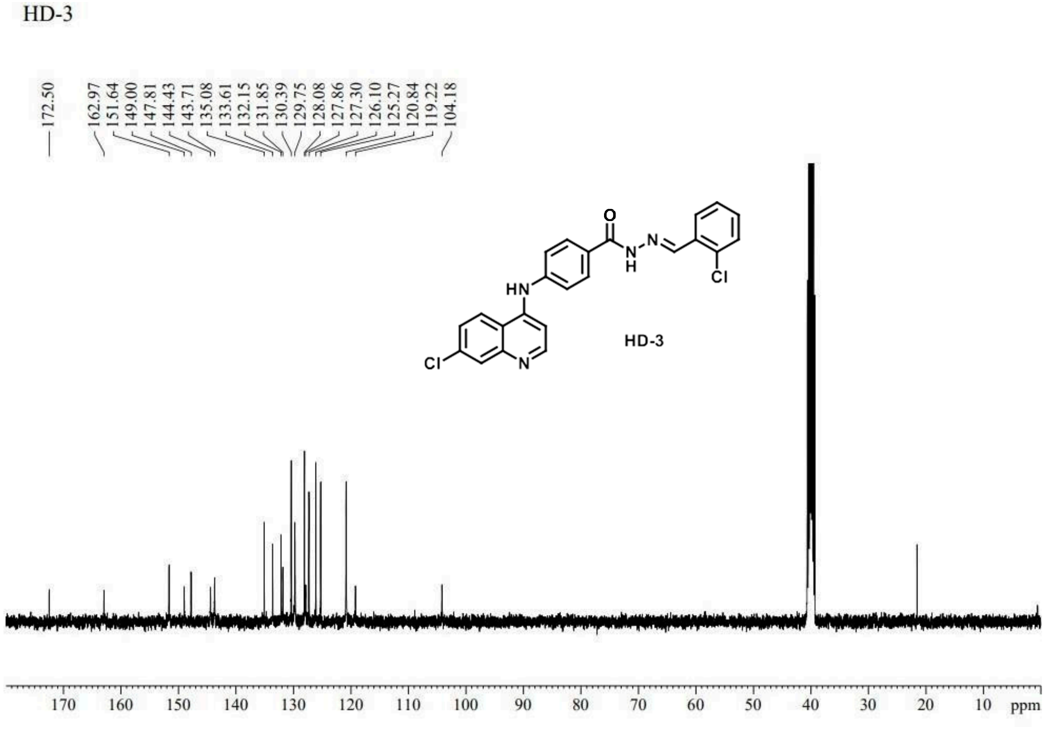

Figure S8: ^{13}C NMR spectrum of **HD3.**

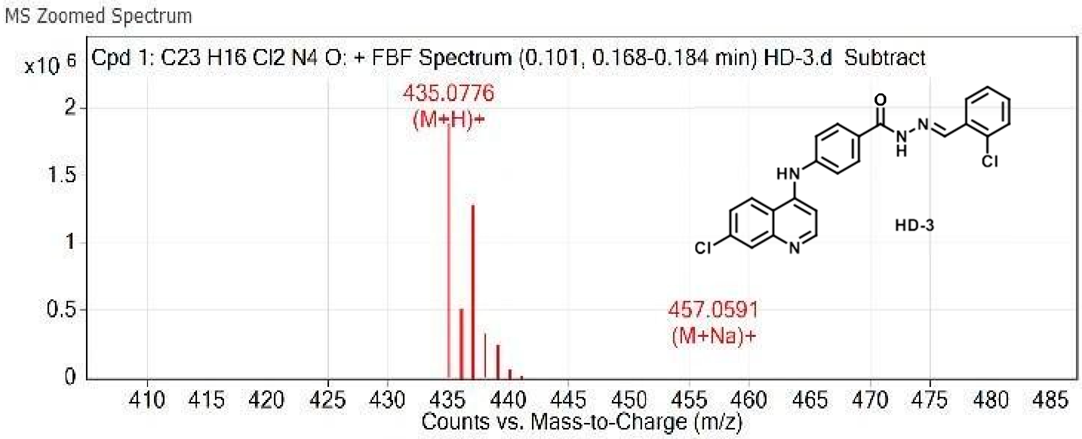

Figure S9: Mass spectrum of **HD3.**

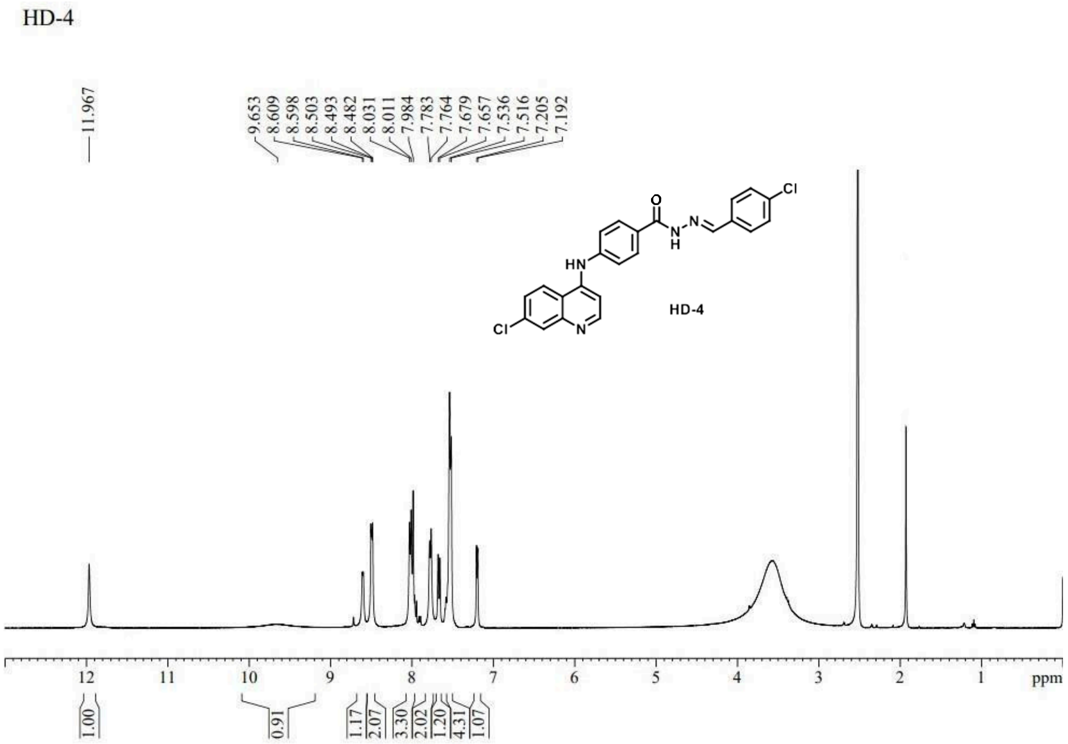

Figure S10: ^{1}H NMR spectrum of **HD4.**

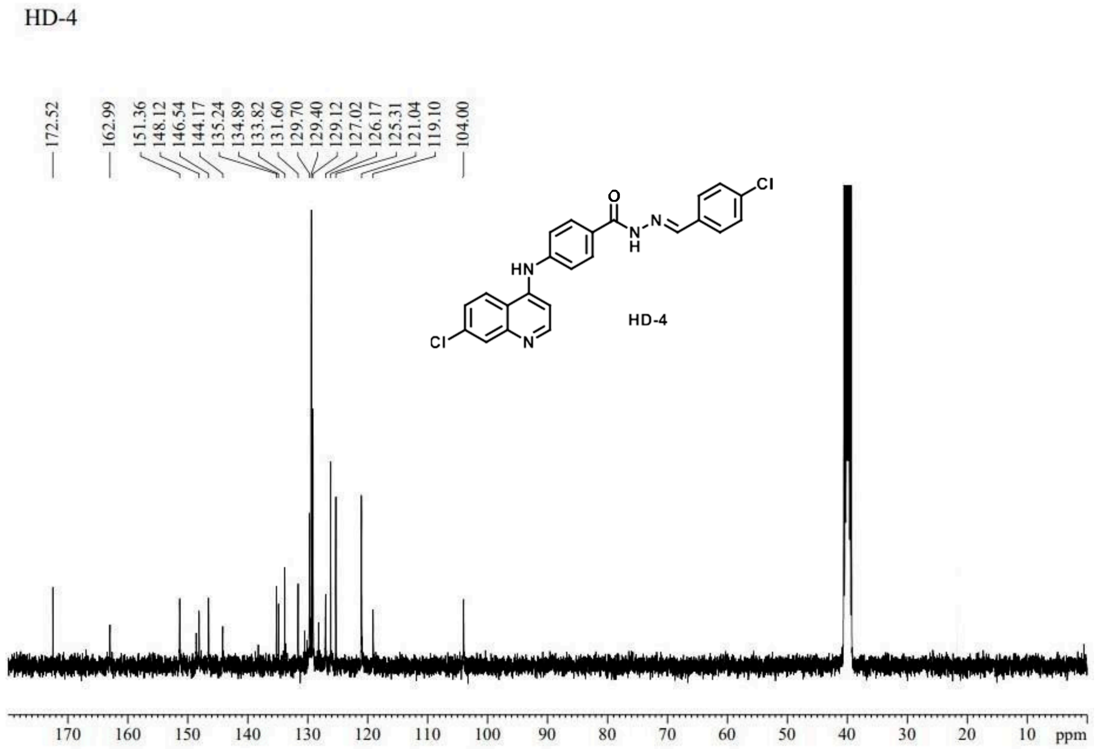

Figure S11: ^{13}C NMR spectrum of **HD4.**

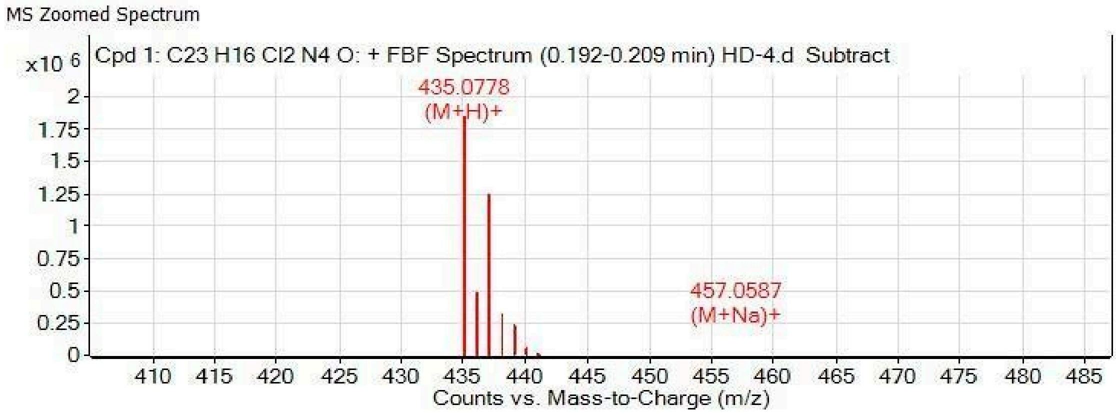

Figure S12: Mass spectrum of **HD4.**

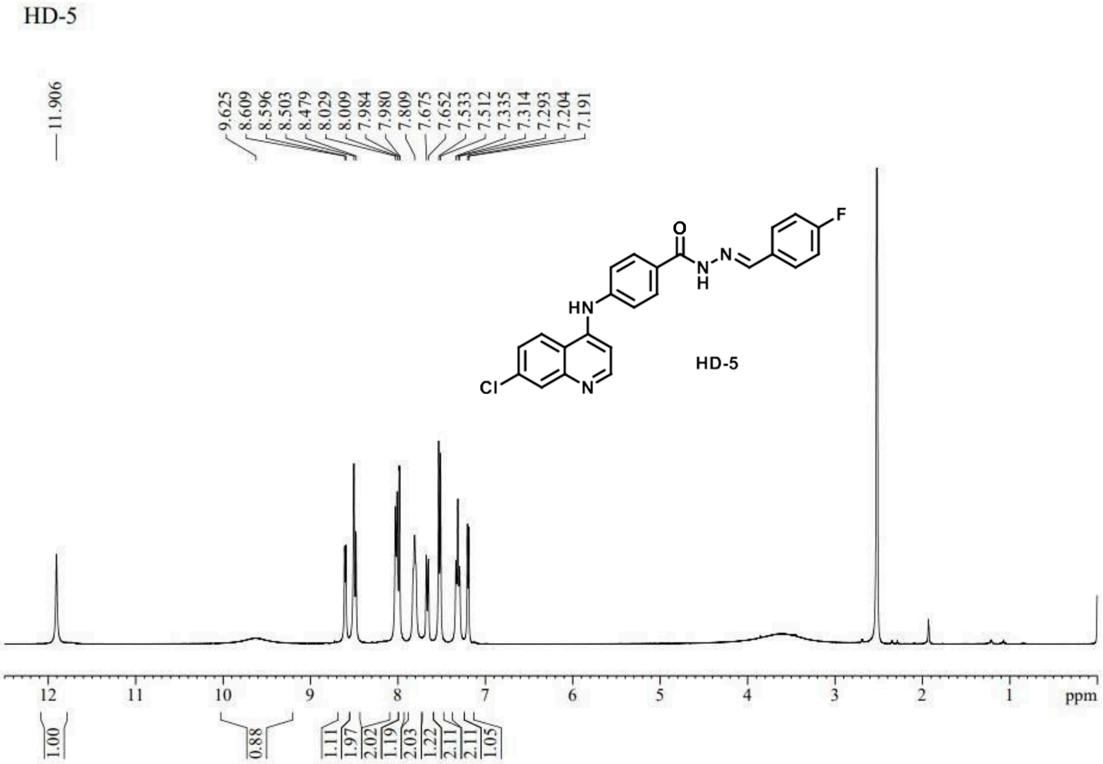

Figure S13: ^{1}H NMR spectrum of **HD5.**

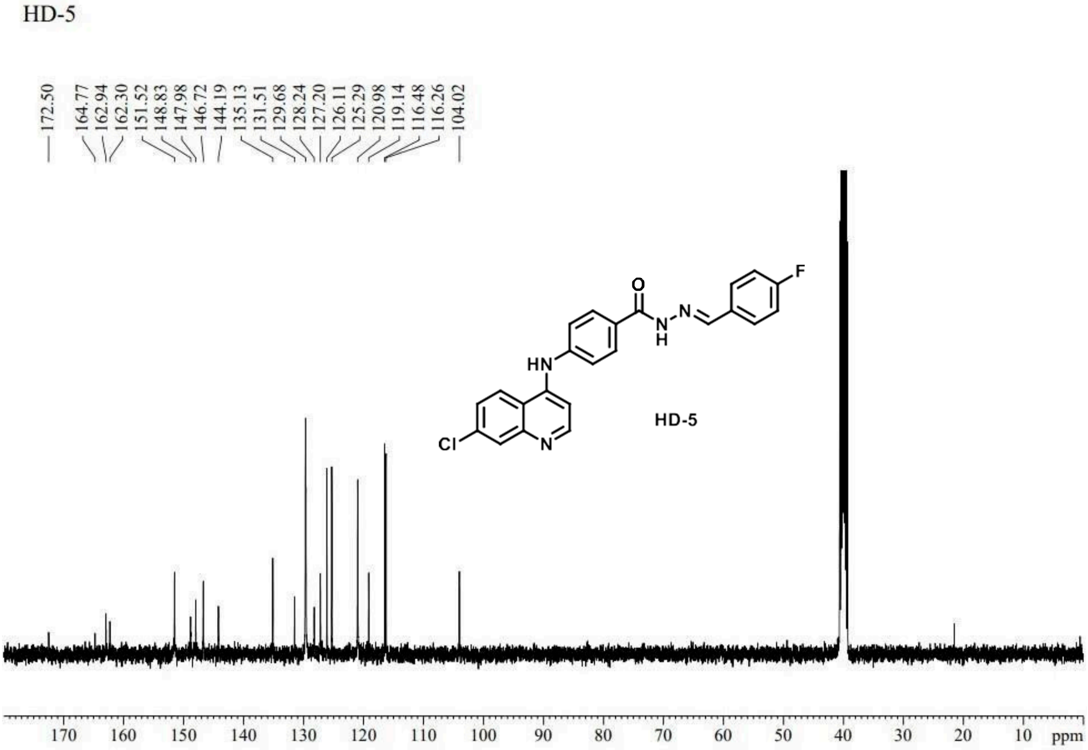

Figure S14: ^{13}C NMR spectrum of **HD5.**

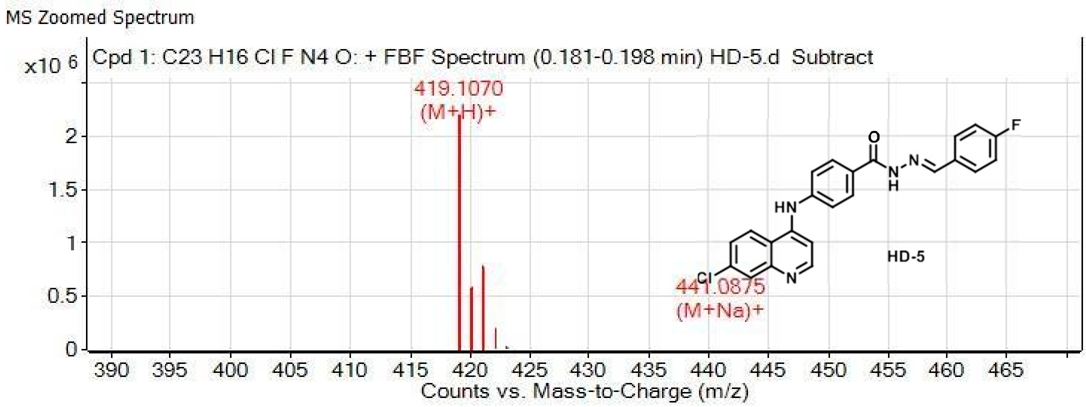

Figure S15: Mass spectrum of **HD5.**

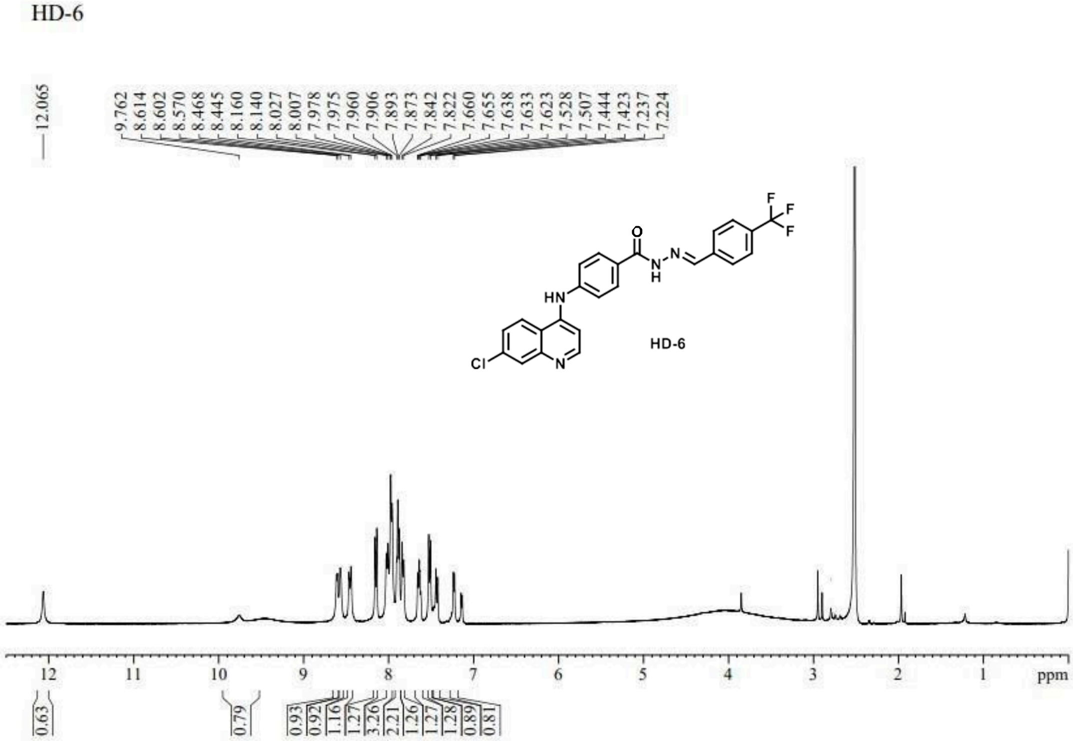

Figure S16: ^{1}H NMR spectrum of **HD6.**

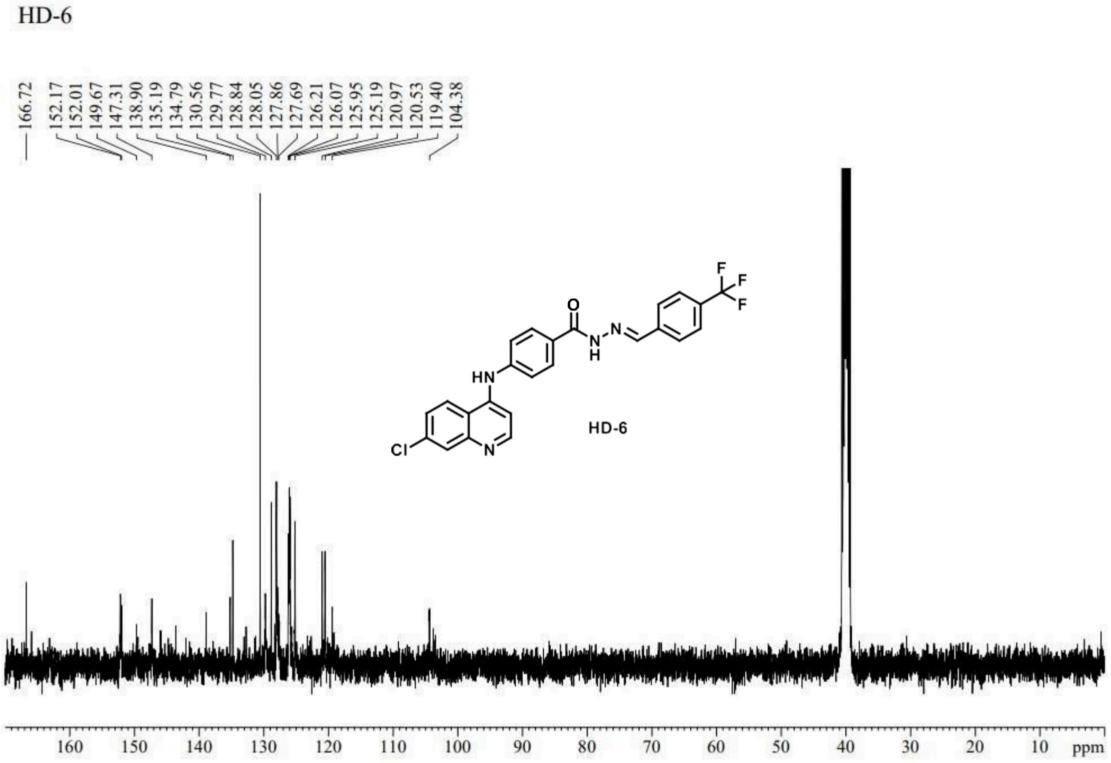

Figure S17: ^{13}C NMR spectrum of **HD6.**

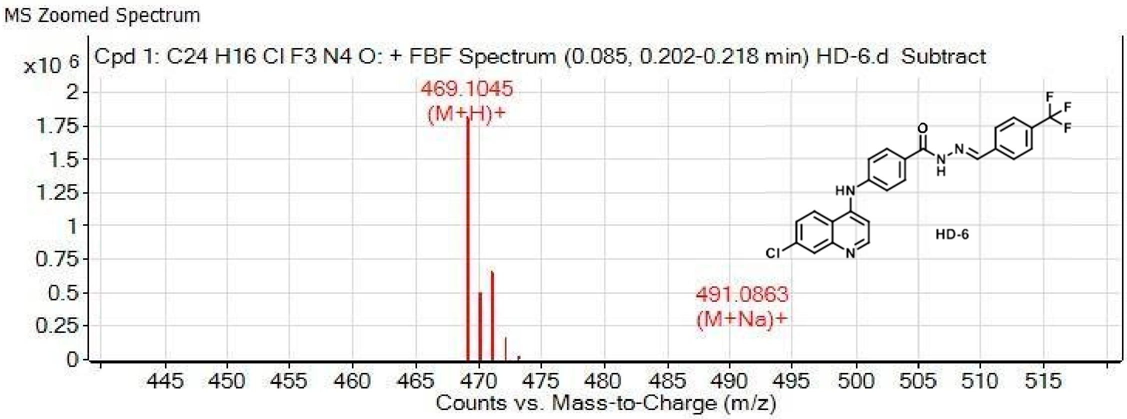

Figure S18: Mass spectrum of **HD6.**

HD-7

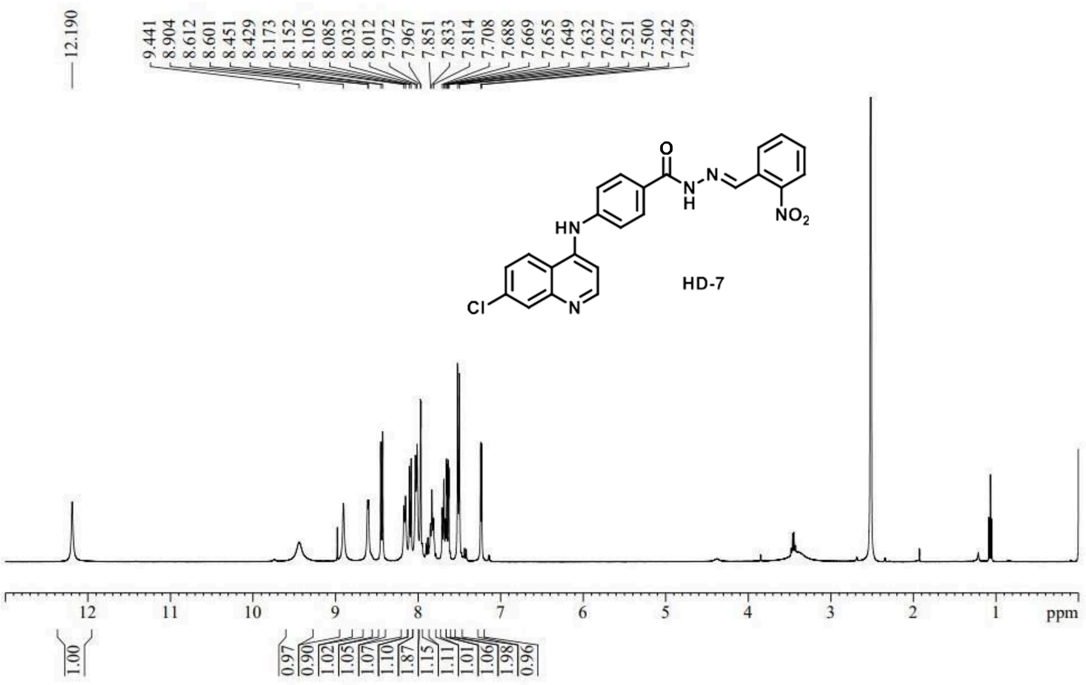

Figure S19: ^{1}H NMR spectrum of **HD7.**

HD-7

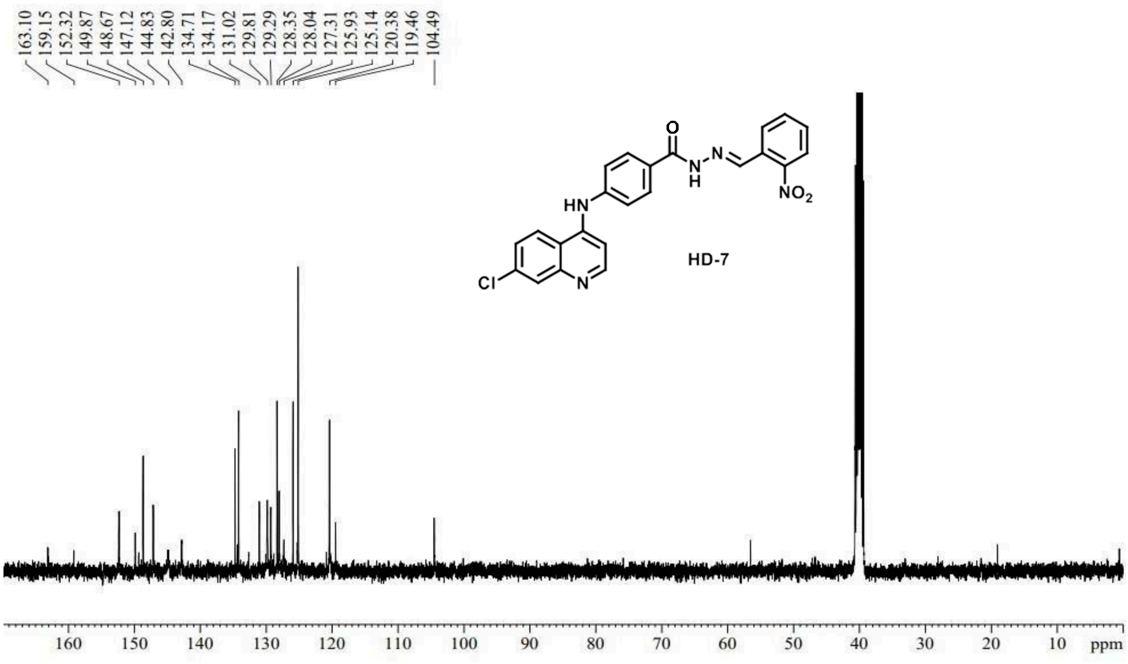

Figure S20: ^{13}C NMR spectrum of **HD7.**

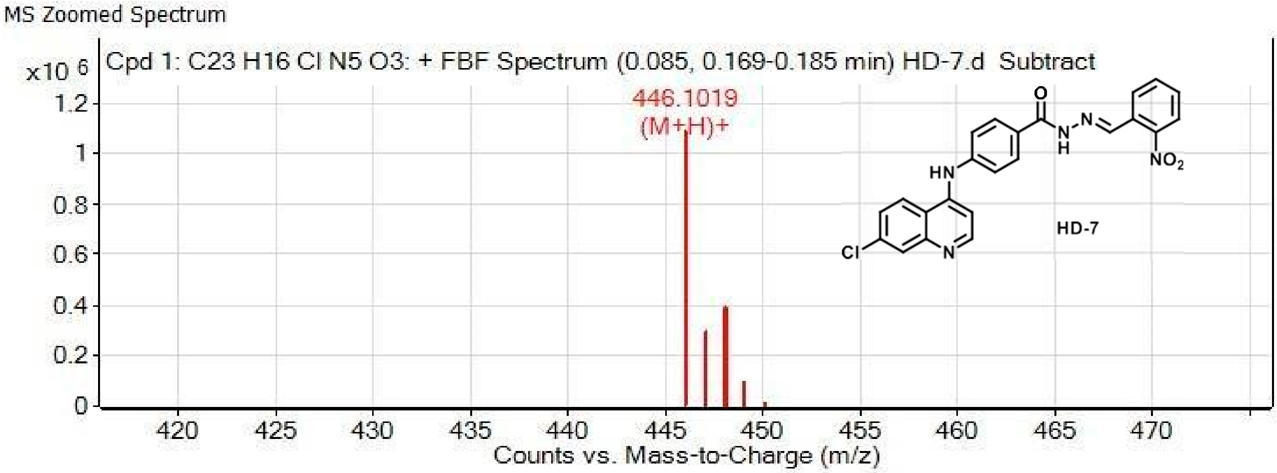

Figure S21: Mass spectrum of **HD7**.

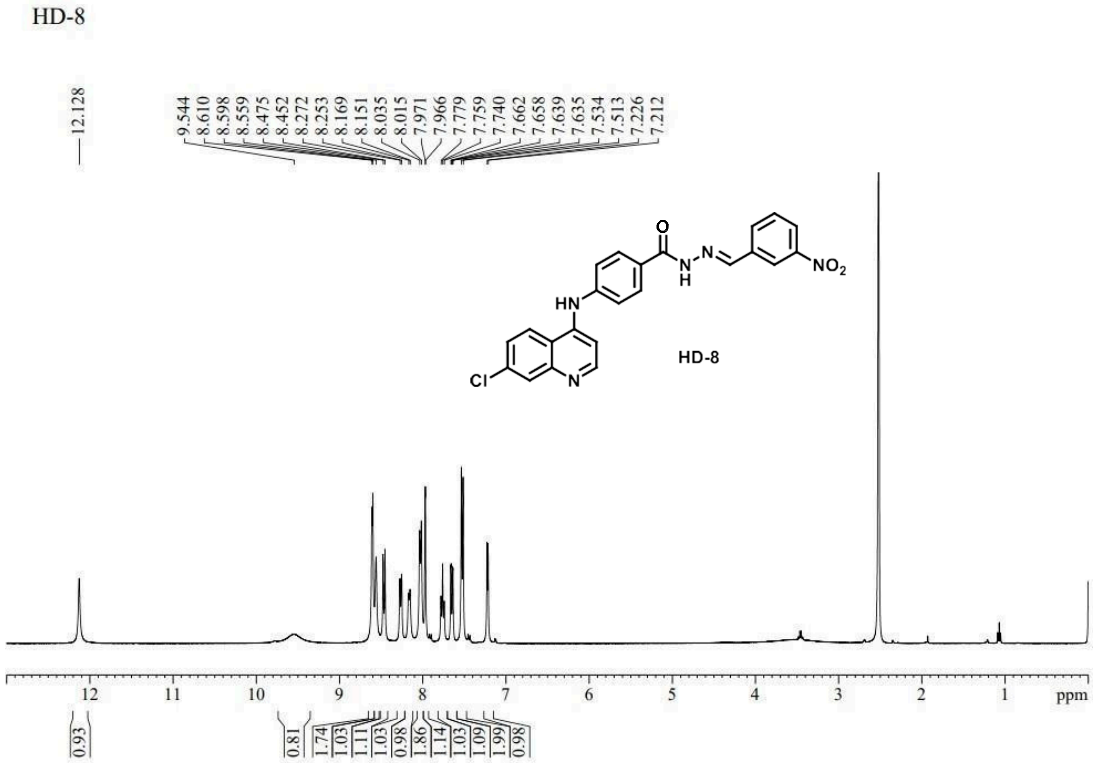

Figure S22: ^{1}H NMR spectrum of **HD8.**

HD-8

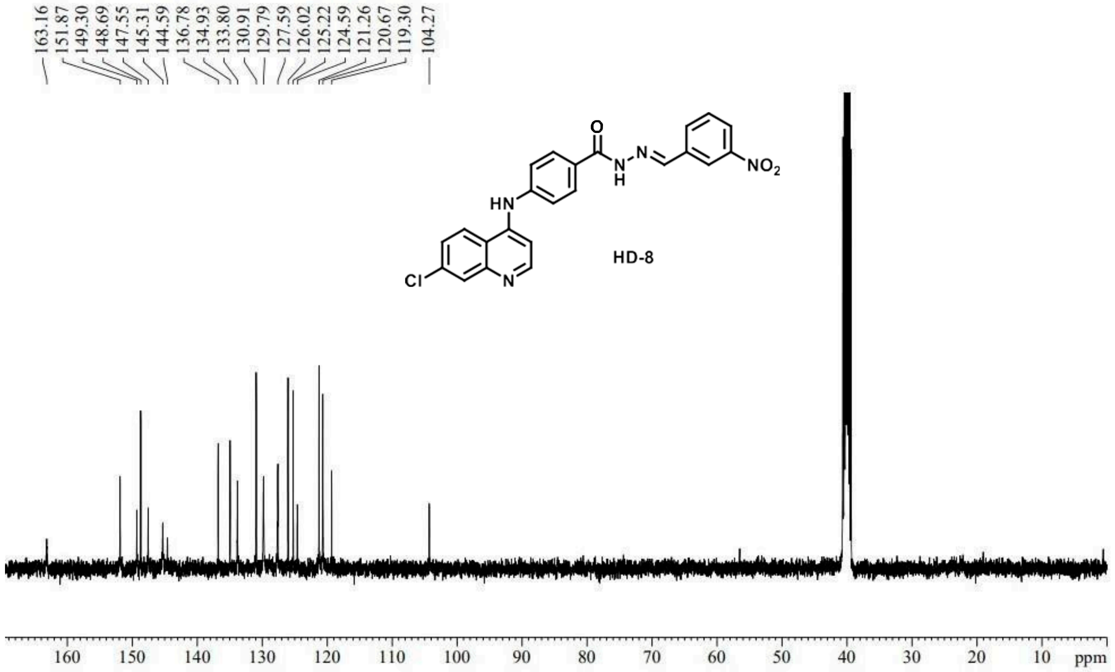

Figure S23: ^{13}C NMR spectrum of **HD8.**

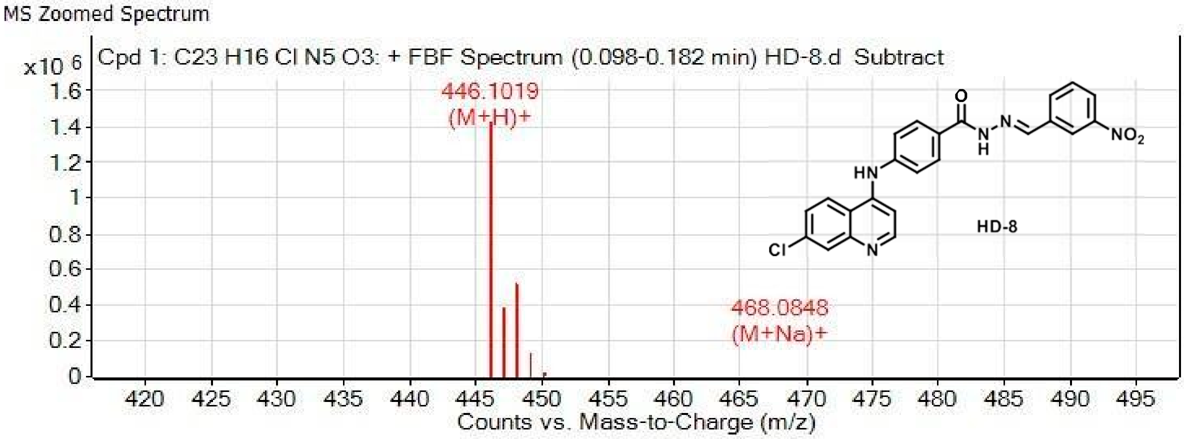

Figure S24: Mass spectrum of **HD8**.

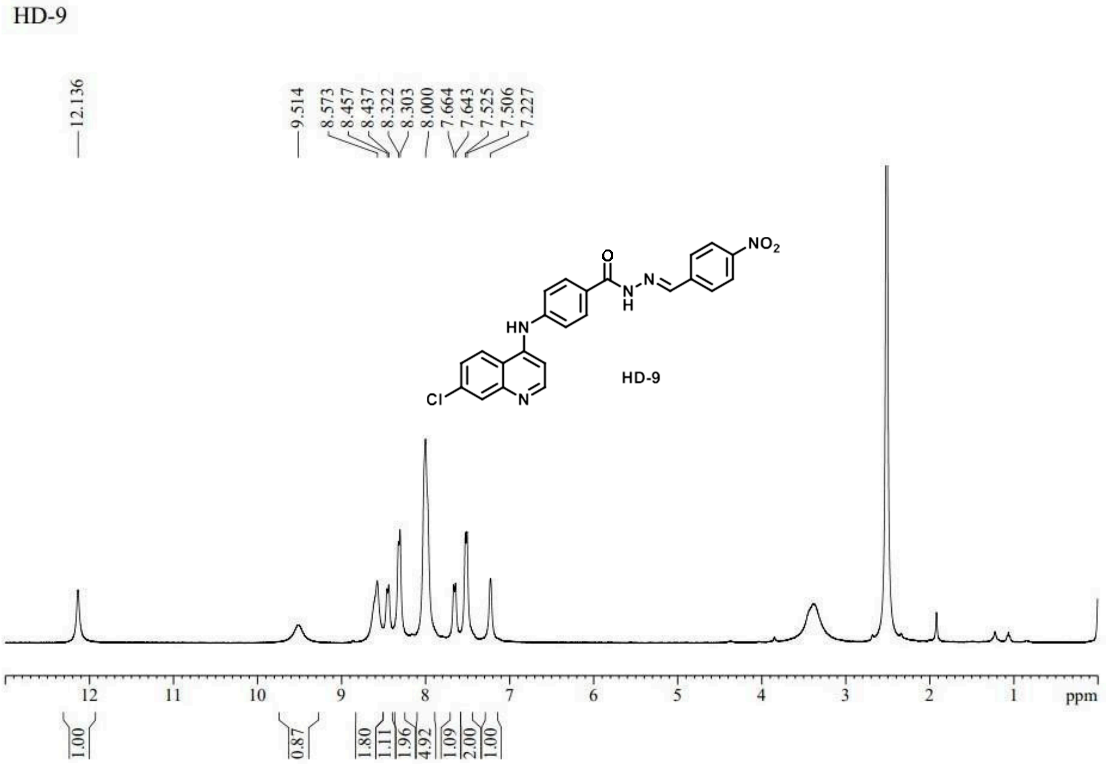

Figure S25: ^{1}H NMR spectrum of **HD9.**

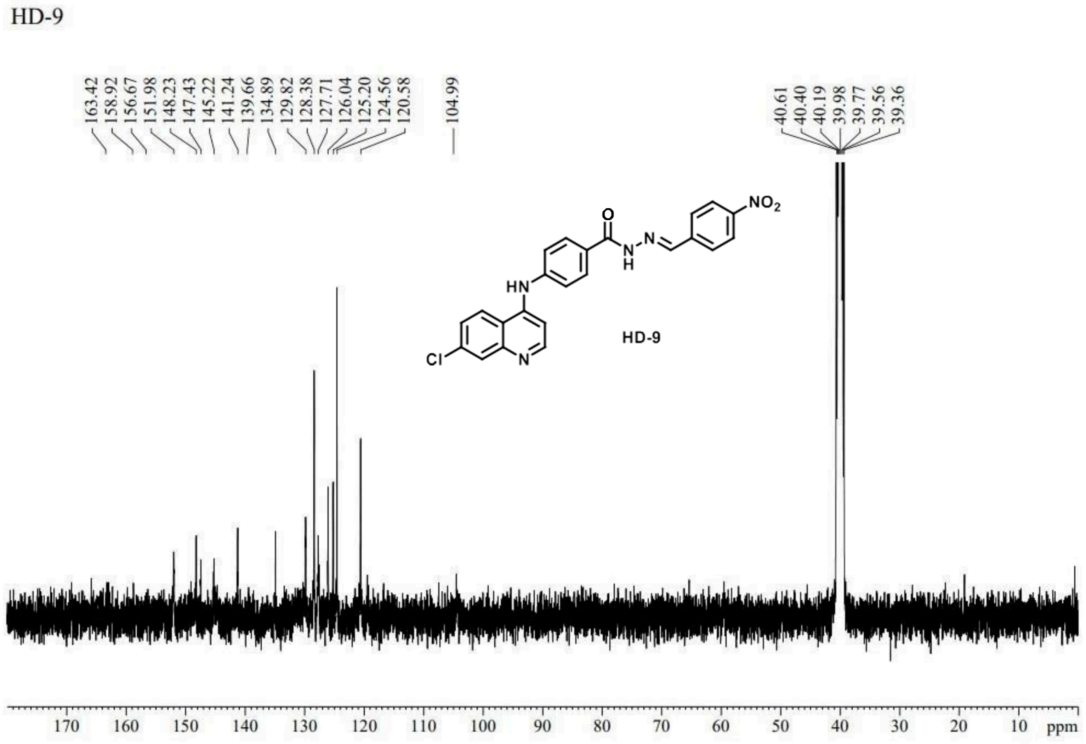

Figure S26: ^{13}C NMR spectrum of **HD9.**

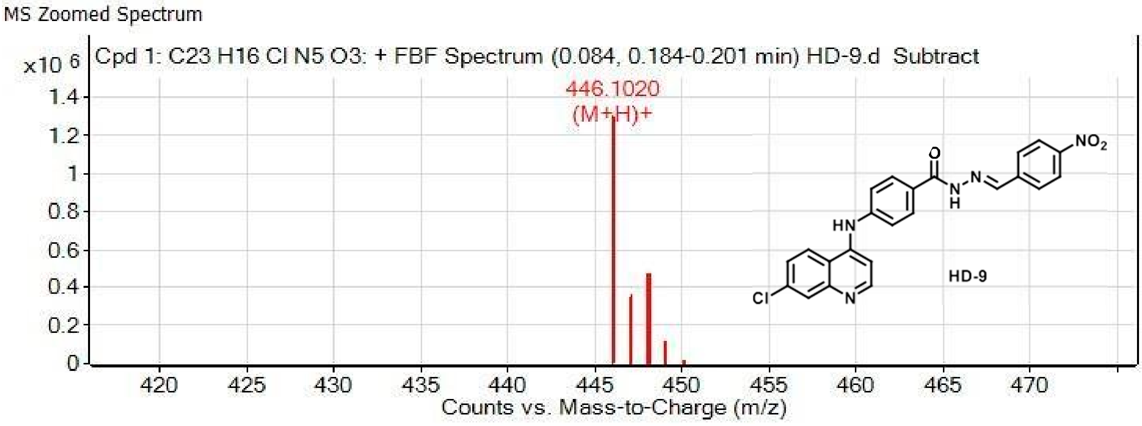

Figure S27: Mass spectrum of **HD**9.

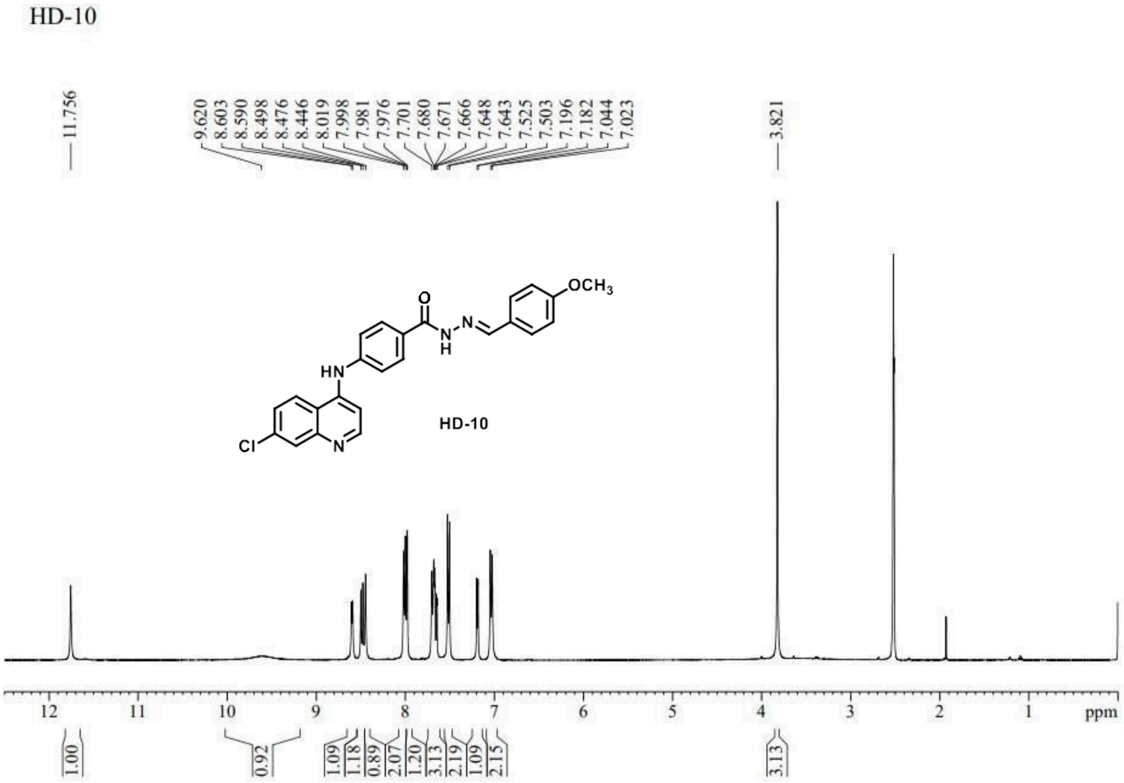

Figure S28: ^{1}H NMR spectrum of **HD10.**

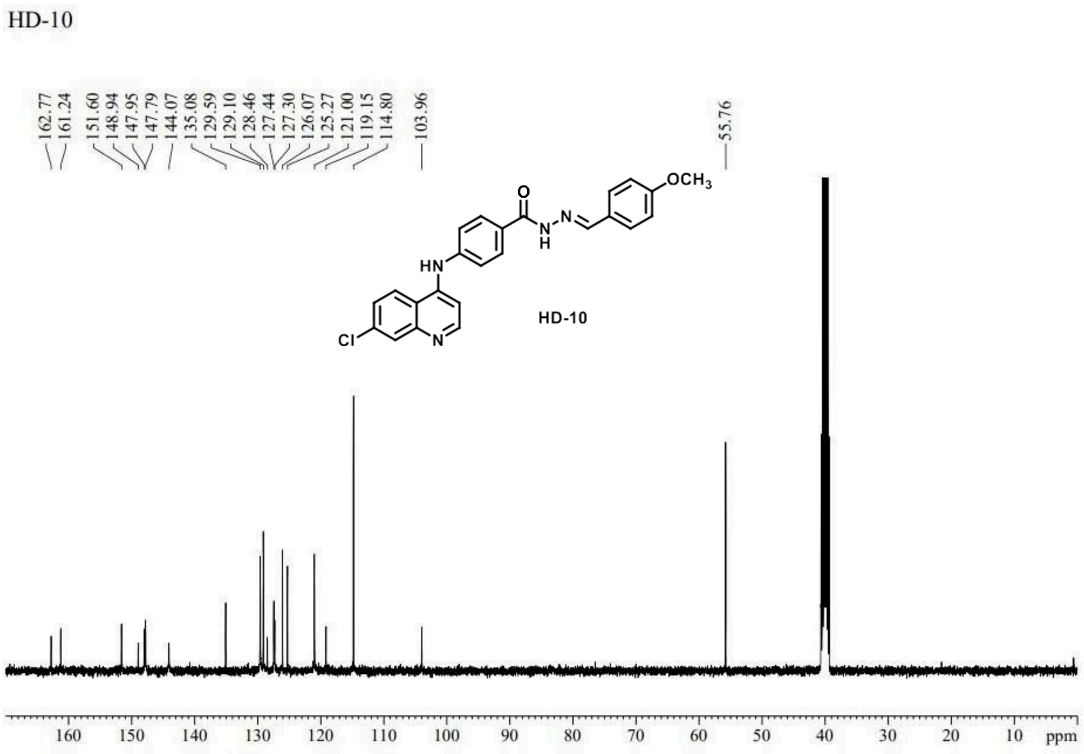

Figure S29: ^{13}C NMR spectrum of **HD10.**

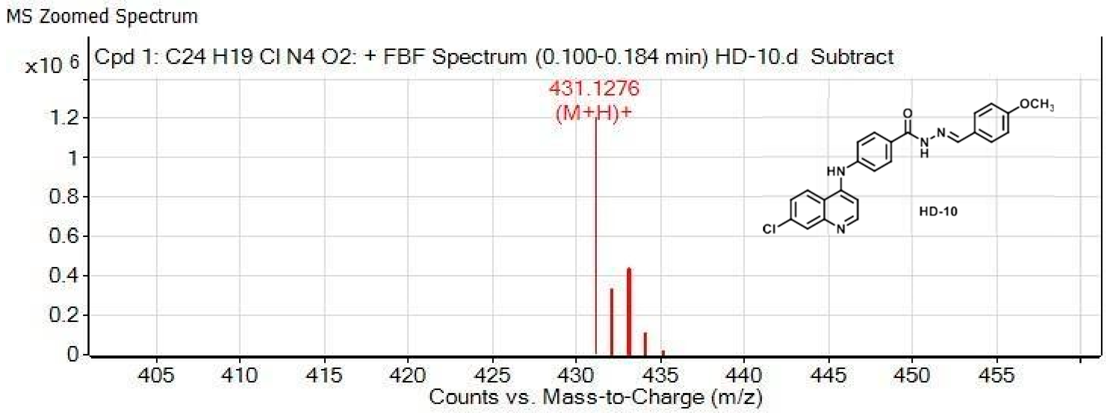

Figure S30: Mass spectrum of **HD10**.

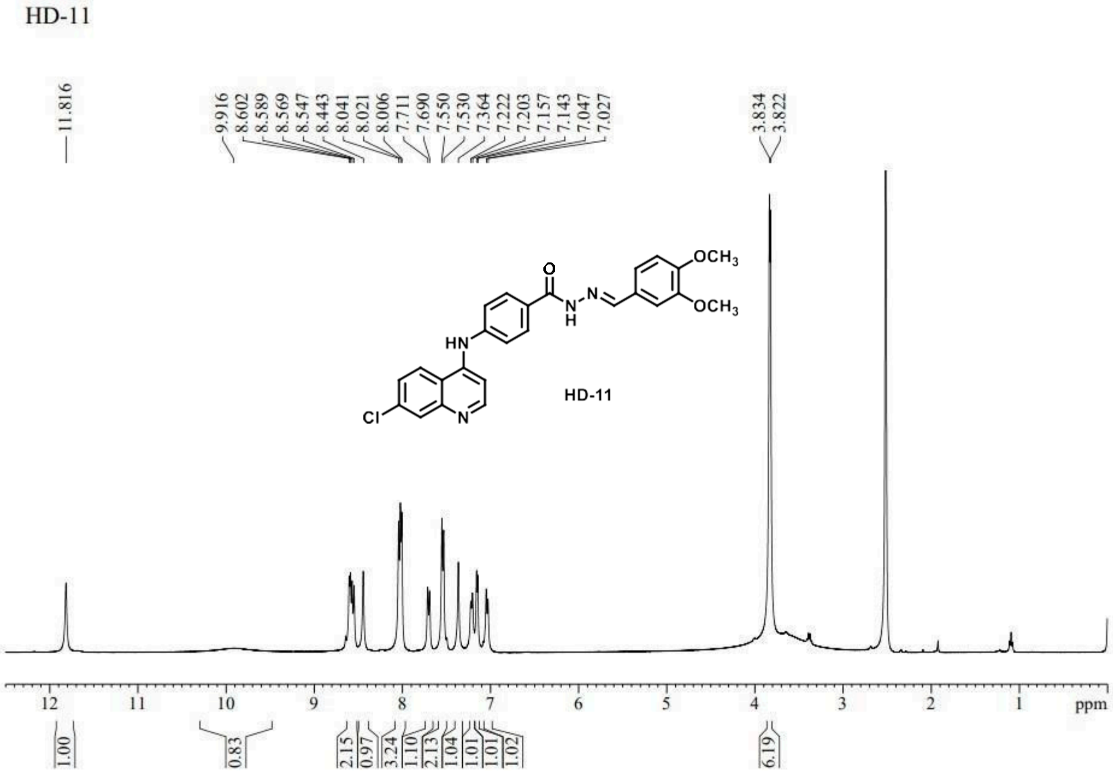

Figure S31: ^{1}H NMR spectrum of **HD11.**

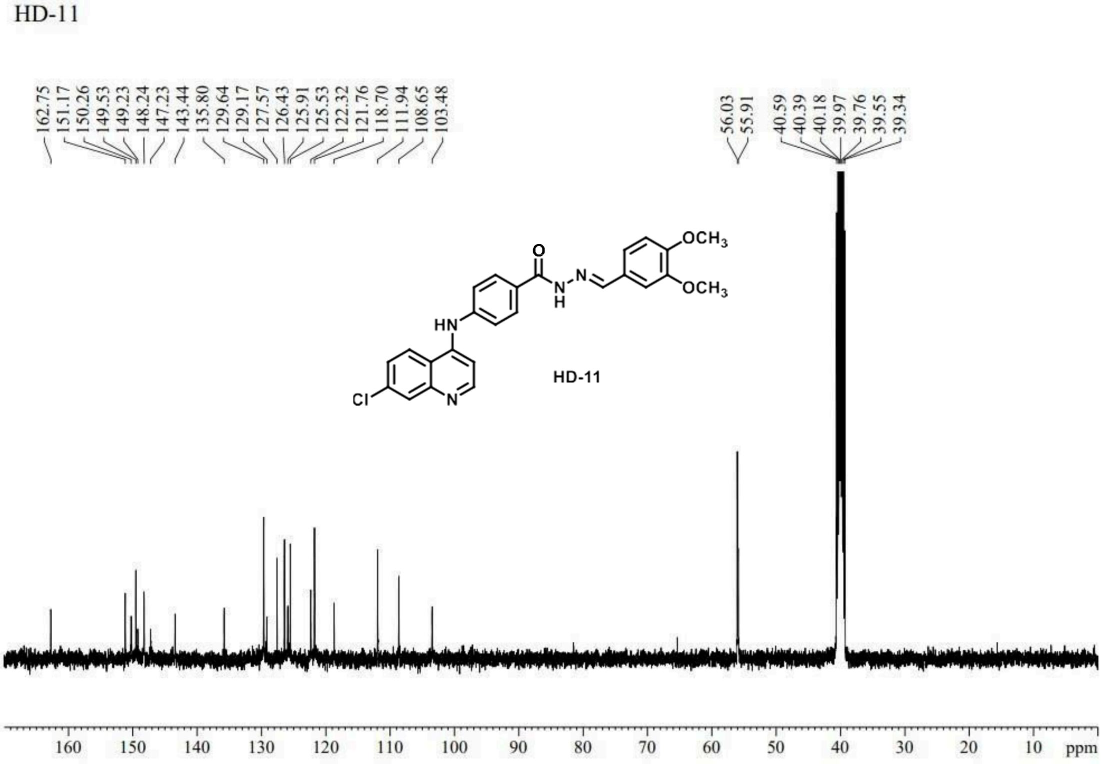

Figure S32: ^{13}C NMR spectrum of **HD11.**

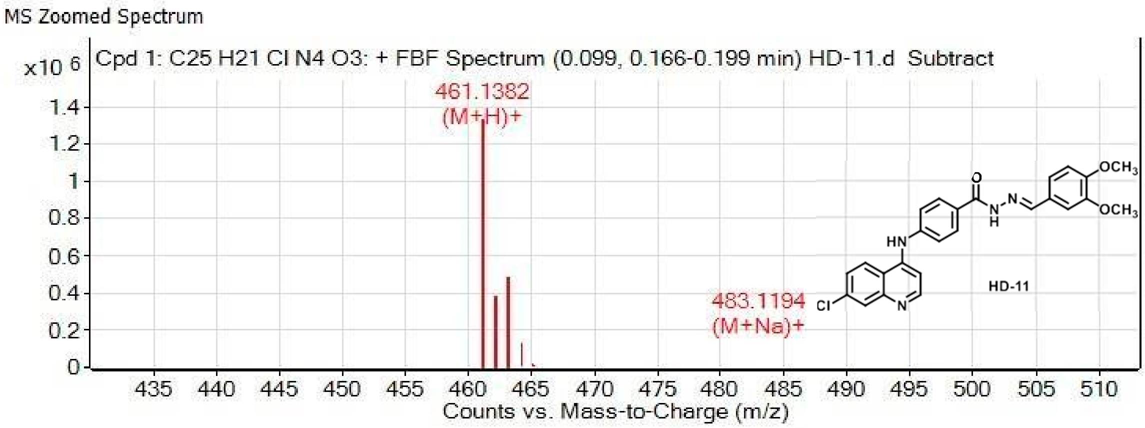

Figure S33: Mass spectrum of **HD11**.

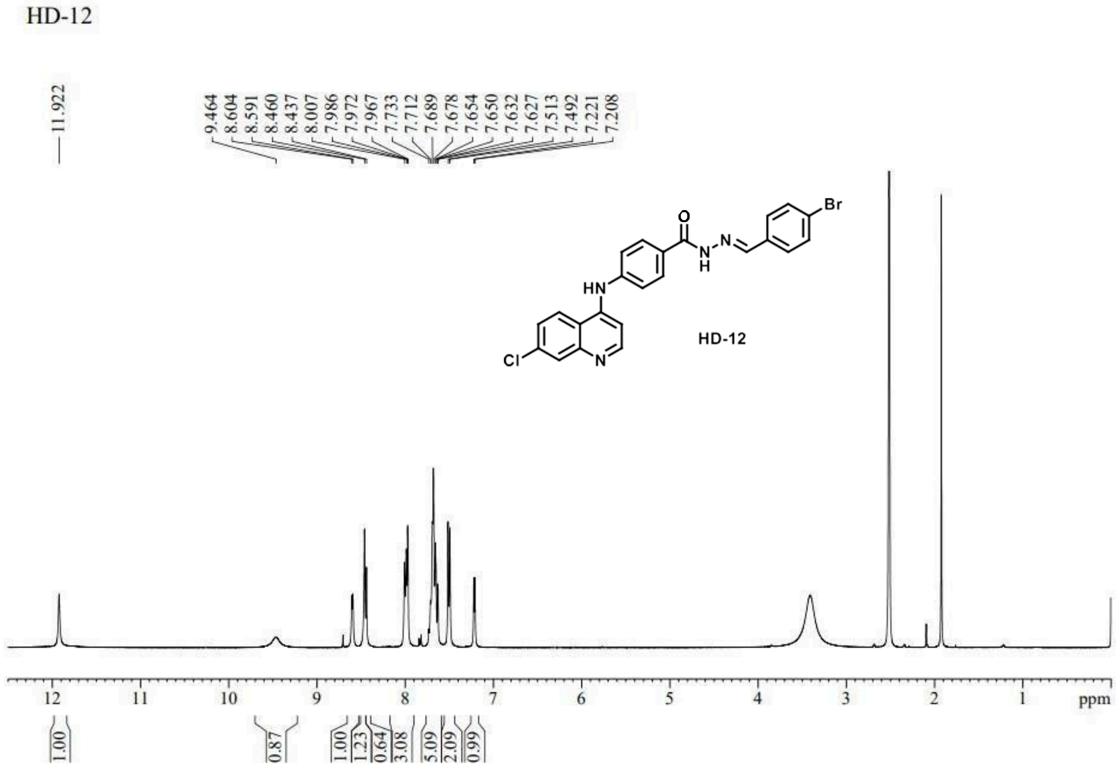

Figure S34: ^{1}H NMR spectrum of **HD12.**

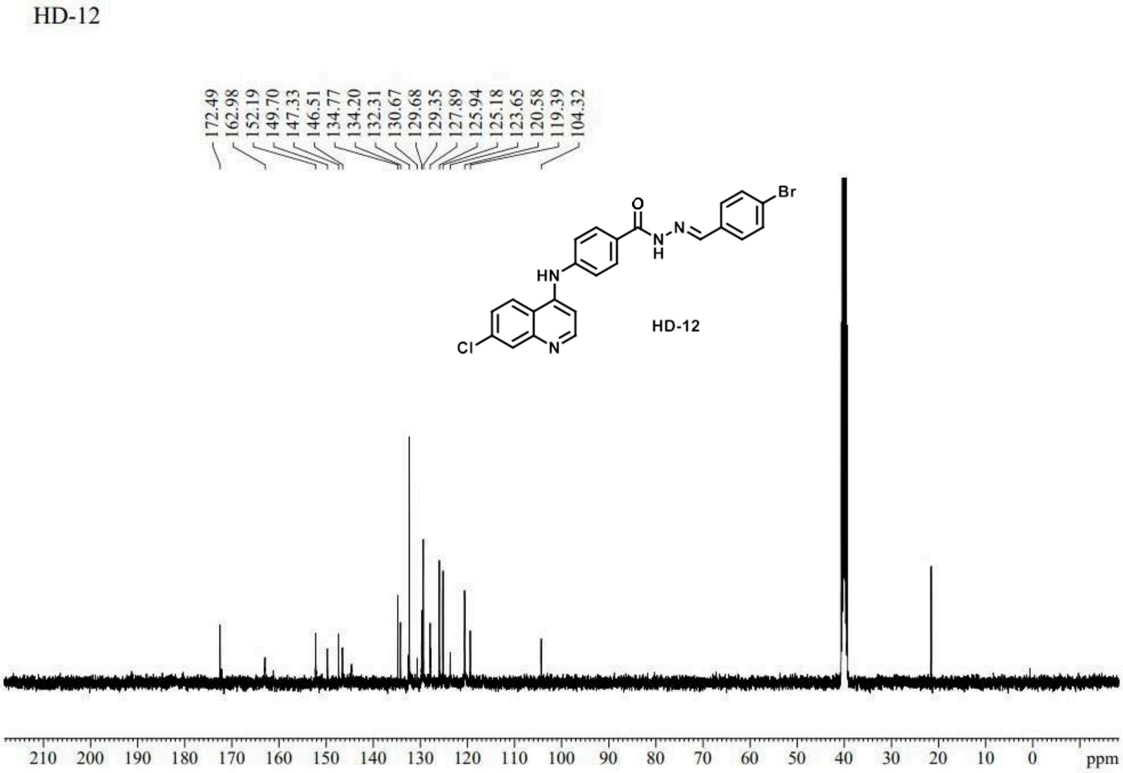

Figure S35: ^{13}C NMR spectrum of **HD12.**

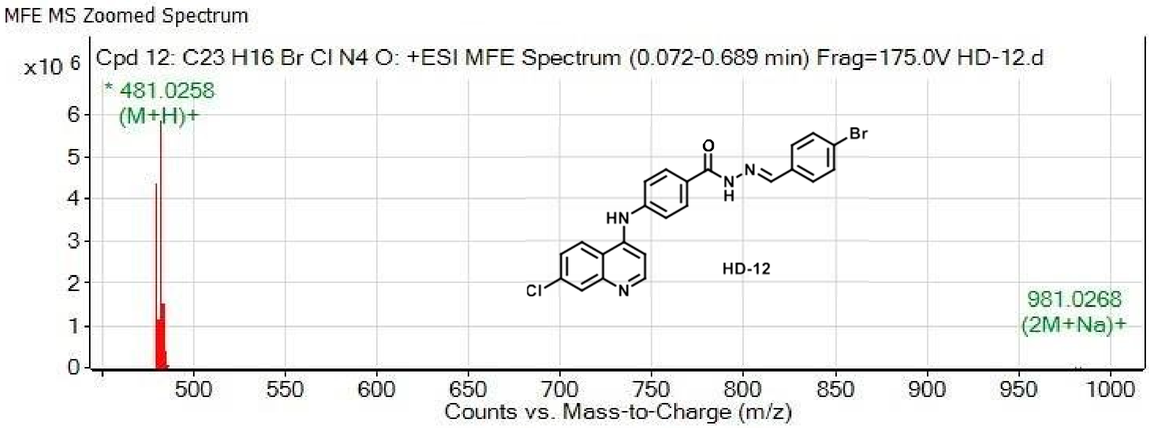

Figure S36: Mass spectrum of **HD12.**

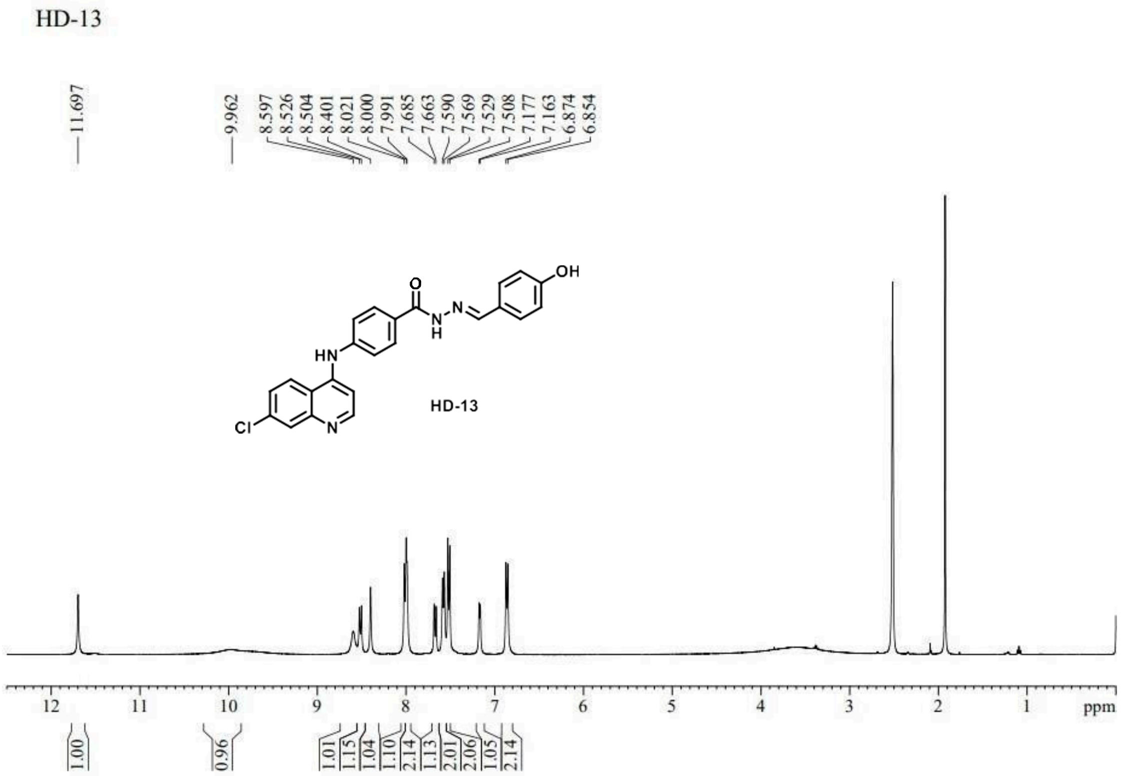

Figure S37: ^{1}H NMR spectrum of **HD13.**

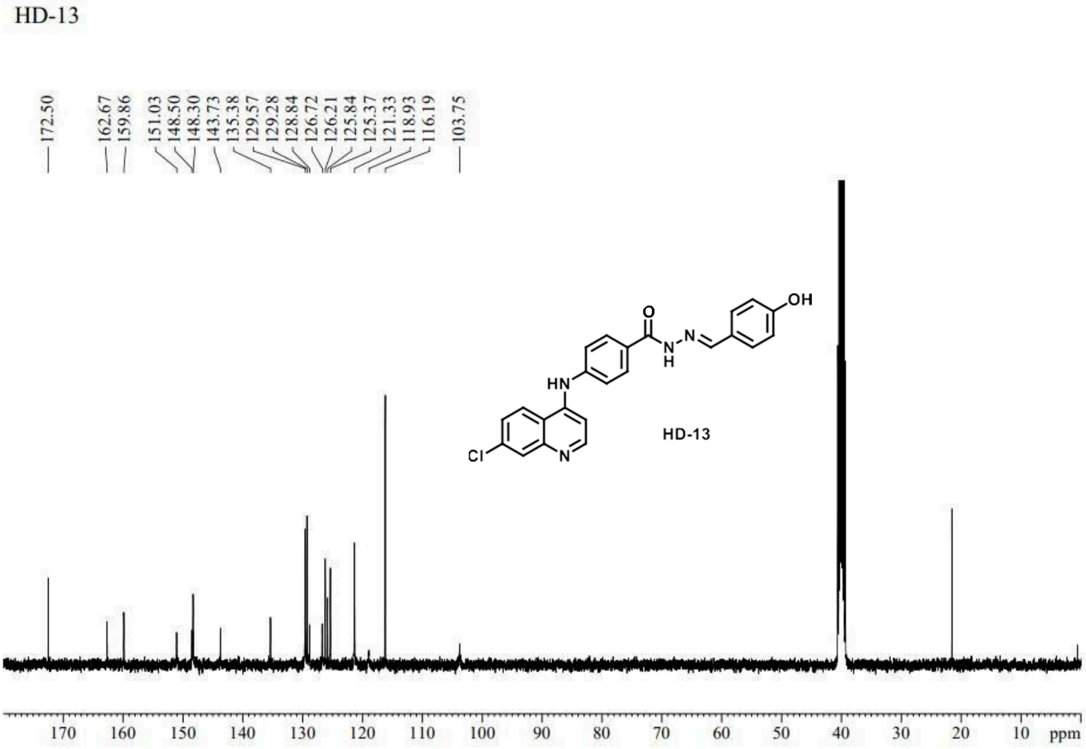

Figure S38: ^{13}C NMR spectrum of **HD13.**

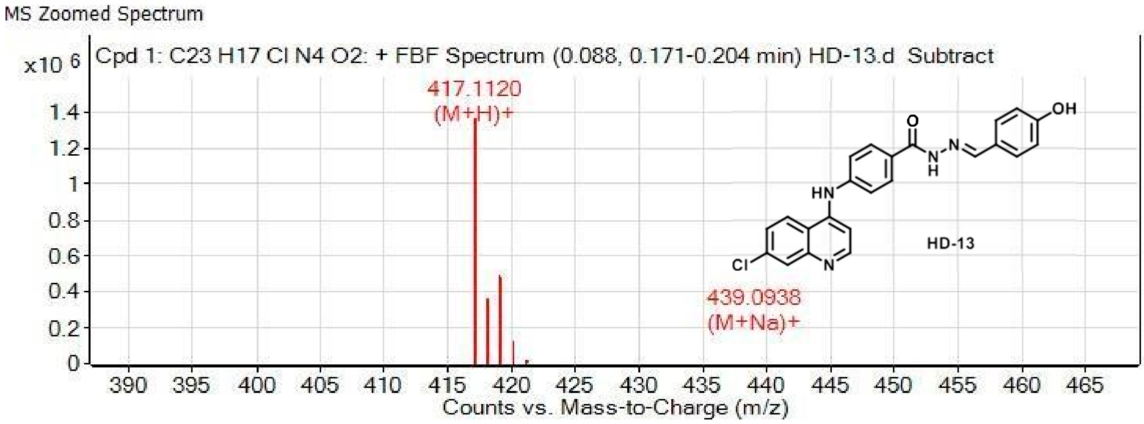

Figure S39: Mass spectrum of **HD13.**

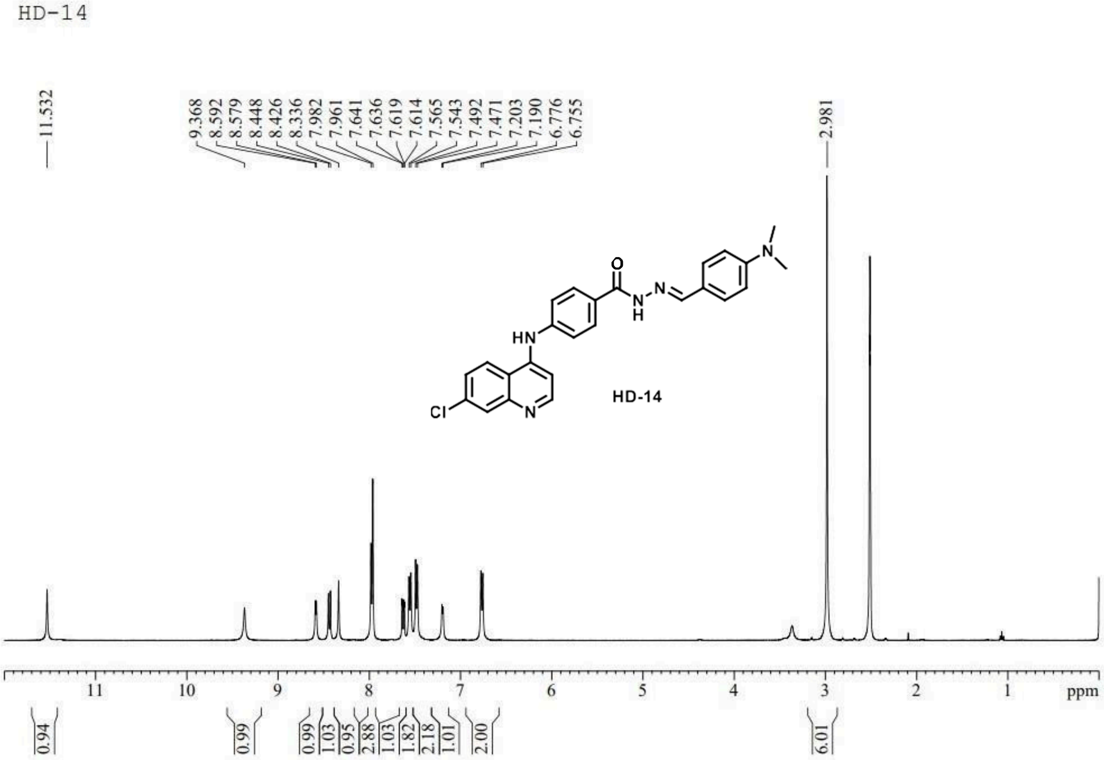

Figure S40: ^{1}H NMR spectrum of **HD14.**

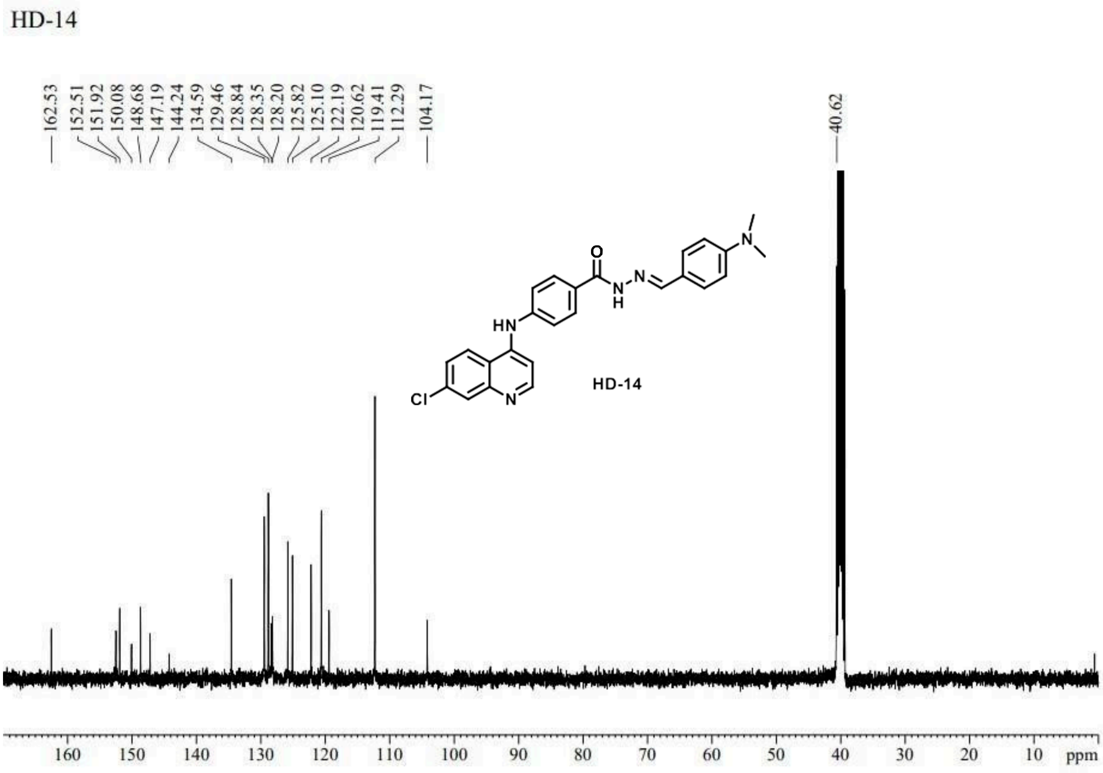

Figure S41: ^{13}C NMR spectrum of **HD14.**

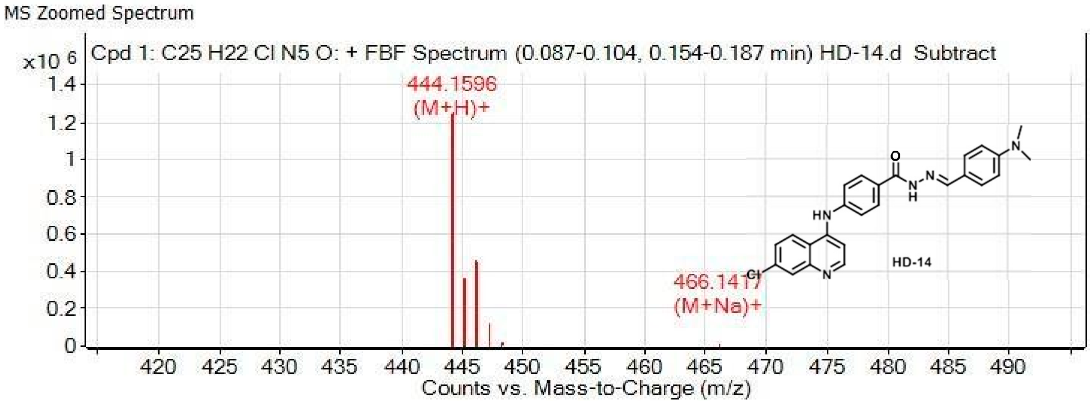

Figure S42: Mass spectrum of **HD14.**

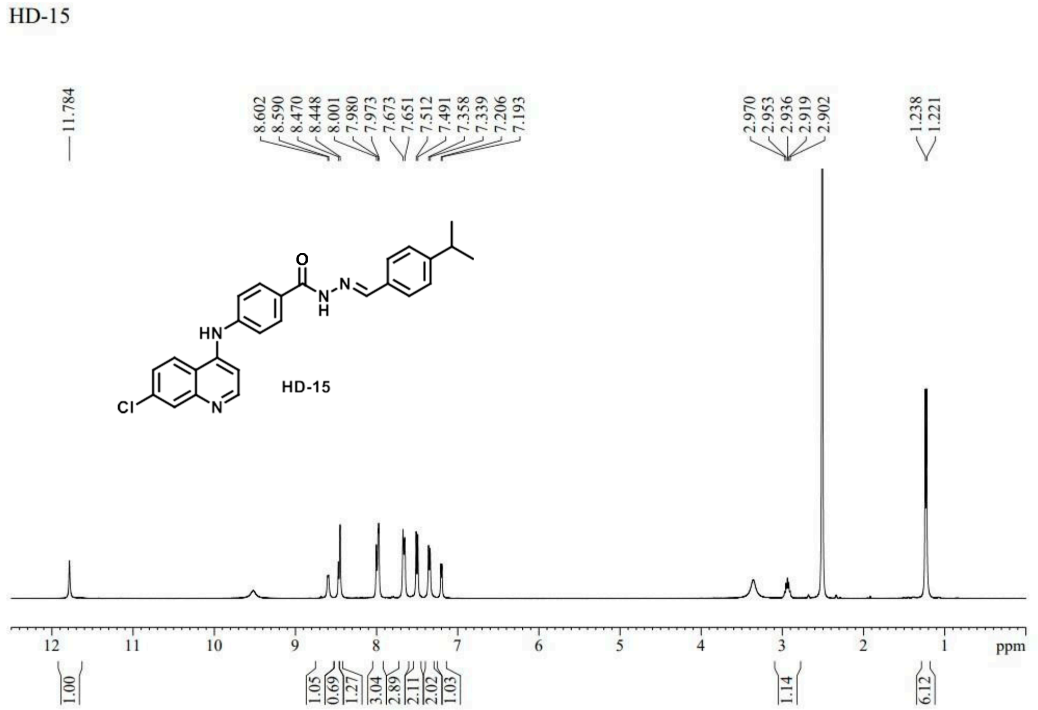

Figure S43: ^{1}H NMR spectrum of **HD15.**

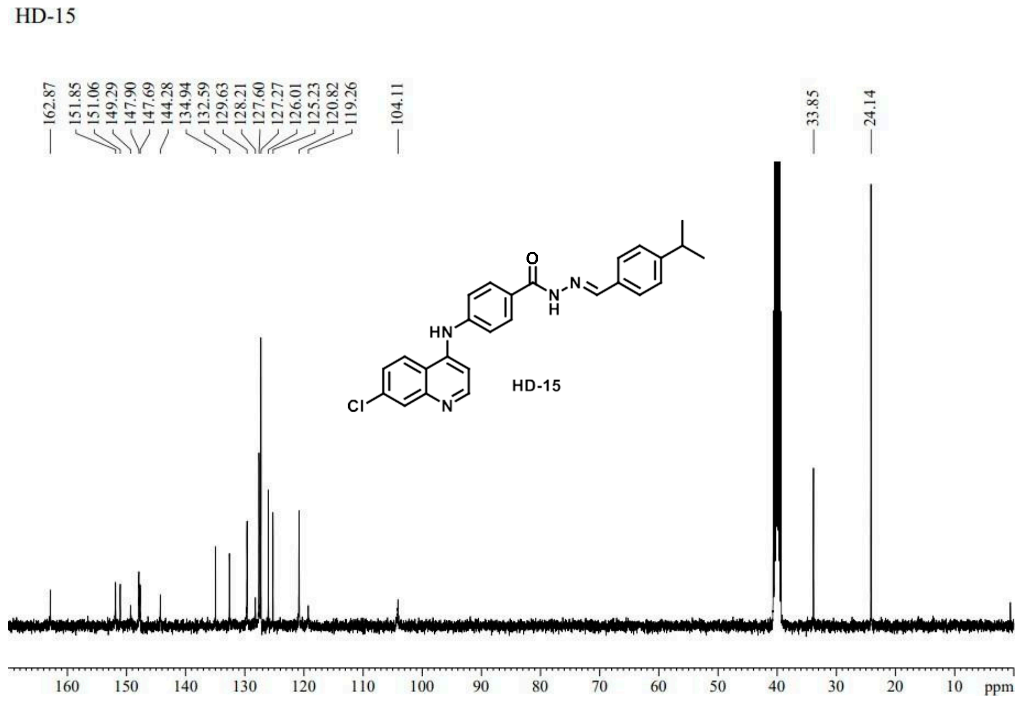

Figure S44: ^{13}C NMR spectrum of **HD15.**

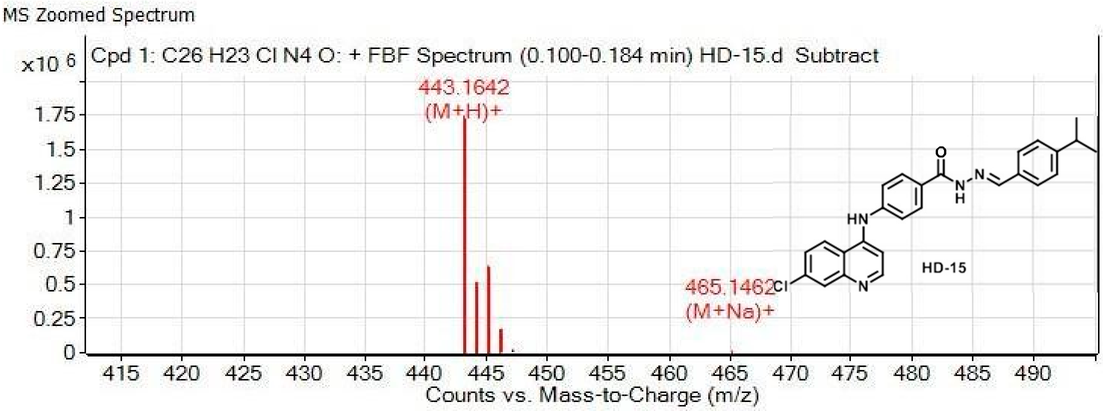

Figure S45: Mass spectrum of **HD15.**

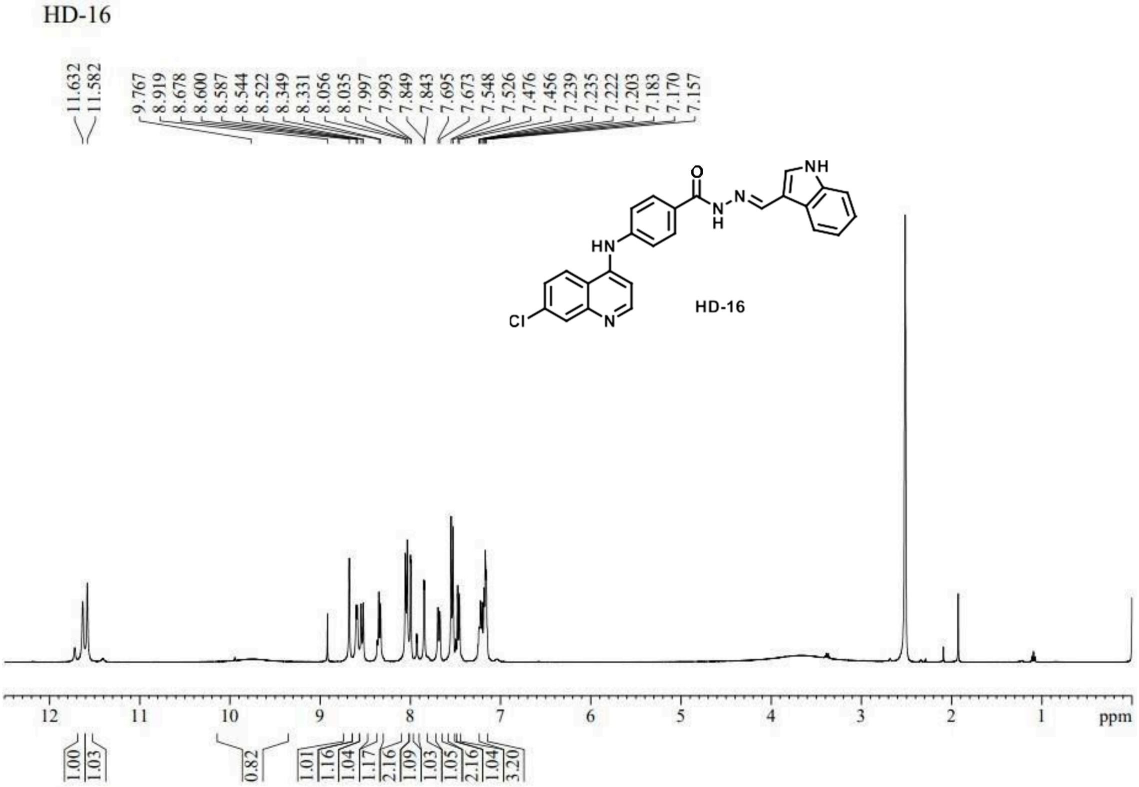

Figure S46: ^{1}H NMR spectrum of **HD16.**

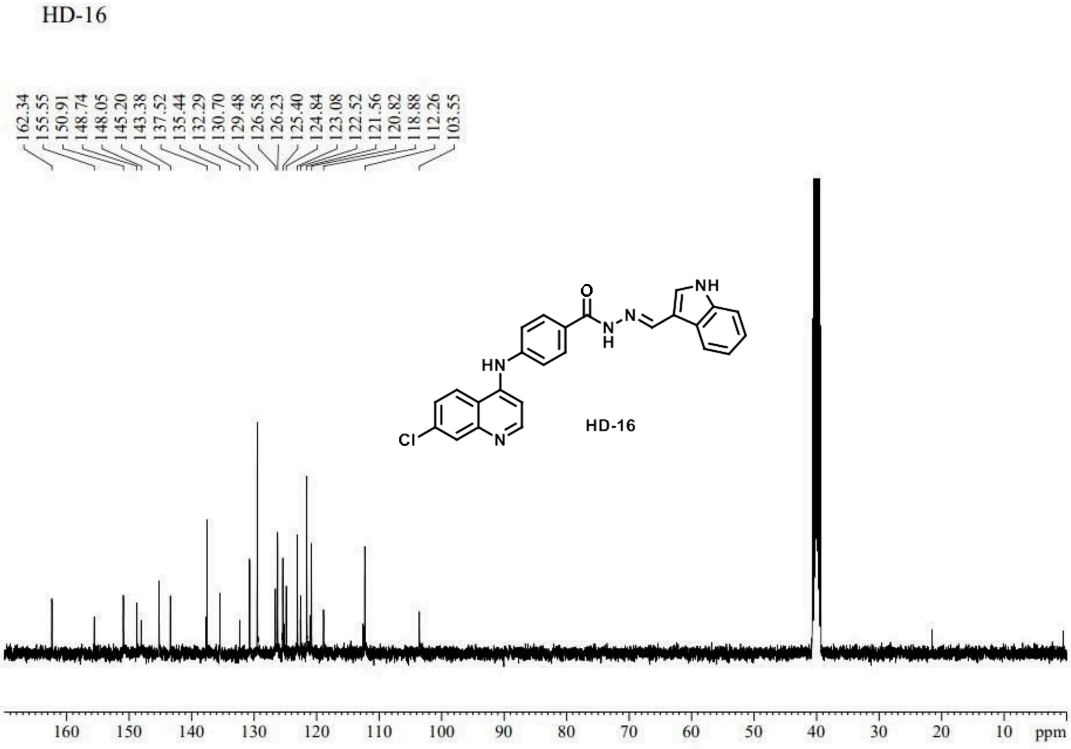

Figure S47: ^{13}C NMR spectrum of **HD16.**

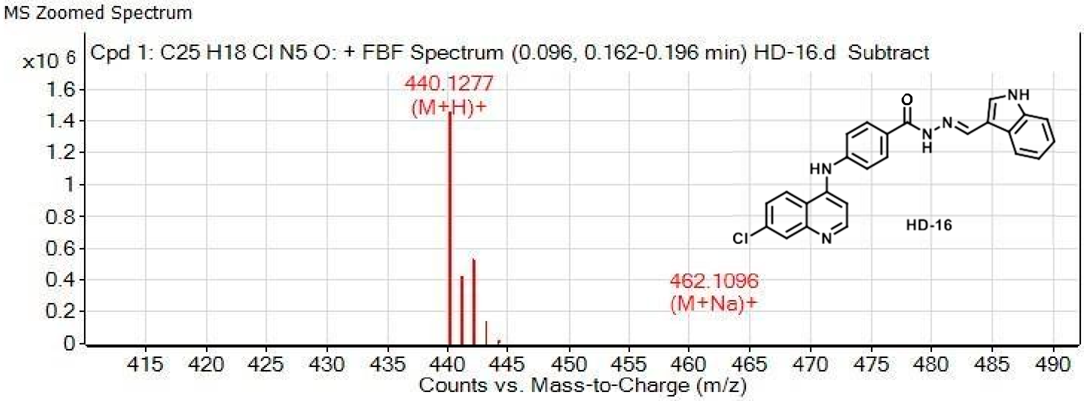

Figure S48: Mass spectrum of **HD16.**

HD-17

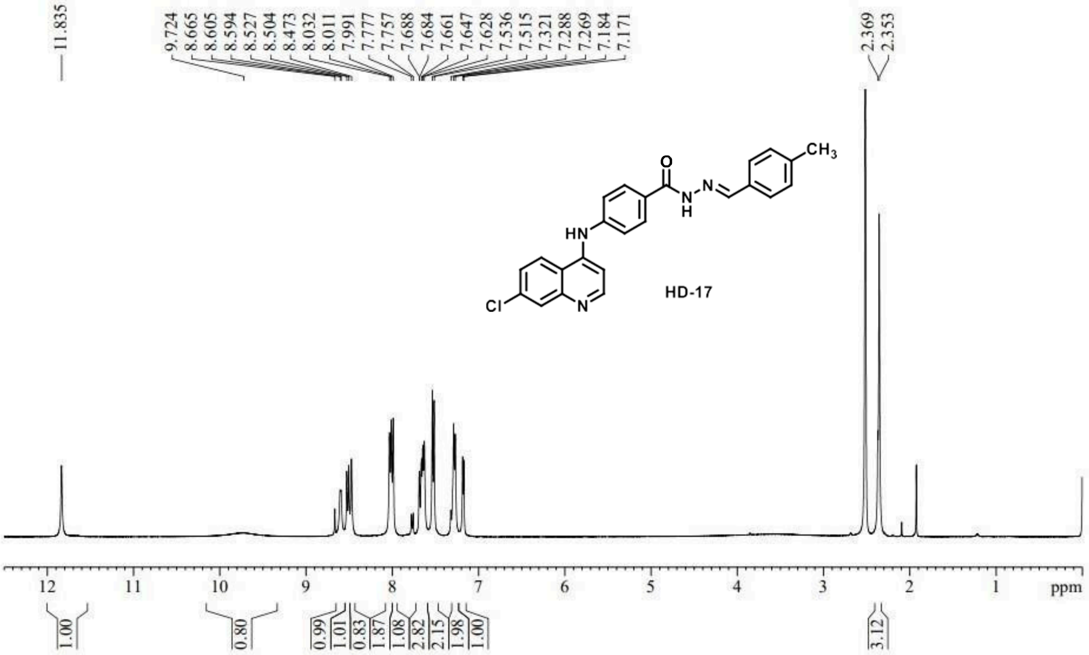

Figure S49: ^{1}H NMR spectrum of **HD17.**

HD-17

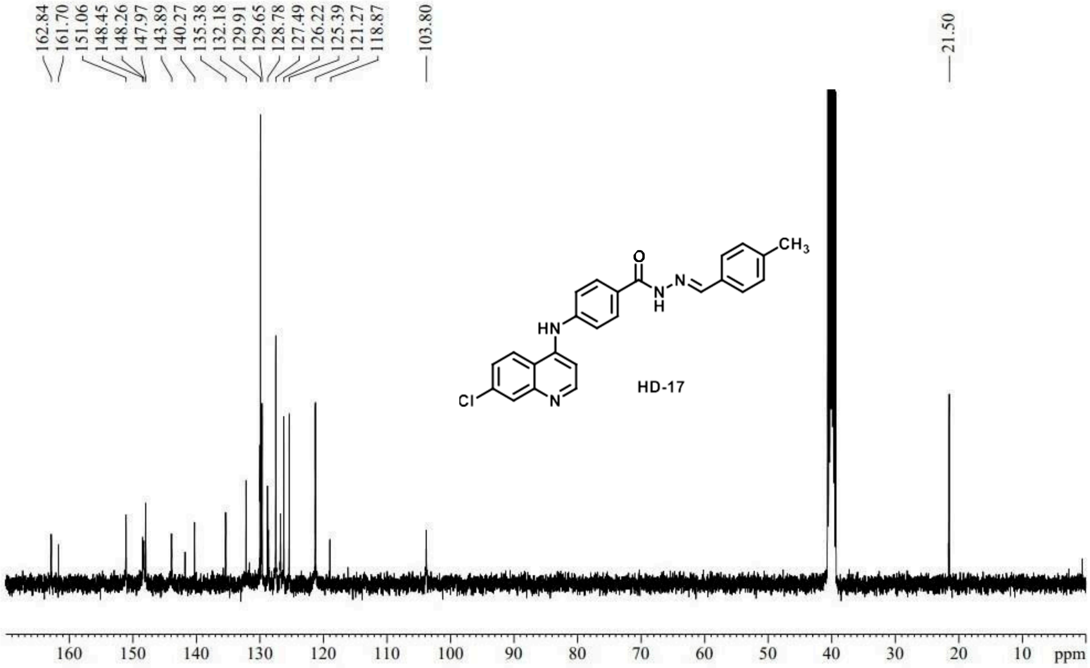

Figure S50: ^{13}C NMR spectrum of **HD17.**

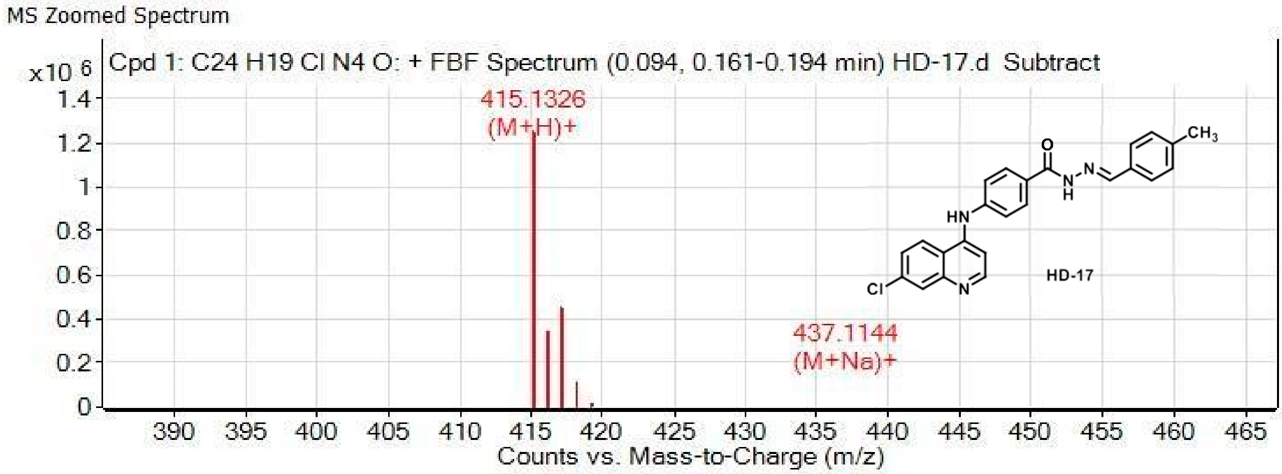

Figure S51: Mass spectrum of **HD17.**

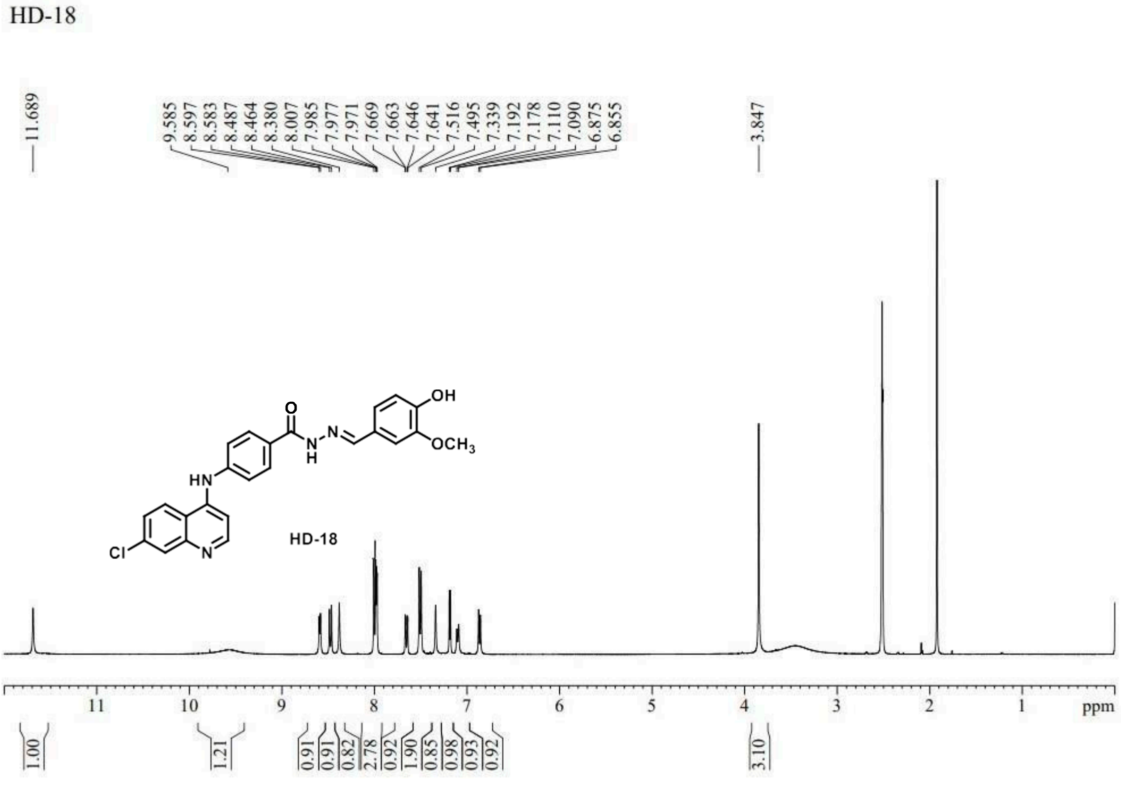

Figure S52: ^{1}H NMR spectrum of **HD18.**

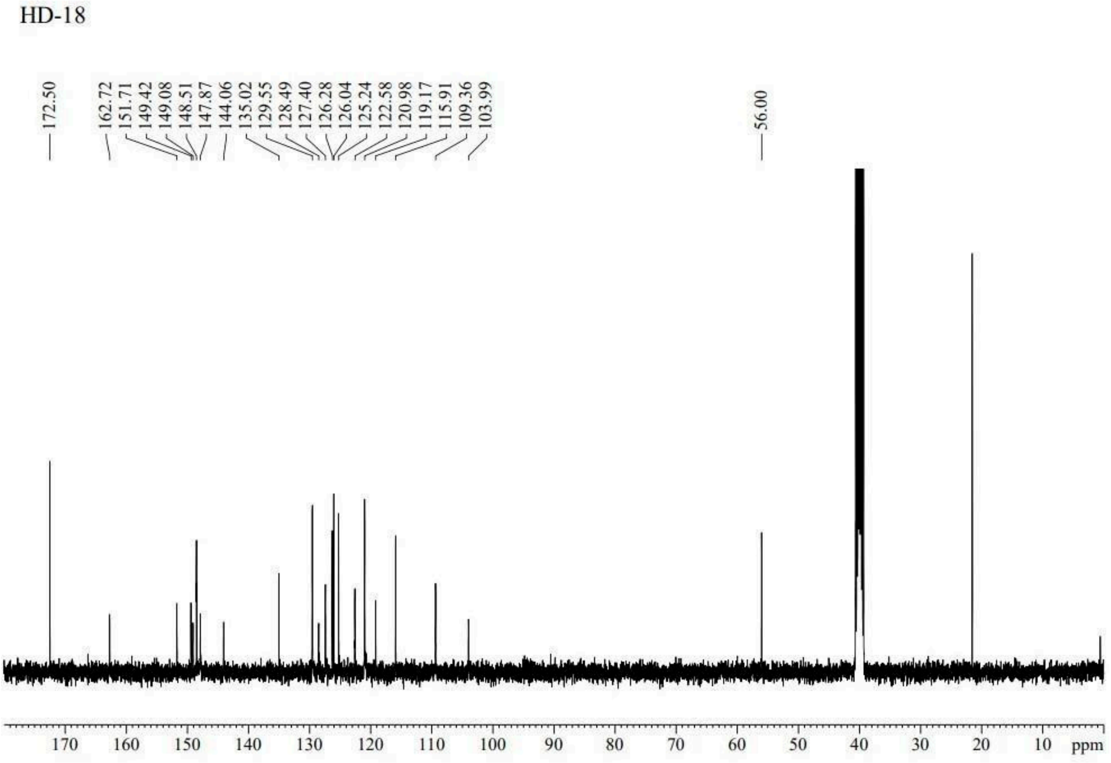

Figure S53: ^{13}C NMR spectrum of **HD18.**

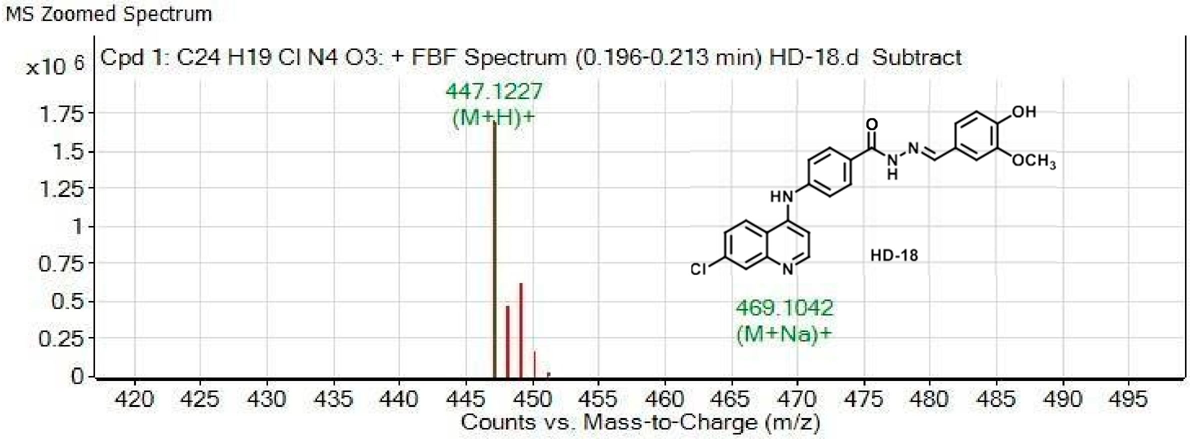

Figure S54: Mass spectrum of **HD18.**

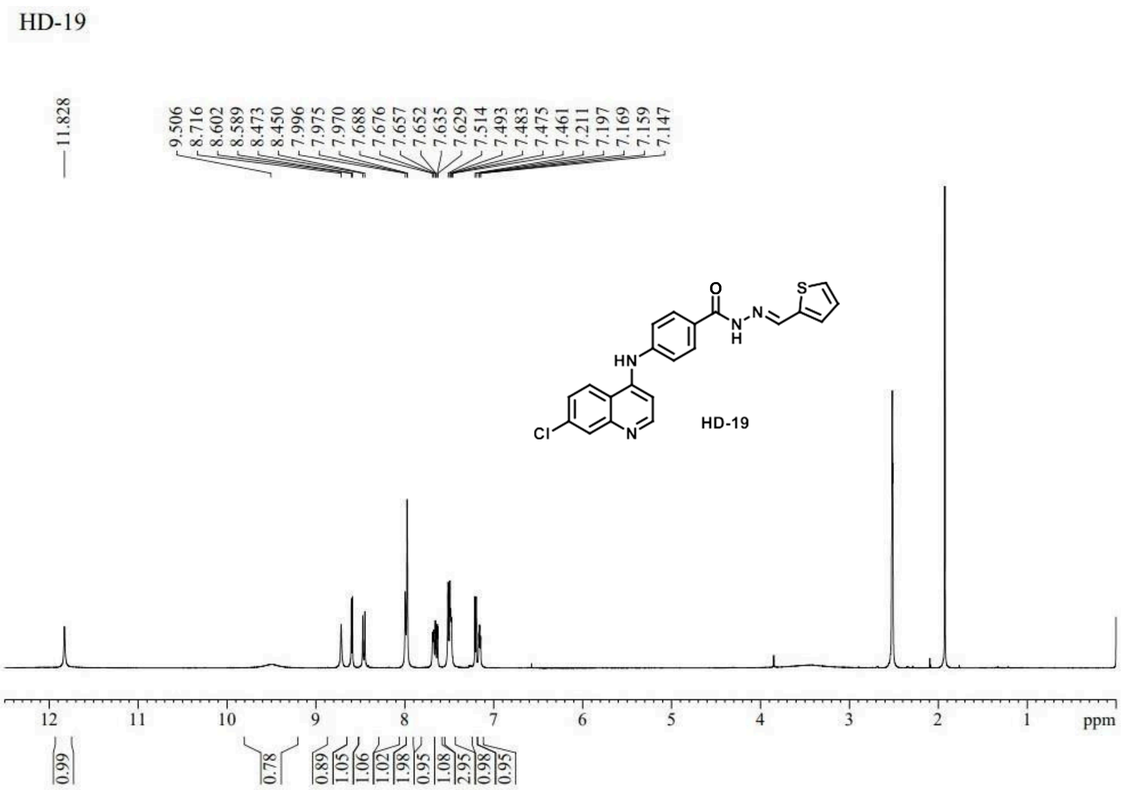

Figure S55: ^{1}H NMR spectrum of **HD19.**

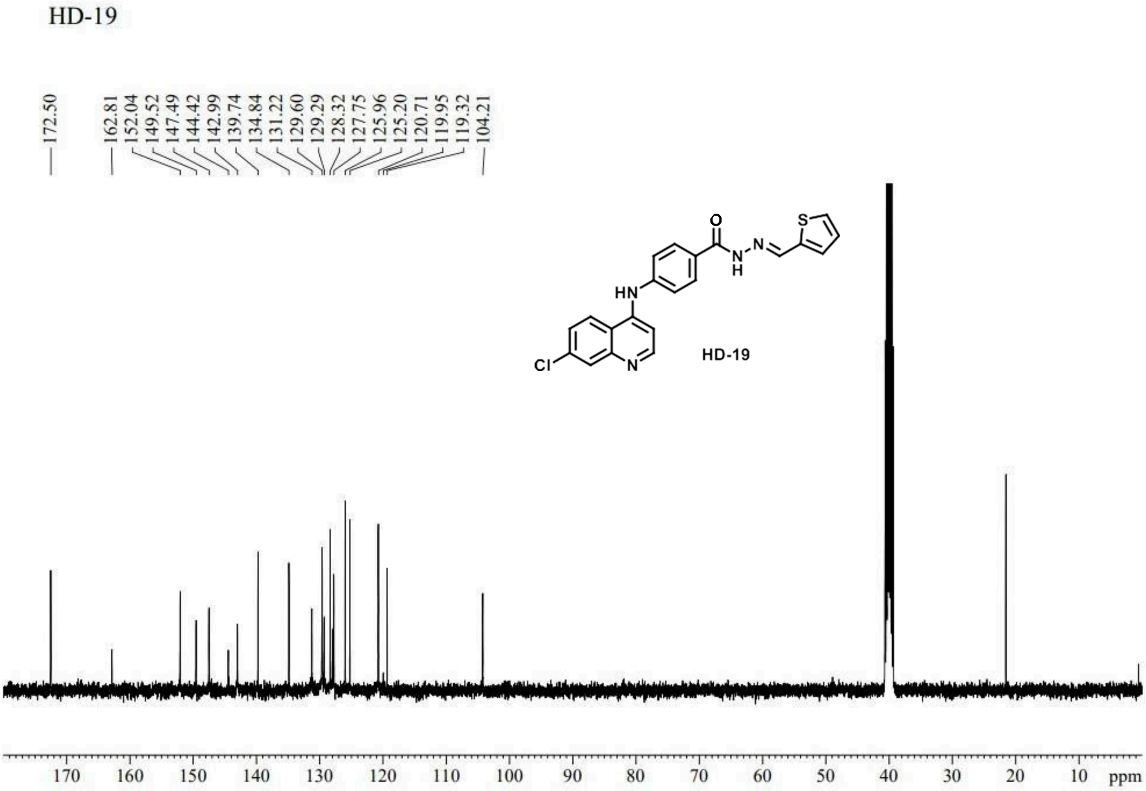

Figure S56: ^{13}C NMR spectrum of **HD19.**

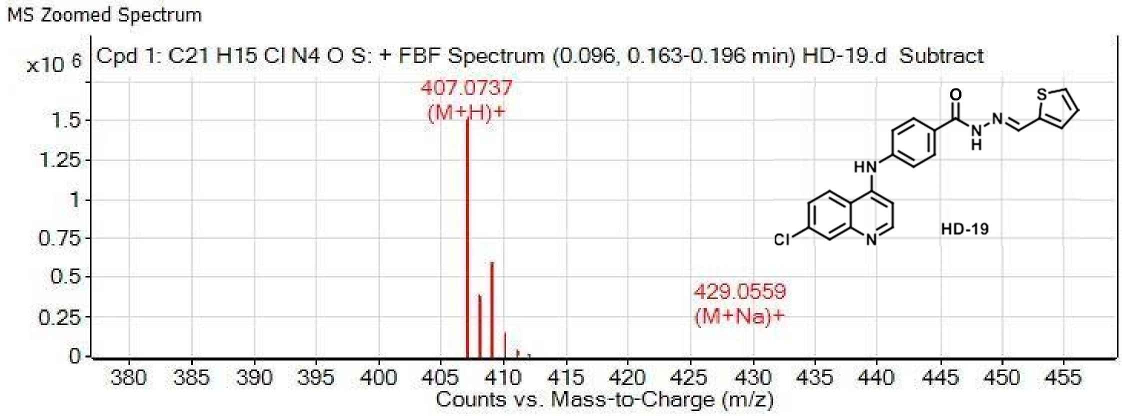

Figure S57: Mass spectrum of **HD19.**

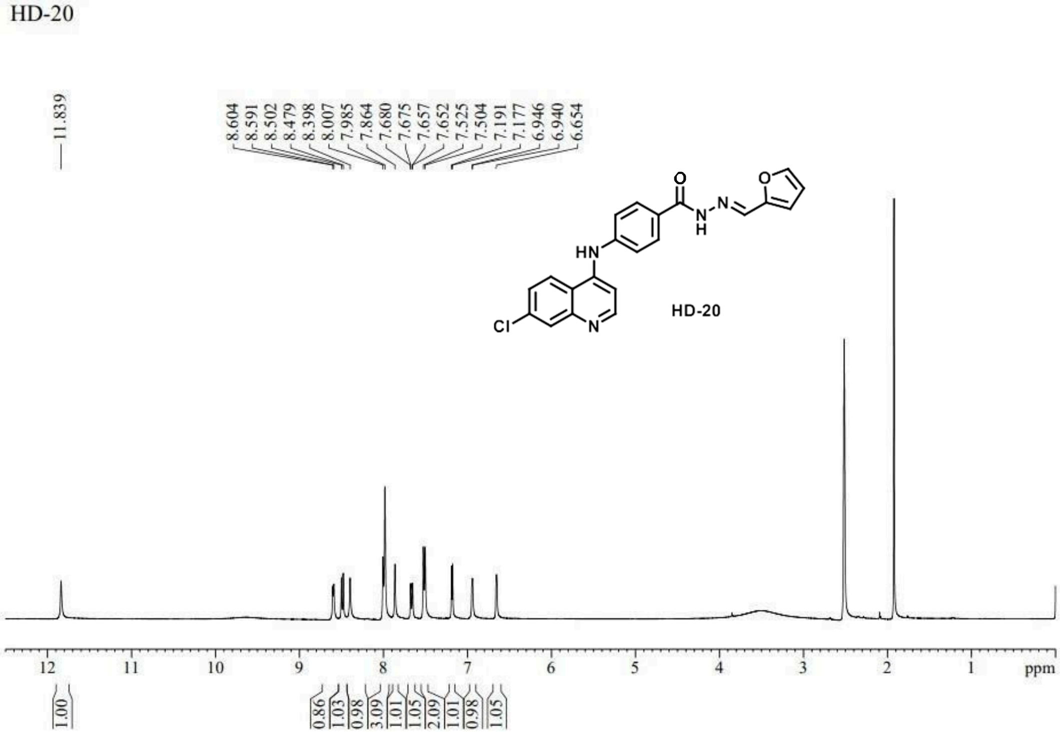

Figure S58: ^{1}H NMR spectrum of **HD20.**

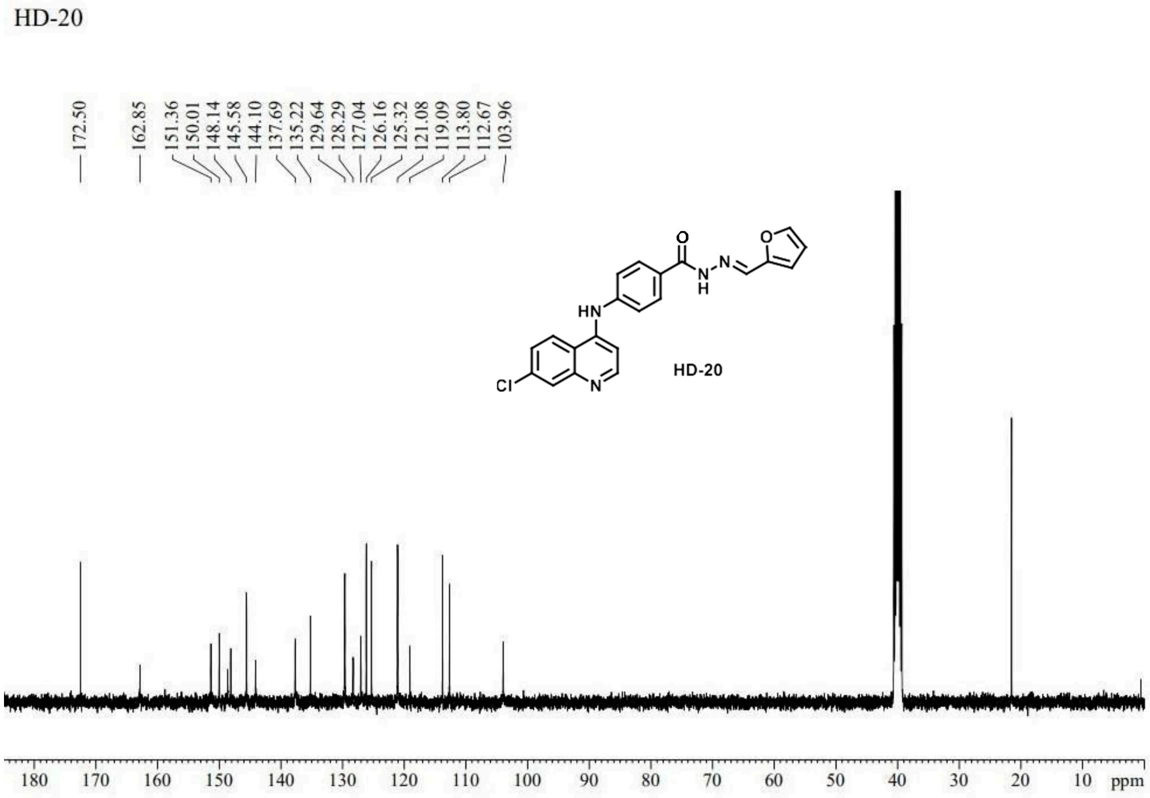

Figure S59: ^{13}C NMR spectrum of **HD20.**

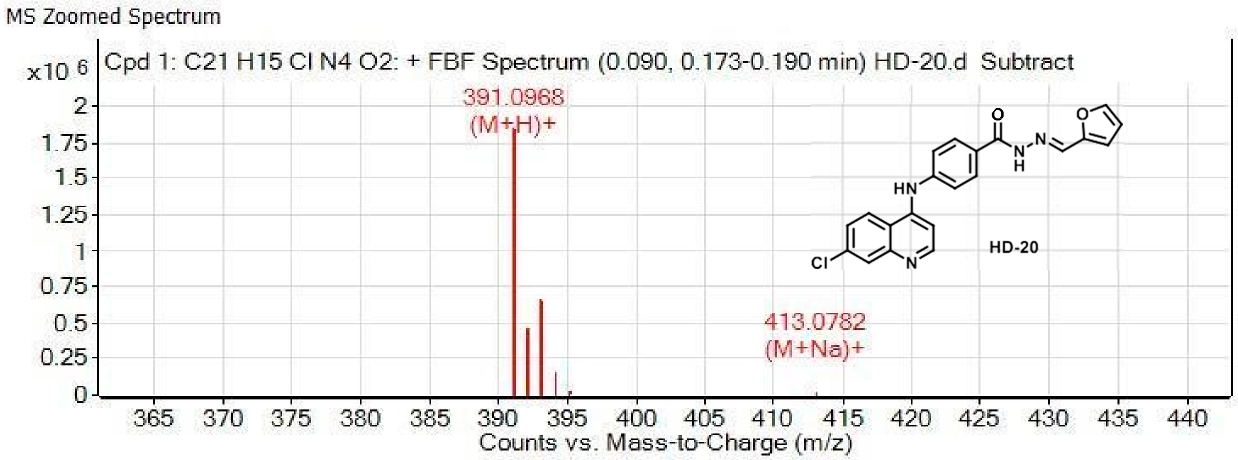

Figure S60: Mass spectrum of **HD20.**

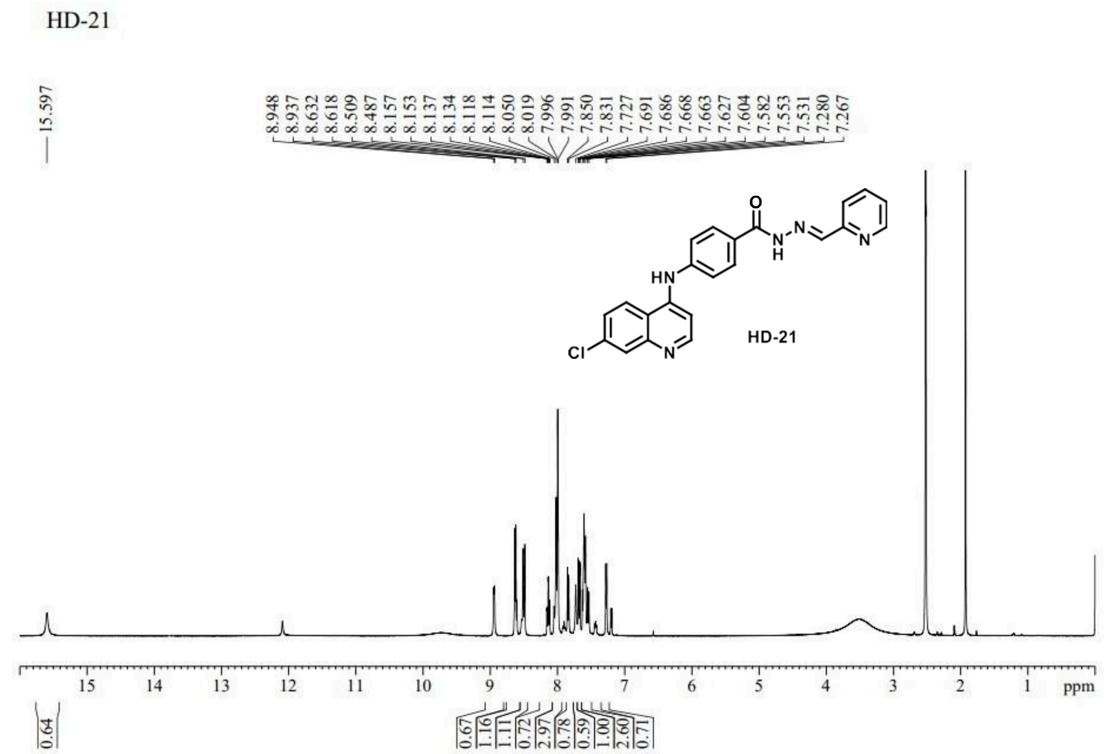

Figure S61: ^{1}H NMR spectrum of **HD21.**

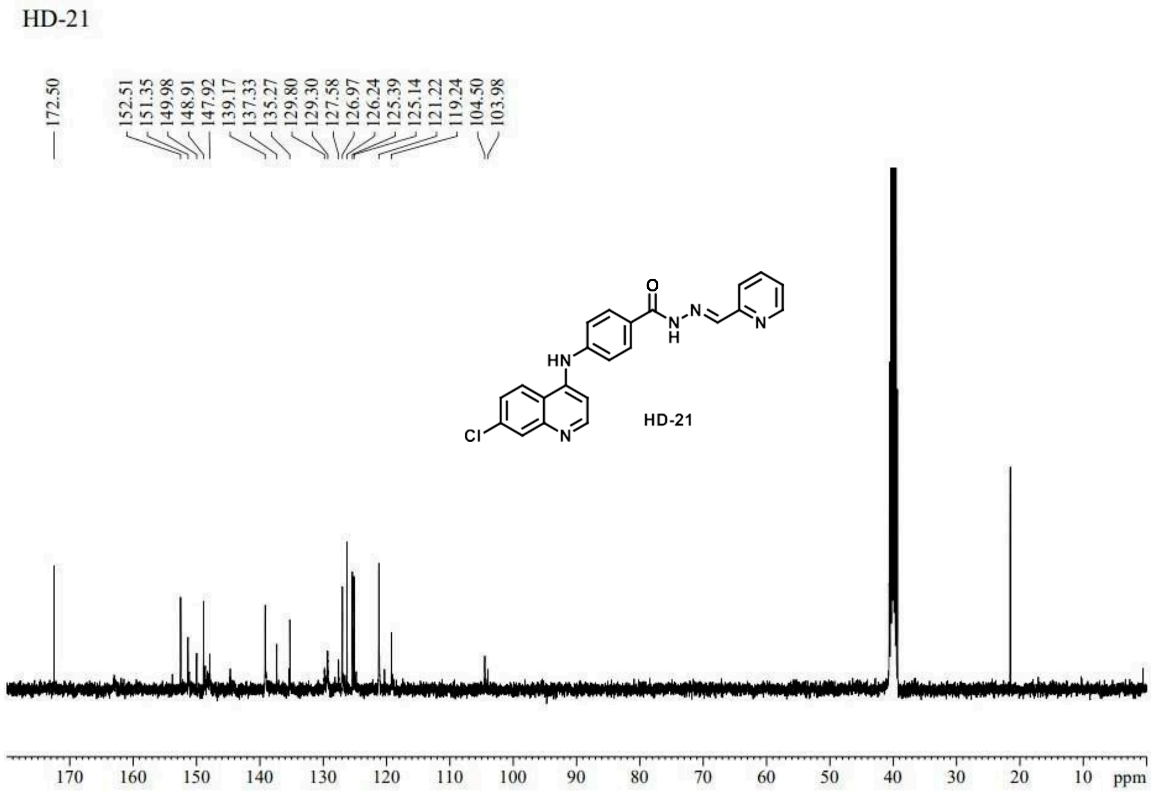

Figure S62: ^{13}C NMR spectrum of **HD21.**

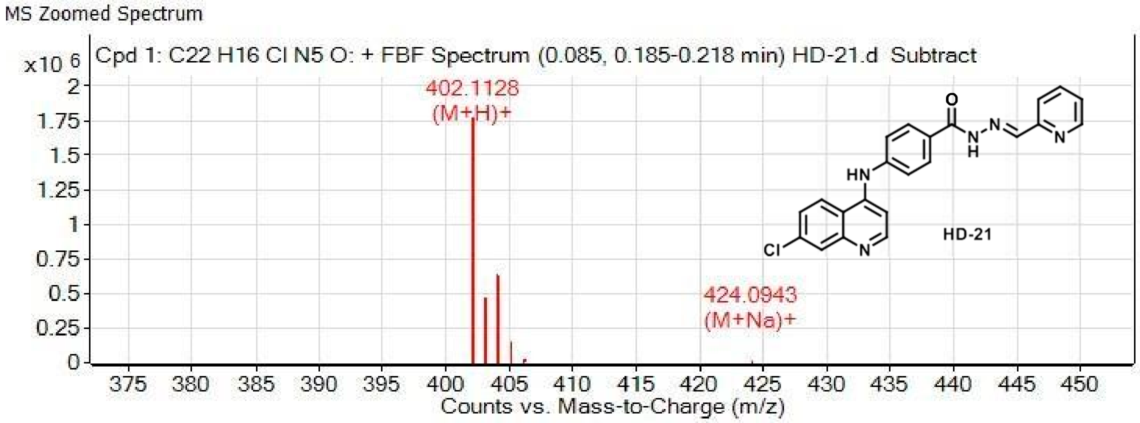

Figure S63: Mass spectrum of **HD21.**

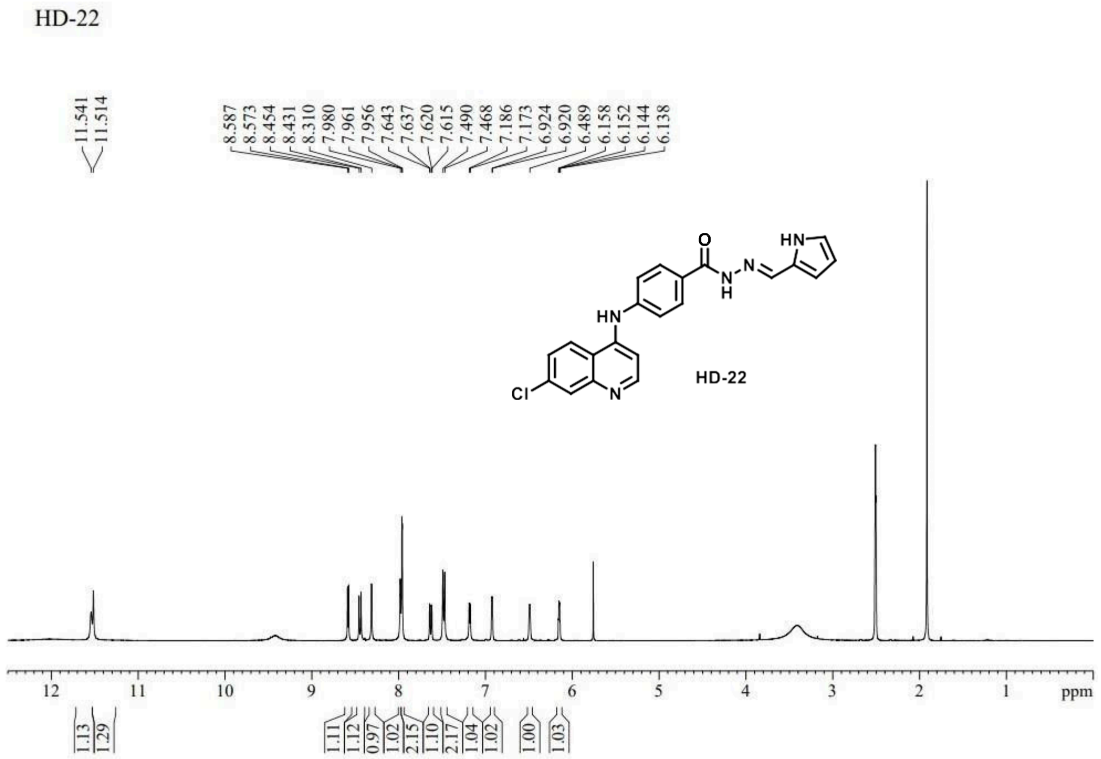

Figure S64: ^{1}H NMR spectrum of **HD22.**

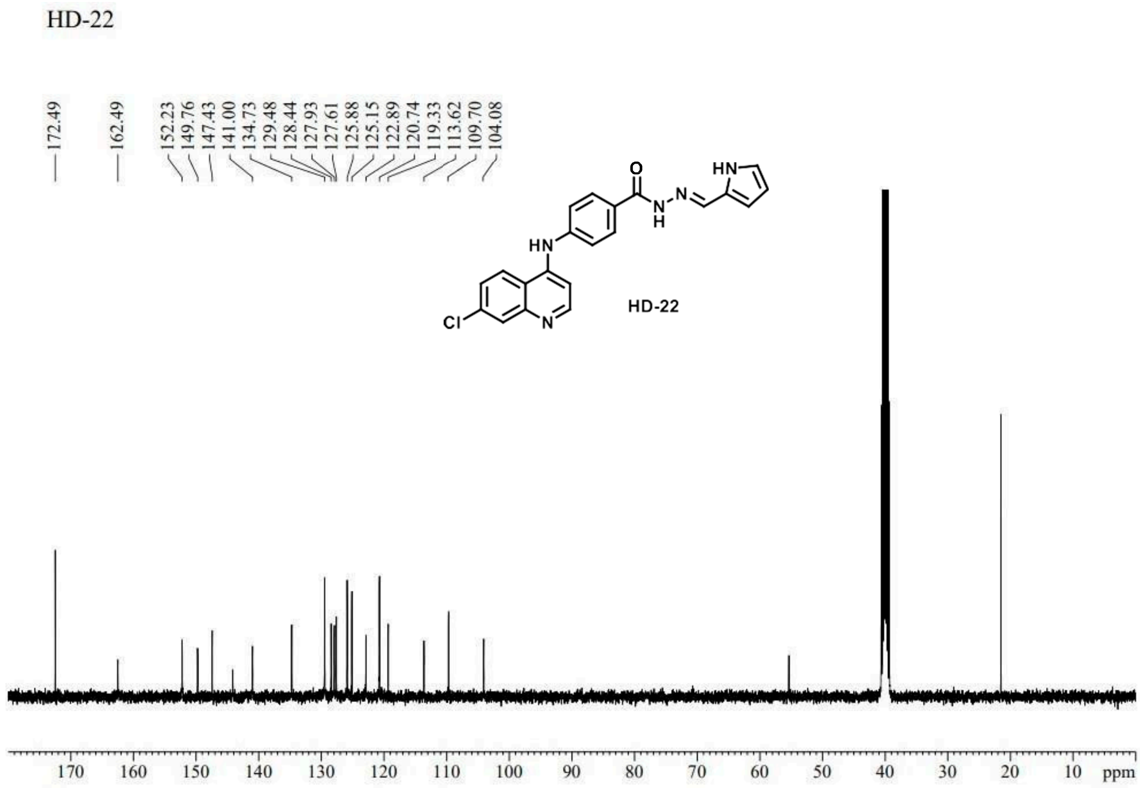

Figure S65: ^{13}C NMR spectrum of **HD22.**

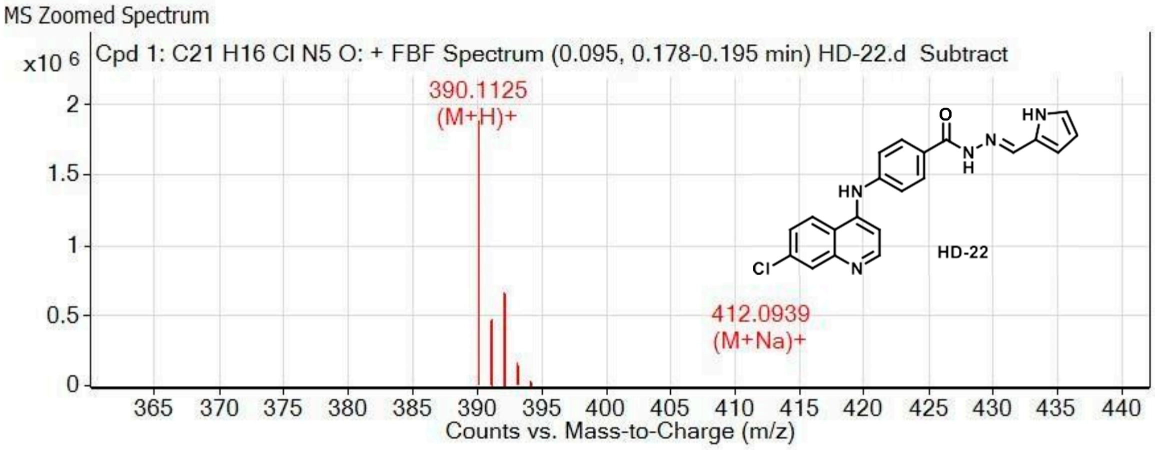

Figure S66: Mass spectrum of **HD22.**

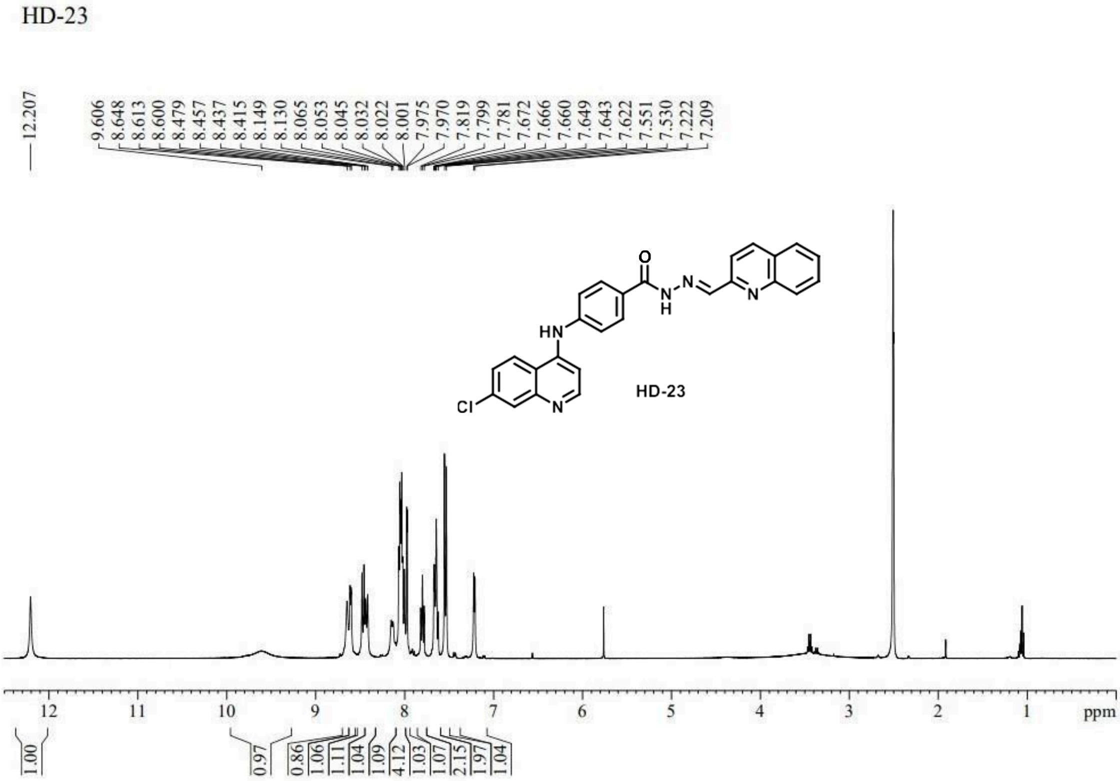

Figure S67: ^{1}H NMR spectrum of **HD23.**

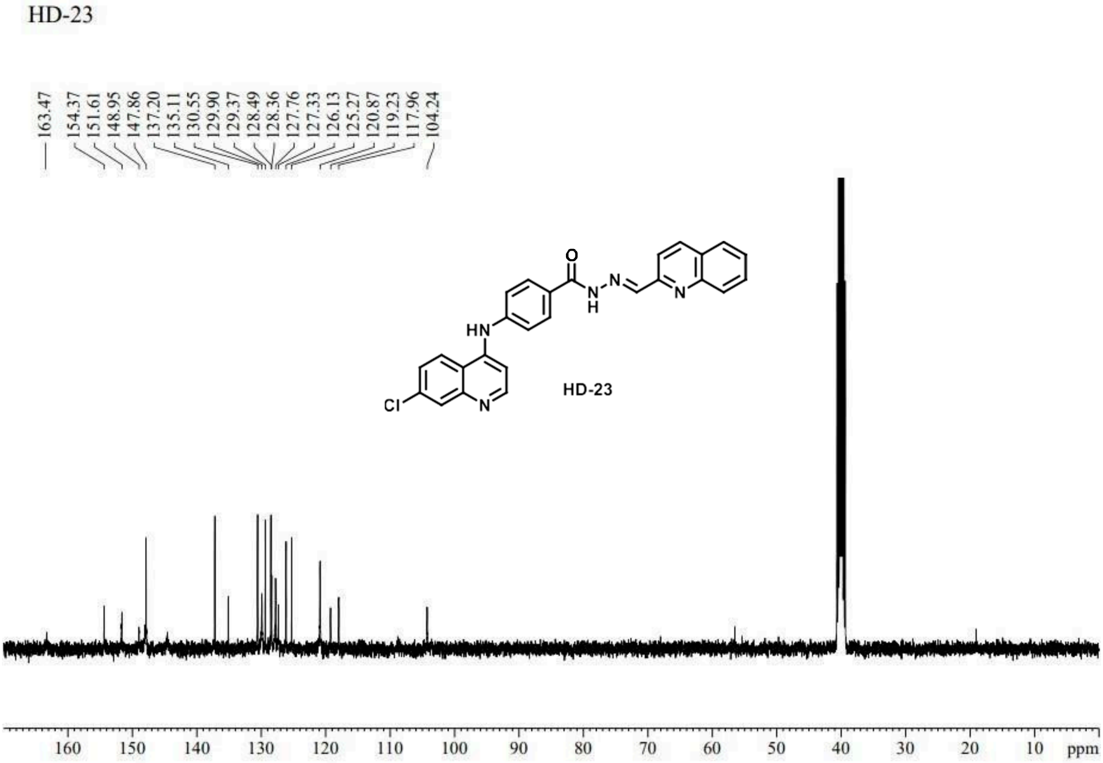

Figure S68: ^{13}C NMR spectrum of **HD23.**

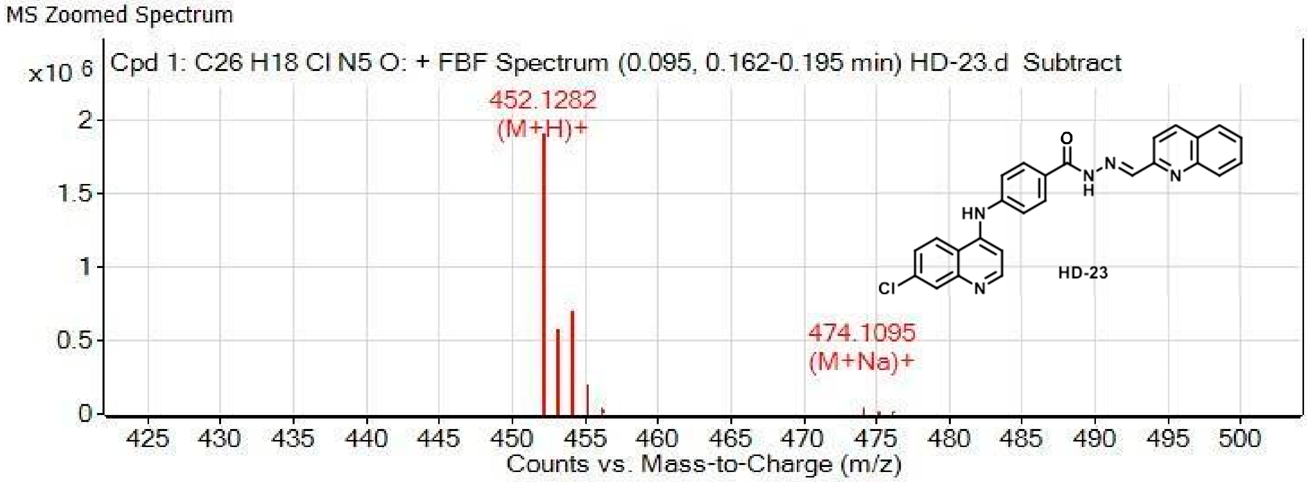

Figure S69: Mass spectrum of **HD23.**

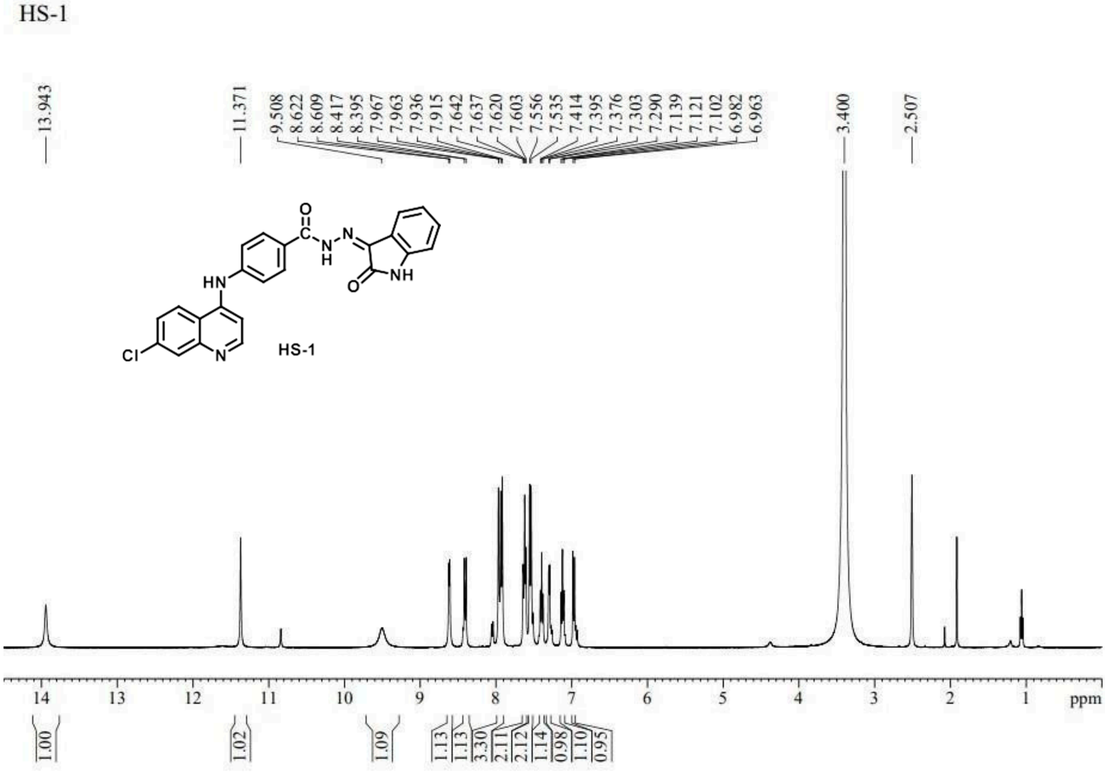

Figure S70: ^{1}H NMR spectrum of **HS1.**

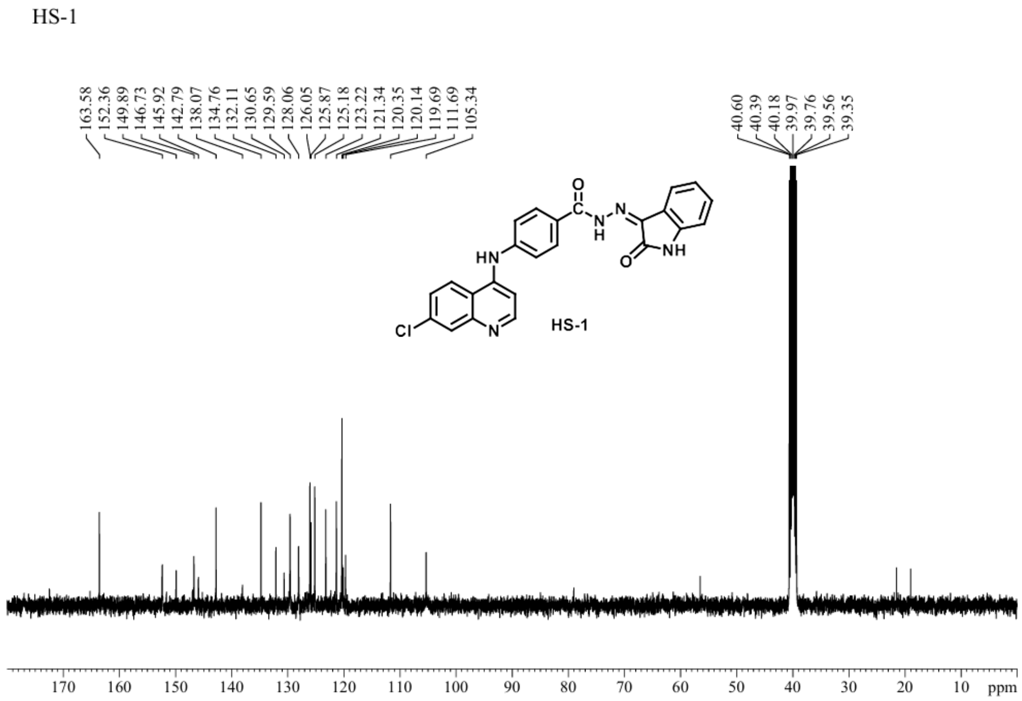

Figure S71: ^{13}C NMR spectrum of **HS1.**

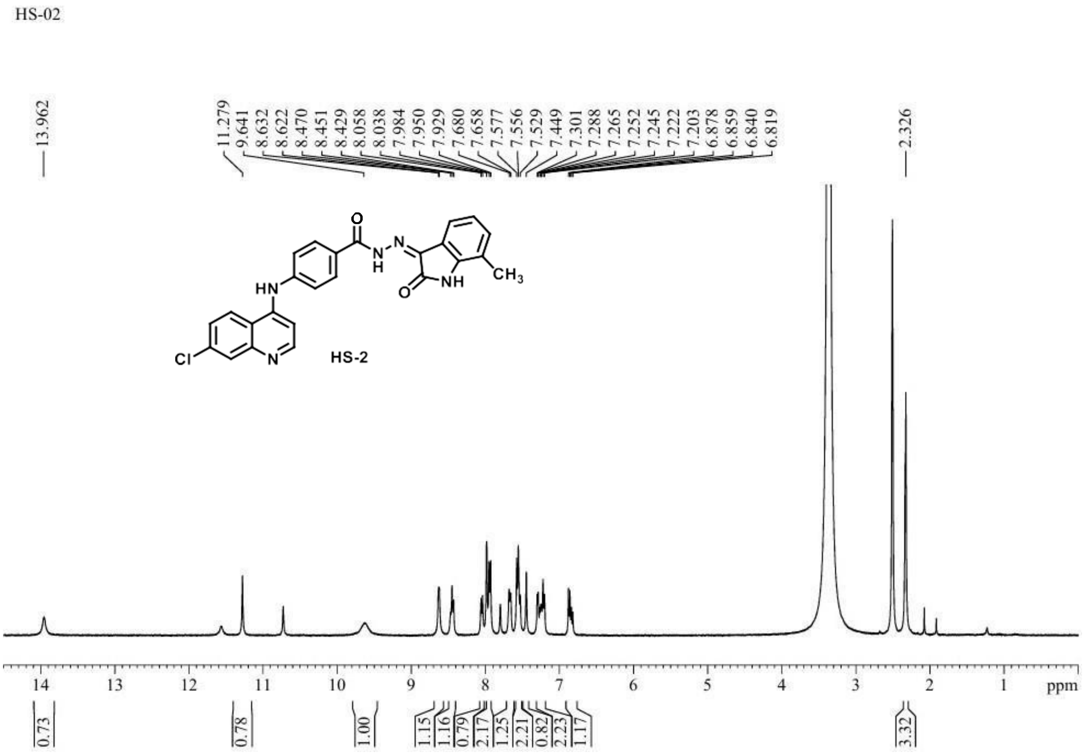

Figure S72: ^{1}H NMR spectrum of **HS2.**

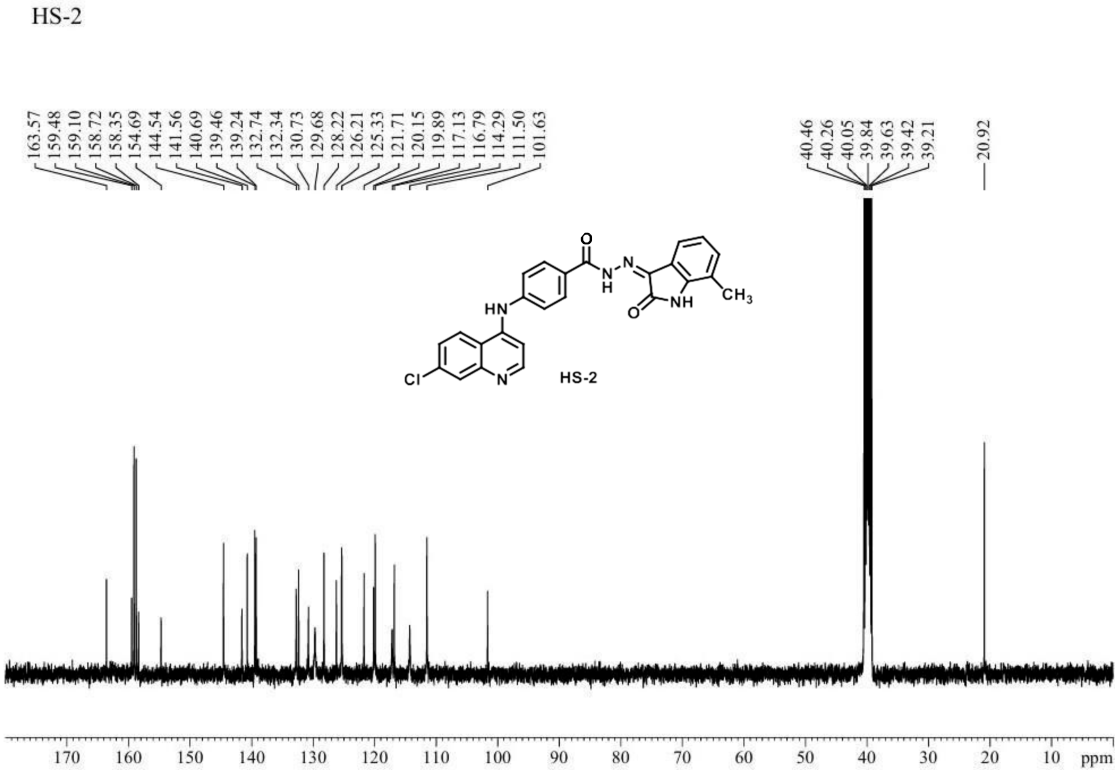

Figure S73: ^{13}C NMR spectrum of **HS2.**

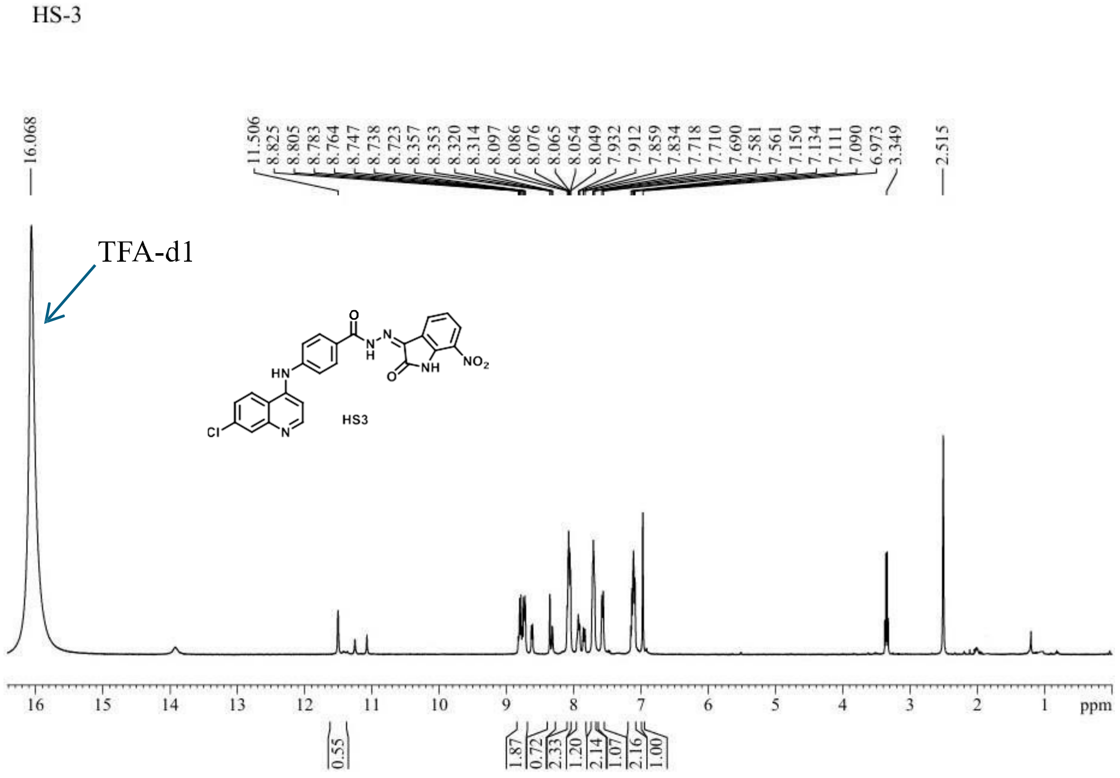

Figure S74: ^{1}H NMR spectrum of **HS3.**

HS-3

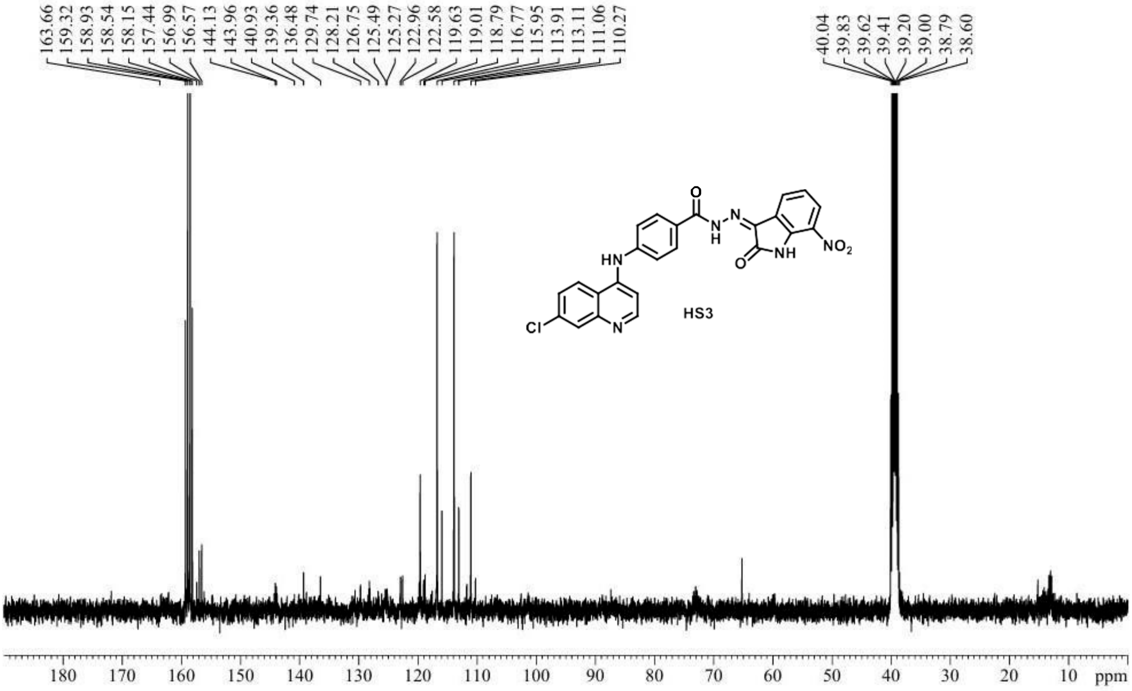

Figure S75: ^{13}C NMR spectrum of **HS3**.

HS-4

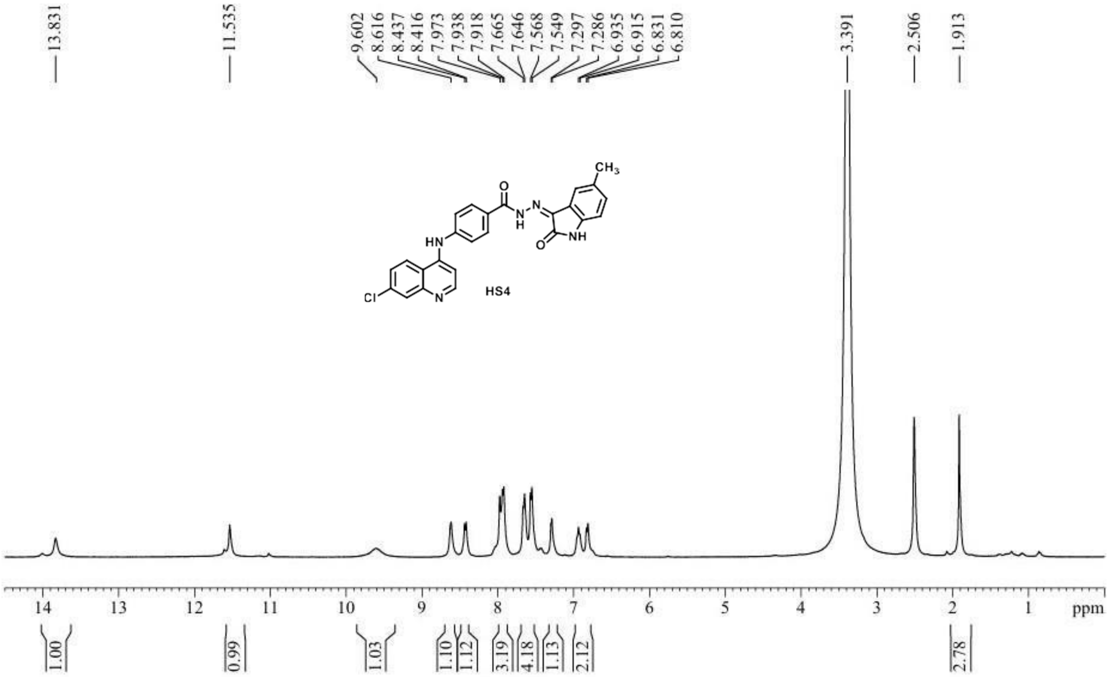

Figure S76: ^{1}H NMR spectrum of **HS4.**

HS-4

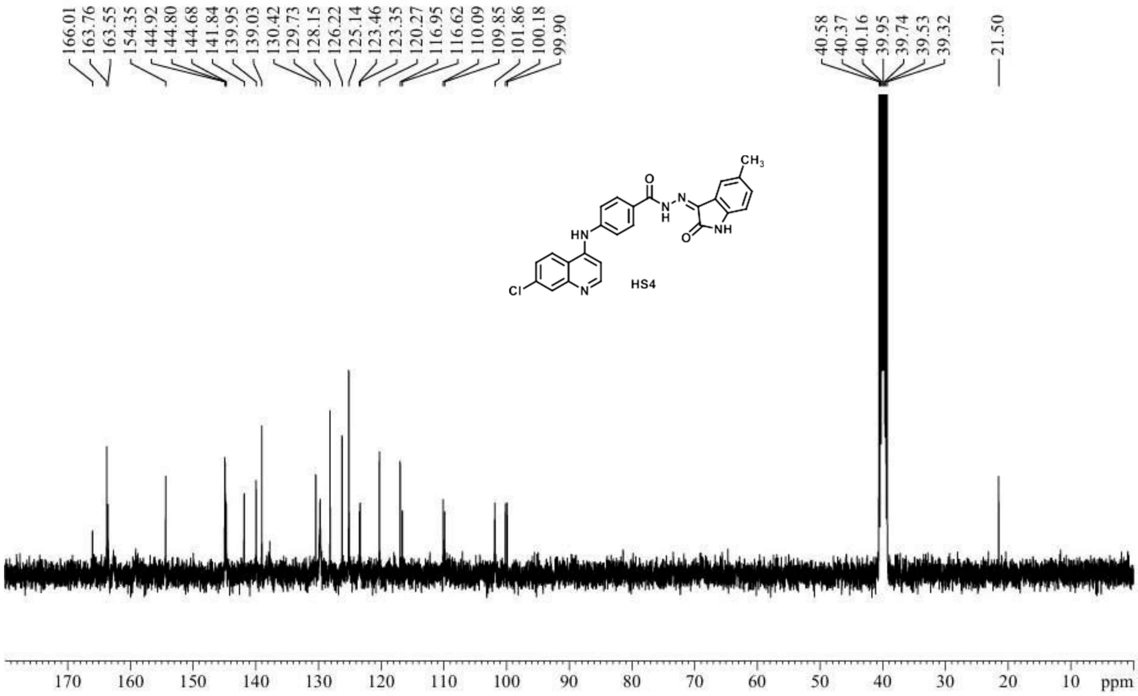

Figure S77: ^{13}C NMR spectrum of **HS4.**

HS-5

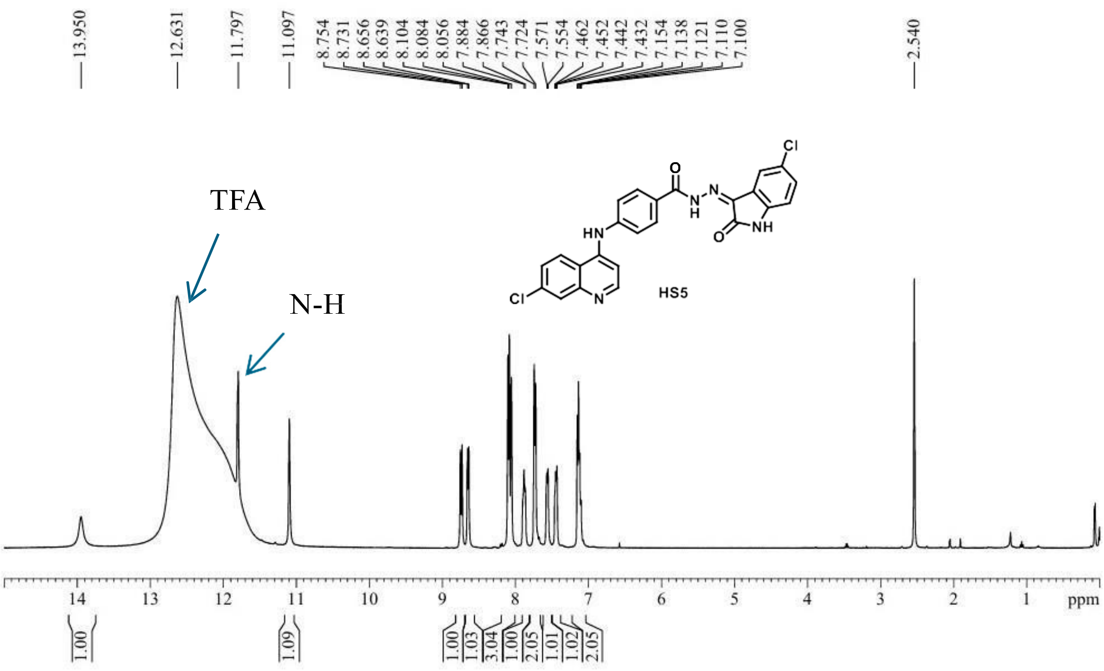

Figure S78: ^{1}H NMR spectrum of **HS5.**

HS-5

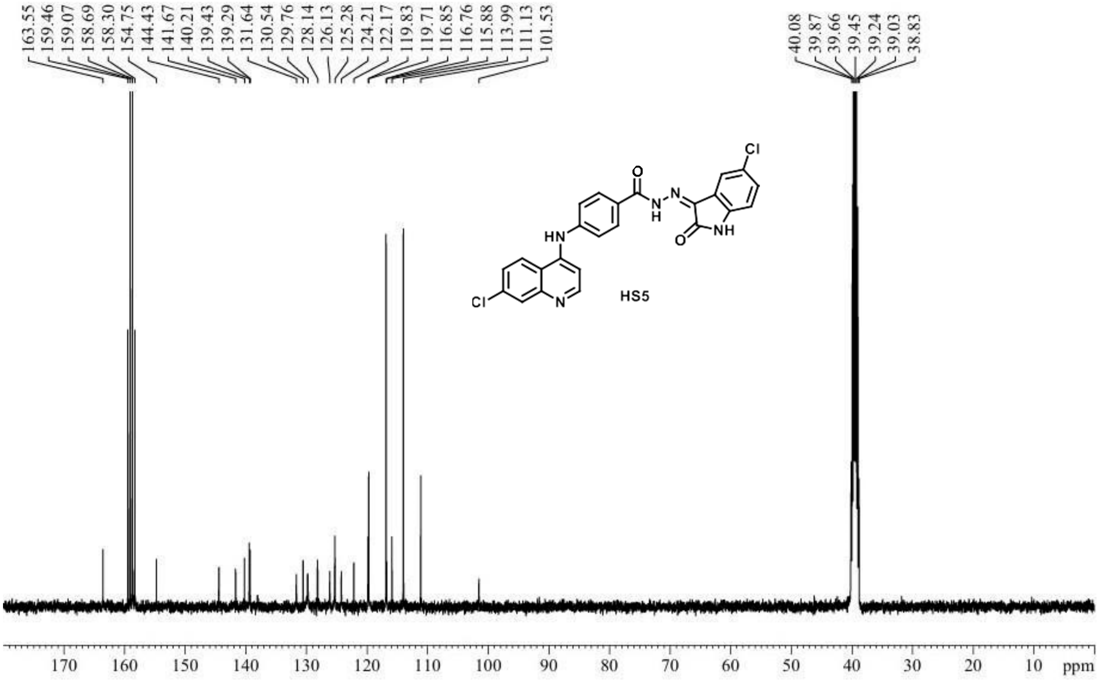

Figure S79: ^{13}C NMR spectrum of **HS5.**

HS-8

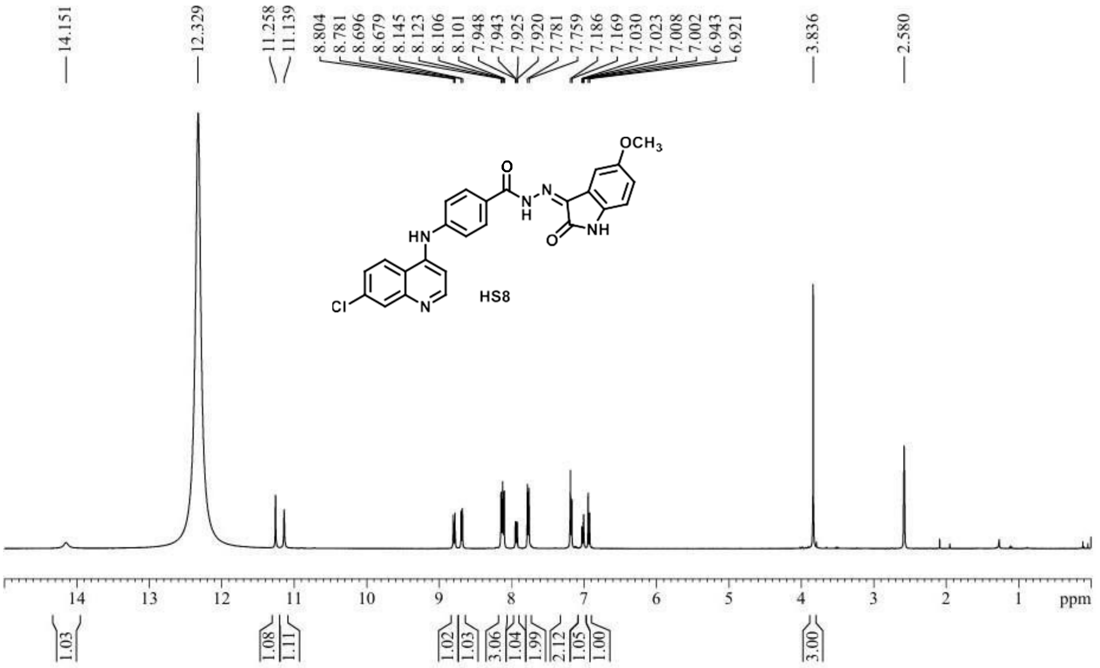

Figure S80: ^{1}H NMR spectrum of **HS8.**

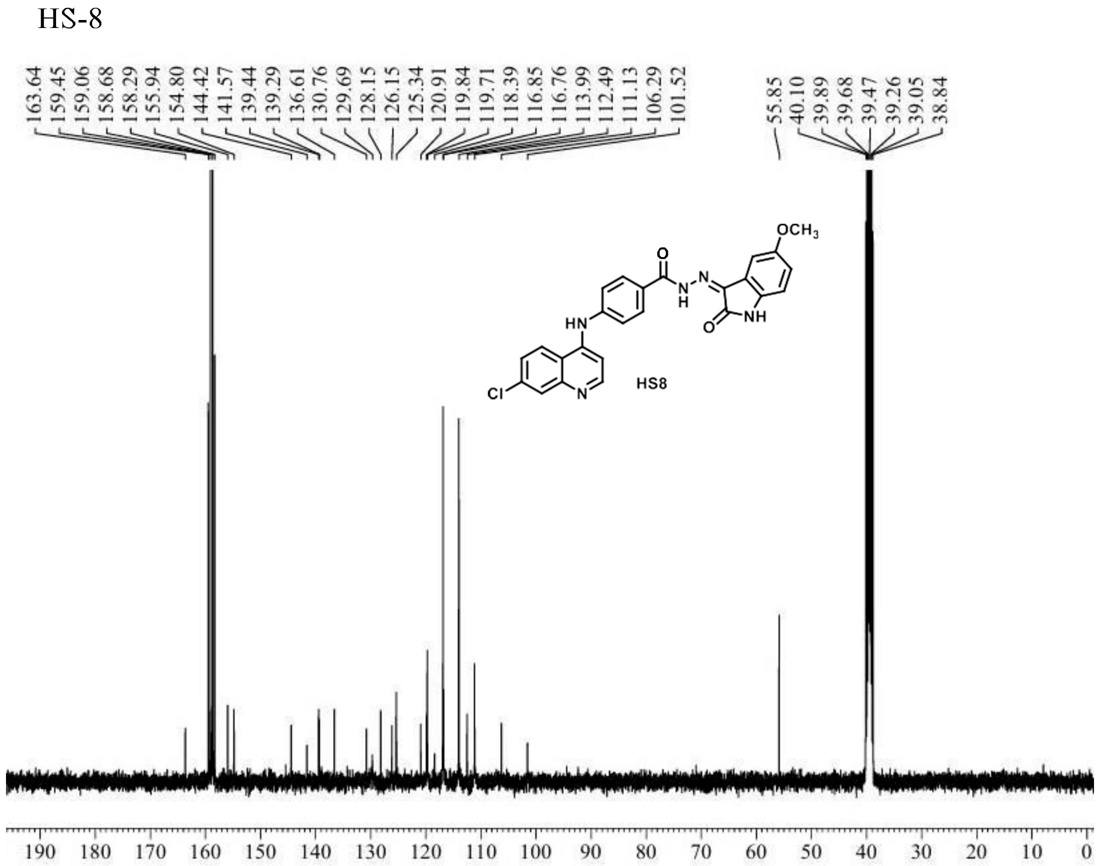

Figure S81: ^{13}C NMR spectrum of **HS8.**

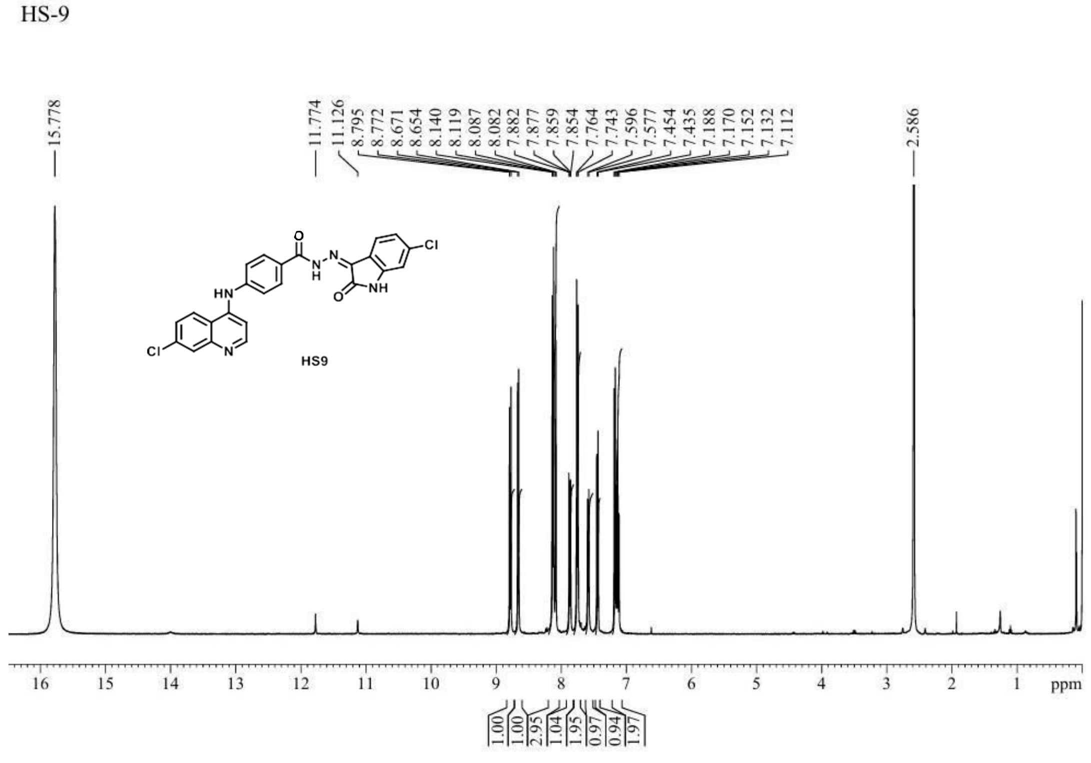

Figure S82: ^{1}H NMR spectrum of **HS9.**

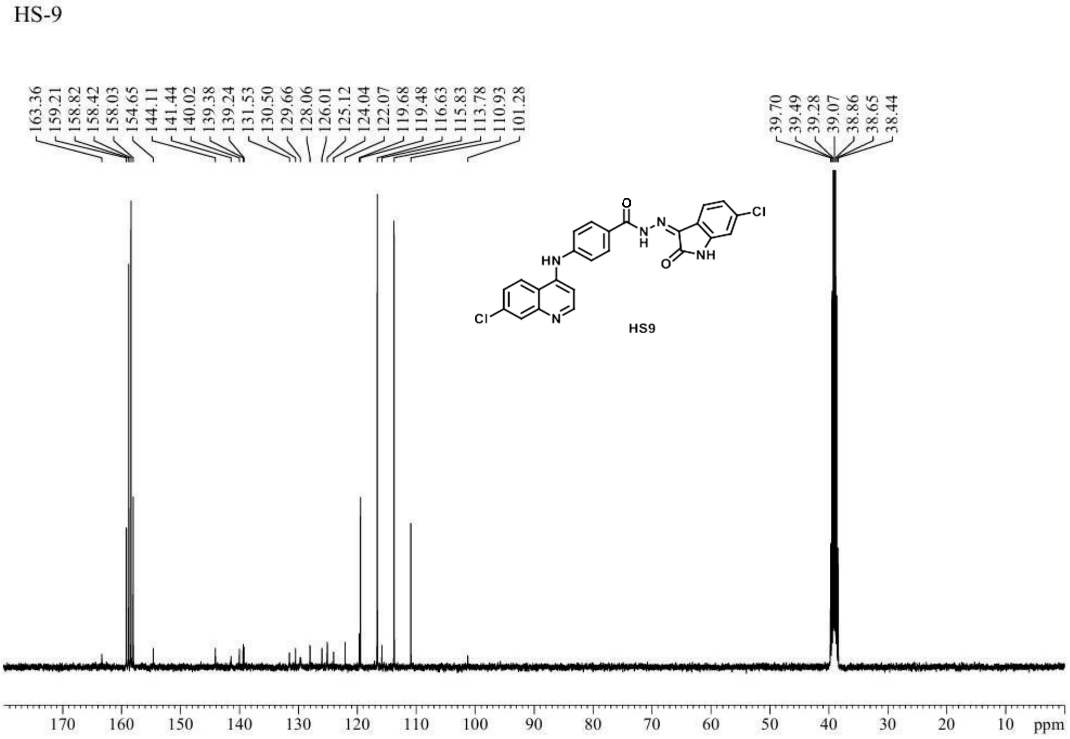

Figure S83: ^{13}C NMR spectrum of **HS9.**

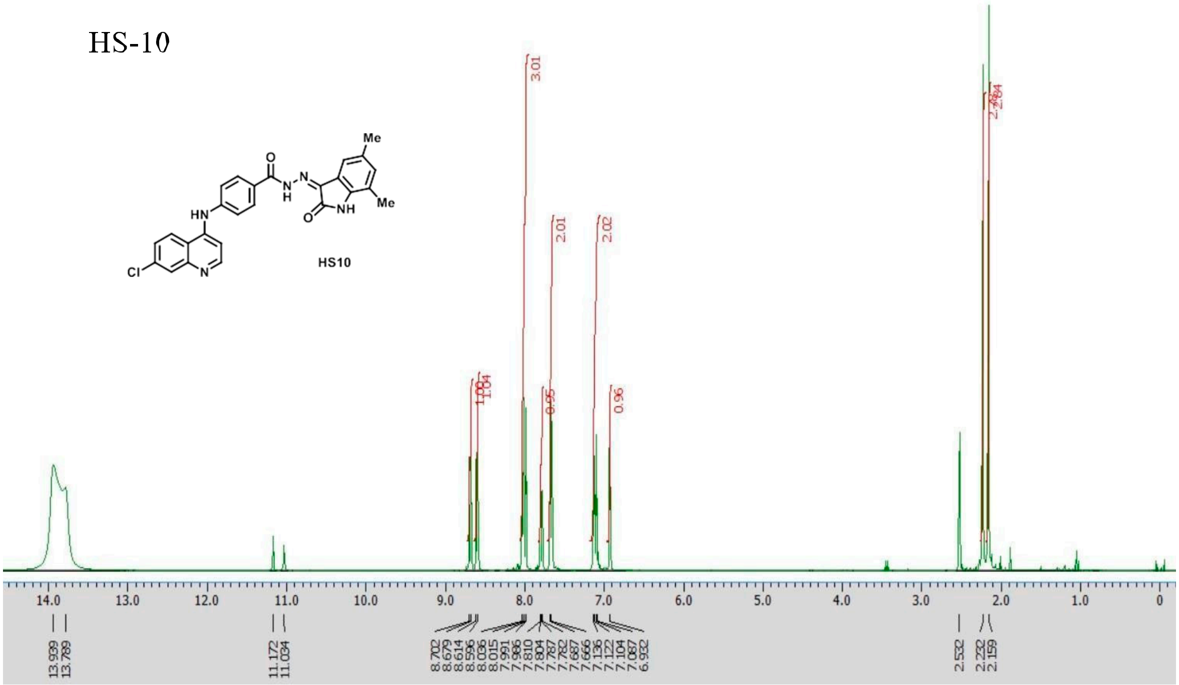

Figure S84: ^{1}H NMR spectrum of **HS10.**

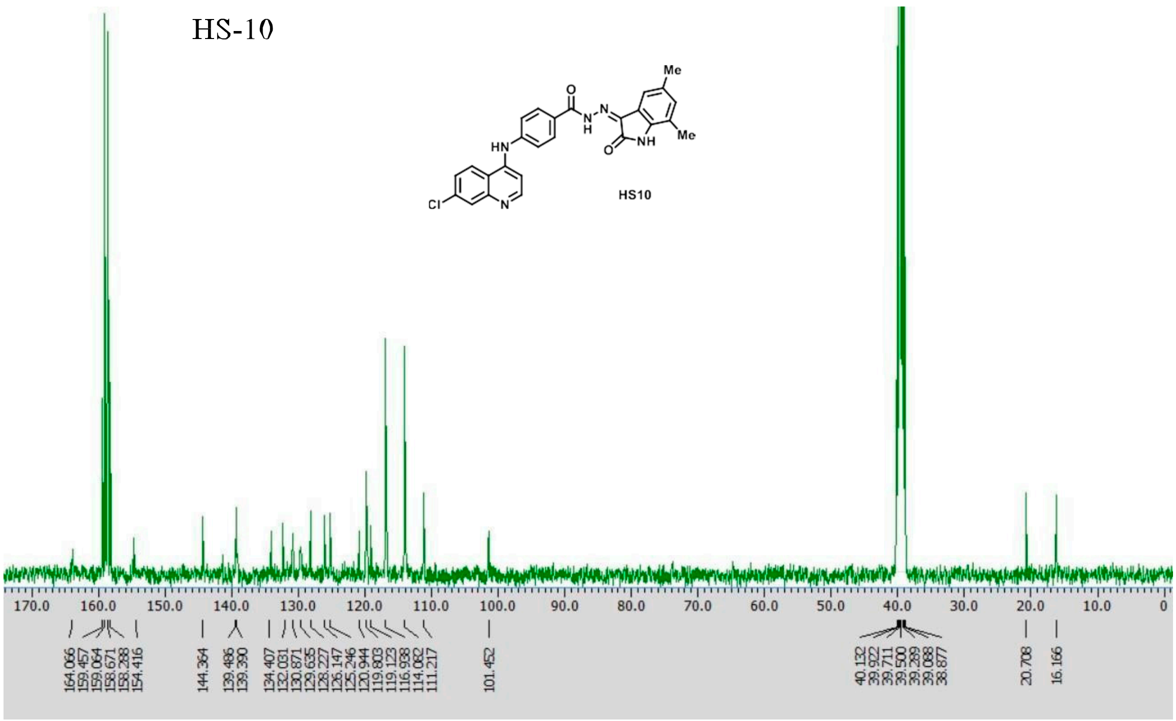

Figure S85: ^{13}C NMR spectrum of **HS10.**

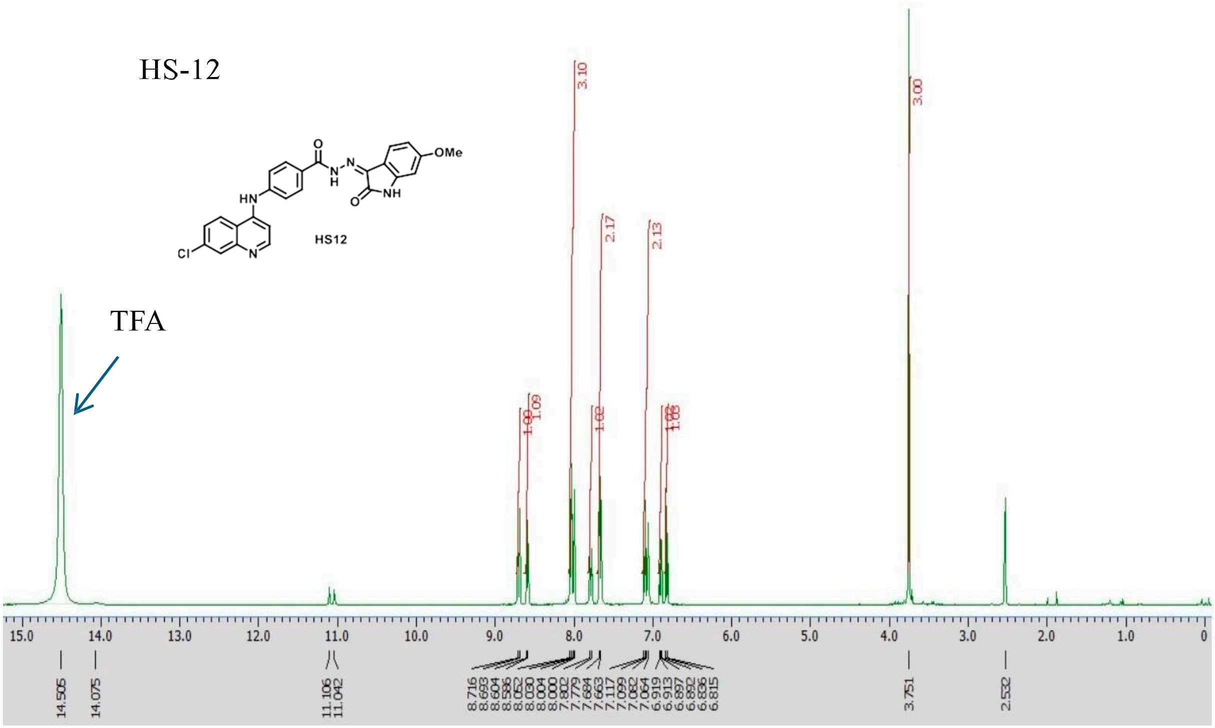

Figure S86: ^{1}H NMR spectrum of **HS12.**

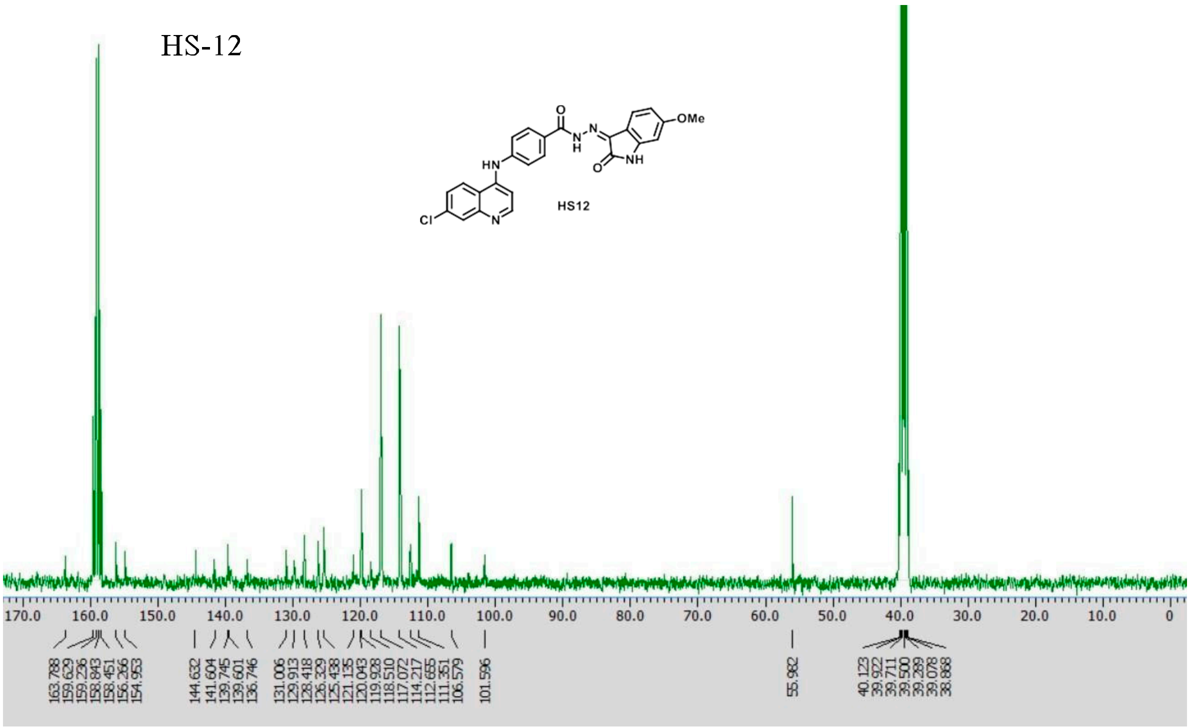

Figure S87: ^{13}C NMR spectrum of **HS12.**
